# Spatiotemporal multi-omics analysis uncovers NAD-dependent immunosuppressive niche triggering early gastric cancer

**DOI:** 10.1038/s41392-025-02390-w

**Published:** 2025-09-22

**Authors:** Pingting Gao, Chunman Zuo, Wei Yuan, Jiabin Cai, Xiaoqiang Chai, Ruijie Gong, Jia Yu, Lu Yao, Wei Su, Zuqiang Liu, Shengli Lin, Yun Wang, Mingyan Cai, Lili Ma, Quanlin Li, Pinghong Zhou

**Affiliations:** 1 Endoscopy Center and Endoscopy Research Institute, Zhongshan Hospital, Fudan University, Shanghai, China; 2 Innovation Center for Evolutionary Synthetic Biology, School of Life Sciences, Sun Yat-sen University, Guangzhou, China; 3 Pathology Department, Zhongshan Hospital, Fudan University, Shanghai, China; 4 Department of Liver Surgery and Transplantation, Liver Cancer Institute, Zhongshan Hospital, Key Laboratory of Carcinogenesis and Cancer Invasion of Ministry of Education, Key Laboratory of Medical Epigenetics and Metabolism, Fudan University, Shanghai, China

**Keywords:** Gastrointestinal cancer, Oncogenes

## Abstract

Understanding the cellular origins and early evolutionary dynamics that drive the initiation of carcinogenesis is critical to advancing early detection and prevention strategies. By characterizing key molecular, cellular and niche events at the precancerous tipping point of early gastric cancer (EGC), we aimed to develop more precise screening tools and design targeted interventions to prevent malignant transformation at this stage. We utilized our AI models to integrate spatial multimodal data from nine EGC endoscopic submucosal dissection (ESD) samples (covering sequential stages from normal to cancer), construct a spatial-temporal profile of disease progression, and identify a critical tipping point (PMC_P) characterized by an immune-suppressive microenvironment during early cancer development. At this stage, inflammatory pit mucous cells with stemness (PMC_2) interact with fibroblasts via *NAMPT*$\longrightarrow$*ITGA5/ITGB1* and with macrophages via *AREG*$\longrightarrow$*EGFR/ERBB2* signaling, fostering cancer initiation. We established gastric precancerous cell lines and organoids to demonstrate that *NAMPT* and *AREG* promote cellular proliferation in vitro. Furthermore, in the transgenic CEA-SV40 mouse model, targeting *AREG* and/or *NAMPT* disrupted key cell interactions, inhibited the JAK-STAT, MAPK, and NFκB pathways, and reduced PD-L1 expression, which was also confirmed by western blot in vitro. These interventions delayed disease progression, reversed the immunosuppressive microenvironment, and prevented malignant transformation. Clinical validation was conducted using endoscopically resected EGC specimens. Our study provides a precise spatiotemporal depiction of EGC development and identifies novel diagnostic markers and therapeutic targets for early intervention.

## Introduction

Gastric cancer (GC) is the fifth most common malignancy globally and ranks as the third leading cause of cancer-related mortality worldwide,^1^ characterized by its complexity, involving multiple stages and diverse etiological factors. According to the Correa cascade model (normal → chronic gastritis → atrophic gastritis → intestinal metaplasia → dysplasia → carcinoma), the development of intestinal-type GC, which constitutes approximately 80% of all GC cases, follows a sequential progression.^2,3^ Intestinal metaplasia (IM) is recognized as a critical precursor in the carcinogenesis of early GC (EGC), representing a pivotal transition point from benign to malignant states.^4^ Patients diagnosed with chronic atrophic gastritis or IM are known to have an elevated annual risk of progression to GC, estimated at 0.1% and 0.25%, respectively.^5,6^ Additionally, spasmolytic polypeptide-expressing metaplasia has been identified as a significant variant of metaplasia associated with GC progression.^7^ Despite substantial advances in understanding the histopathological features and molecular underpinnings of these precancerous changes, there remains a profound lack of clinically effective strategies to prevent or reverse these conditions, underscoring a critical and persistent gap in early intervention and prevention.^8^

Previous multi-omics studies on human GC have primarily utilized samples from normal gastric mucosa,^9^ aiming to identify potential markers through lineage tracing, or IM,^10^ employing targeted DNA sequencing to uncover associated driver genes. Researchers have also extensively explored advanced primary and metastatic GC,^11–14^ including peritoneal carcinomatosis,^12^ malignant cells isolated from patient ascites.^15^ Despite these substantial efforts on defining the endpoints (advanced cancer) and some key waypoints (like IM) of gastric carcinogenesis, investigations into the very genesis and early evolution of EGC itself—specifically the dynamic molecular and cellular processes orchestrating the transition from established pre-cancer (e.g., IM) to incipient malignancy—remain remarkably scarce and pose unique technical and biological challenges. Consequently, elucidating the mechanisms governing EGC onset is paramount for developing rational strategies to intercept carcinogenesis at its earliest, potentially reversible stages, offering a more cost-effective and life-saving approach than managing advanced cancer.

The initiation of EGC is driven by subtle cellular and molecular alterations that are easily obscured by background inflammation or metaplastic changes, rendering them difficult to detect.^16,17^ This challenge is further exacerbated by the lack of high-quality, spatially resolved, and well-annotated clinical specimens that capture the full continuum from pre-cancerous lesions to overt malignancy within the same individual. Most existing studies attempt to reconstruct the gastric carcinogenic cascade by assembling biopsies from spatially and temporally distinct pathological stages—such as normal mucosa, gastritis, IM, and cancer—often derived from different patient cohorts.^18,19^ However, this cross-sectional approach introduces significant inter-patient variability, fragmenting the landscape of tumor evolution and impeding efforts to construct a coherent, continuous atlas of EGC progression. As a result, the precise molecular and spatial dynamics governing the transition from normal gastric epithelium to malignant transformation remain poorly defined and represent a critical unresolved question in the field.

In this study, we employed AI-enabled single-cell RNA-seq (scRNA-seq) and spatial transcriptomics (ST) analyses of ESD specimens from nine patients with EGC. These ESD specimens capture spatially continuous regions—from normal mucosa through IM and the peri-tumoral interface to the tumor core—within the same patient. We defined the transition zone as the PMC_P region, which is spatially proximal to the tumor and distinct from, yet potentially evolutionarily linked to, the adjacent IM region. Our comprehensive analysis revealed several transformative findings in PMC_P region: (1) we identified a novel population of tumor-initiating inflammatory pit mucous cells endowed with stemness properties, termed PMC_2^20,21^; (2) we defined a specific gene set promoting EGC initiation, which correlates with shorter survival time; (3) we identified a distinctive network of ligand–receptor interactions within the PMC_P niche—including *AREG* $\longrightarrow$ *EGFR/ERBB* and *NAMPT* $\longrightarrow$ *ITGA5/ITGB1*—that recruit specific macrophage subsets and activate fibroblasts. These interactions converge to amplify key oncogenic pathways such as STAT1, MAPK, and NF-κB, revealing a previously unrecognized signaling axis essential for EGC initiation; (4) we observed PD-L1 upregulation in the PMC_P region, contributing to an immune-suppressive microenvironment that supports early tumor cell survival and immune evasion; and (5) we validated these findings through a comprehensive series of in vitro and in vivo experiments, including gastric precancerous cell lines, patient-derived organoids, longitudinal ESD specimen analysis, and the CEA-SV40 transgenic mouse model of spontaneous gastric carcinogenesis. Drug intervention studies using *NAMPT* and *AREG* analogs and antagonists further confirmed the therapeutic relevance of these targets. Together, our data and approach offer a valuable resource for elucidating the spatiotemporal dynamics of EGC and uncovering actionable targets for its prevention.

## Results

### Spatial-temporal atlas of EGC in ESD specimens

**Fig. 1:**
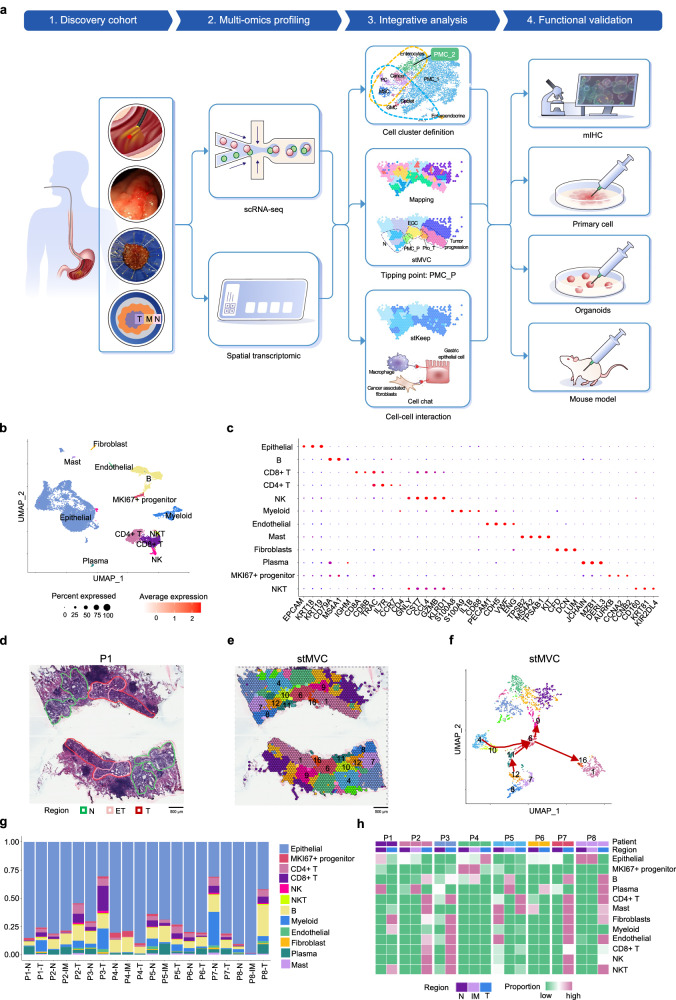
Spatial-temporal heterogeneity of early gastric lesions encompassing normal, intestinal metaplasia (IM), and tumor (T) regions. **a** Schematic overview of the experimental workflow. Fresh ESD samples containing adjacent normal (N), intestinal metaplasia (IM), and tumor (T) tissues were obtained from nine patients. **b** UMAP visualization of single-cell RNA sequencing (scRNA-seq) data showing 12 major cell types. **c** Dot plot illustrating the expression of canonical marker genes across identified cell types. Dot size represents the percentage of cells expressing each gene; color scale indicates the average normalized expression level. **d** H&E-stained spatial transcriptomics (ST) section from patient P1, with histologically annotated normal (N), early tumor (ET), and tumor (T) regions. **e** Spatial domains identified by the stMVC algorithm based on ST data from patient P1. **f** UMAP visualization of spatial domains identified by stMVC in ST data of patient P1. **g** The bar chart showing the proportion of 12 cell types identified from scRNA-seq across each histological in the ST data from eight patients. **h** The heatmap displaying the enrichment of 12 cell types identified from scRNA-seq across 20 histologically annotated regions in the ST data from eight patients

To comprehensively elucidate the sequential processes of EGC, with a particular focus on the cellular composition and spatial organization of the precancerous state and its role in EGC initiation in human, we collected ESD specimens. These specimens included both EGCs of the stage T1 (T), precancerous state (including IM, mild to moderate dysplasia), and matched normal areas (N) (Fig. 1a). In total, 9 tissue samples from 9 individuals without any prior treatment met the criteria, of which 8 specimens were analyzed using the 10X Genomics Visium ST technology (P1-P8),^22,23^ 3 paired tissues (P4-P6) and 1 unpaired specimen (P9) were analyzed by scRNA-seq (Supplementary Fig. 1a and Tables 1–3).

To clarify the heterogeneous cell populations within our interested cellular niches, we analyzed scRNA-seq profiles from adjacent site-matched regions of four patients, yielding a total of 16,839 cells (Supplementary Fig. 1b and Tables 3, 4). To define the cellular composition of the tumor microenvironment (TME) in EGC, we performed graph-based clustering analysis on these cells, identifying 12 distinct cell types based on known marker genes or specific genes uniquely expressed by one cell type. These cell types included epithelial cell, B cell, CD4+ T cell, CD8+ T cell, NK, NKT, myeloid cell, endothelial cell, mast cell, fibroblast cell, plasma cell, *MKI67*+ progenitor cell (Fig. 1b, c, and Supplementary Tables 4–6). Moreover, immunofluorescence staining of classical markers validated the heterogeneous infiltration of these cell types throughout EGC development (Supplementary Fig. 1c, d), underscoring the complexity and dynamic nature of TME.

In the ST analysis, each section containing two continuous slices includes an average of approximately 2500 spots. Each spot, with a diameter of 55 μm, encompasses about 8–20 cells, within the capture area, facilitating the observation of the sequential pathological process from normal to tumor (6.5 mm × 6.5 mm). A total of 20,063 spots from eight patients were sequenced, with a median of approximately 6500 unique molecular identifiers and 2600 genes (Supplementary Table 2). The H&E image areas of each ST data were labeled by the pathologists, and a total of 20 variable histological regions were identified (Fig. 1d and Supplementary Fig. 1e). To explore intra-tumoral heterogeneity, we applied our previously developed AI model stMVC,^24^ which integrates gene expression, spatial location, H&E images, and histological annotations. stMVC employs multi-view graph learning to derive spatial domain embeddings that capture both structural and temporal features of disease progression. Our analysis showed that stMVC can delineate the developmental trajectories of various spatial domains. For instance, in P1, we observed a trajectory from normal tissue to cancer: cluster 12/8 $\longrightarrow$ cluster 11 $\longrightarrow$ cluster 6 $\longrightarrow {\rm{cluster}}1$6 $\longrightarrow \,{\rm{cluster}}$ 1, with cluster 11 representing a transited state between IM and EGC tissues. Similar patterns were observed in the other seven patients (Fig. 1d–f and Supplementary Fig. 1f).

The spatial distribution of classical marker genes of various cell types within the N, IM, and T regions across all patients illustrates the heterogeneity of cellular niches (Fig. 1g). Using GraphST^25^ to estimate the proportions of 12 different cell types for each spot, we noted that all major cell types are present in the tumor, adjacent IM, and normal regions of the eight patients. However, the degree of infiltration of these cell types varies considerably among the different regions, suggesting diverse roles of these cell types at different stages of EGC progression (Fig. 1h).

### Epithelial cell subtypes in the progression of EGC

**Fig. 2:**
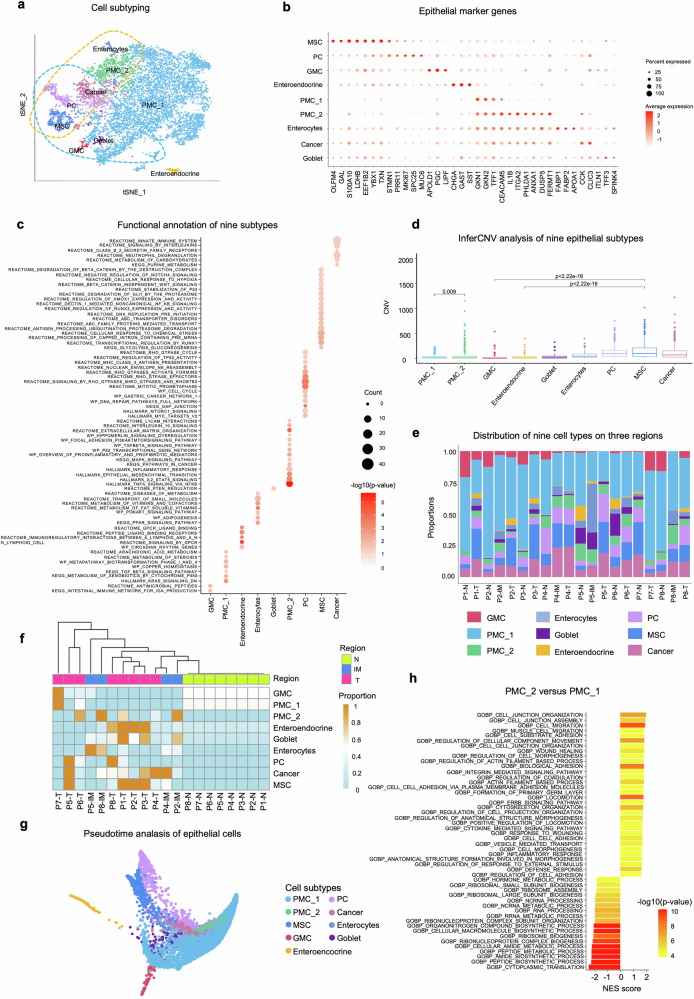
Heterogeneity of epithelial cell subtypes. **a** tSNE plot illustrating nine epithelial cell subtypes identified from scRNA-seq data. **b** Dot plot showing the expression levels of marker genes in each subtype. Dot size and color indicate the percentage and mean expression level of each gene. **c** Functional annotation of nine subtypes. **d** Boxplot displaying large-scale copy number variations (CNVs) in six subtypes (goblet, enterocytes, PMC_2, PC, MSC, and cancer) compared to PMC_1, GMC, and enteroendocrine subtypes. Each color indicates one subtype. **e** Distribution of the nine cell subtypes across N, IM, and T regions in the ST data from each patient. **f** Unsupervised clustering analysis based on the proportions of epithelial subtypes across normal, IM, and cancer regions. **g** Pseudotime trajectory analysis of epithelial cells derived from scRNA-seq data from all single-cell samples. **h** Functional analysis comparing PMC_2 to PMC_1

Upon performing sub-clustering analysis on all 10,474 epithelial cells, we identified nine distinct subtypes, most of which were characterized by known markers (Fig. 2a, b, and Supplementary Fig. 2a). These subtypes include antral basal gland mucous cells (GMC, expressing *MUC6*, *PGC*, and *LIPF*), pit mucous cells (PMC_1, marked by *GKN1*, and *TFF1*), enteroendocrine cells (*CHGA*, *GAST*, and *SST*), enterocytes (*FABP1*/*2* and *APOA1*), goblet cells (*ILTN1*, *TFF3*, and *SPINK4*), proliferative cells (PC, expressing *PRR11*, and *MKI67*). Additional clusters were identified as cancer cells (*CCK*, and *CLIC3*), metaplastic stem-like cells (MSC, defined by *OLFM4*, and *GAL*), and PMC_2 cells, which uniquely exhibited elevated expression of the inflammatory signal *IL1B* compared to PMC_1, along with high expression of *ITGA2* and stem cell-associated markers such as *PHLDA1*.^26^

Each subtype was associated with specific functional pathways. GMCs were linked to the intestinal immune network for IgA production, while PMC_1 cells participated in TGF-β signaling and steroid metabolism. Enteroendocrine cells were involved in arachidonic acid metabolism and GPCR ligand binding. Enterocyte cells were associated with PI3K-AKT signaling pathway and goblet cells regulated by PTEN signaling. Notably, PMC_2 cells exhibited activity in interleukin-10 signaling, the TP53 transcriptional network, MAPK signaling, IL2-STAT5 signaling, and TNF-α signaling via NFκB. PC cells were linked to MHC class II antigen presentation, cell cycle, and modulation of *TP53* activity; MSCs exhibited diverse functions including glycolysis and gluconeogenesis, transcriptional regulation by *RUNX1*, response to chemical stress, and *RUNX3* expression regulation. Cancer cells were involved in innate immune responses, signaling by interleukins, class B2 secretion family receptors, neutrophil degranulation, and metabolism of carbohydrates and purines (Fig. 2c).

To investigate whether the different epithelial subtypes exhibit distinct genetic variations, we analyzed large-scale chromosomal copy number variations (CNVs) across nine cell types using inferCNV.^27^ The analysis revealed several key findings: (1) PMC_2 cells display higher levels of CNVs compared to PMC_1 cells, indicating that PMC_2 is more closely associated with cancer; (2) GMC and enteroendocrine cells show relatively lower CNVs compared to cancer and MSC cells. This pattern aligns with their spatial distribution across different pathological regions, where GMCs and enteroendocrine cells are predominantly located in normal regions, while cancer cells and MSCs are found in cancerous regions (Fig. 2d, e). Clustering analysis based on the proportions of epithelial cell subtype across the normal, IM, and cancer regions indicated that the regions with the same histological type tend to exhibit similar cell-type compositions. Notably, both IM and cancer regions display higher heterogeneity compared to normal tissue, and the epithelial subtype proportions in IM are more similar to those in cancer than those in normal regions (Fig. 2f).

In addition, we leveraged DPT algorithm^28^ to estimate the pseudotime of these nine cell types. The trajectory analysis revealed three branching lineages: PMC_1 $\longrightarrow$ PMC_2 $\longrightarrow$ cancer cell, PC $\longrightarrow$ cancer cell, MSC $\longrightarrow$ cancer cell, which is verified by RNA velocity^29^ (Fig. 2g and Supplementary Fig. 2b, c). Compared to PMC_1, PMC_2 shows various biological functions, including cell migration, wound healing, cell-cell adhesion, EMT-related pathways, and multiple signaling pathways such as NFκB-TNFα, KRAS, ERBB, IL2-STAT5, hypoxia, IL1, INFγ, INFα, and integrin-mediated signaling pathways. These pathways may play critical roles in EGC development (Fig. 2h).

### PMC_P, the tipping-point state during EGC initiation

**Fig. 3:**
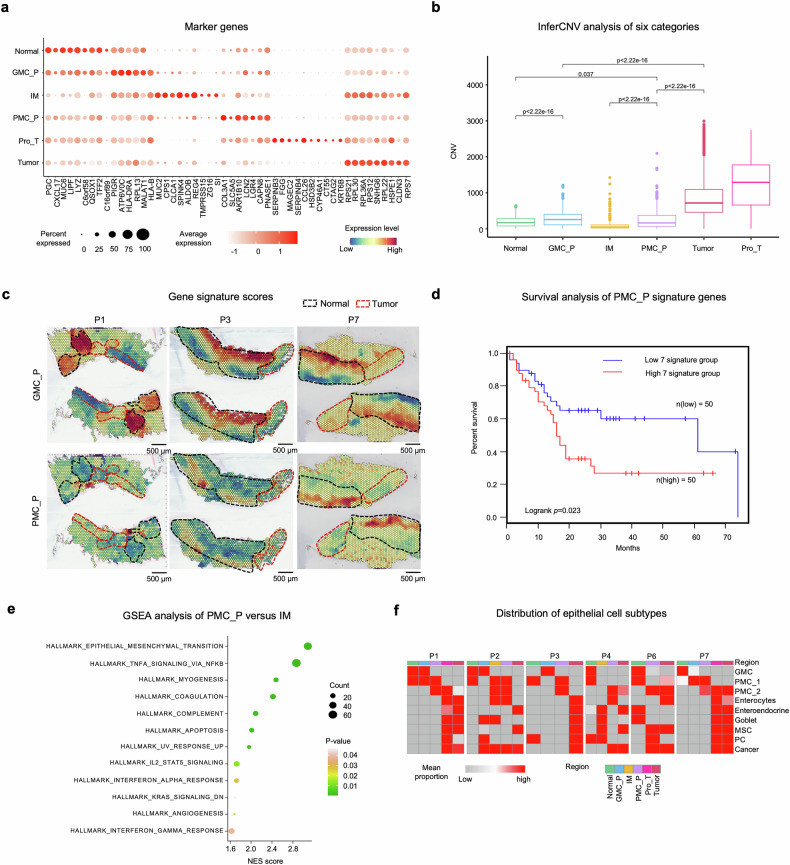
Identification of critical epithelial cell states driving EGC initiation. **a** Dot plot showing the expression of representative marker genes across six spatial epithelial groups identified in ST data. Dot size indicates the percentage of cells expressing each gene; color scale denotes mean normalized expression level. **b** Box plot showing large-scale CNV levels across six spatial groups. CNV levels were normalized across samples; each color represents a different group. **c** Gene signature scores of GMC_P (top) and PMC_P (bottom) marker genes across ST regions in three representative patients (P1, P2, and P3). **d** Overall survival rate of patients based on the average expression of seven signature genes of PMC_P in stomach cancer from the TCGA database by GEPIA2.^83^ **e** Gene set enrichment analysis (GSEA) of differentially expressed genes in PMC_P versus IM. **f** Heatmap showing the proportion of nine epithelial subtypes in six groups of ST data

To identify epithelial cells that are over-enriched during EGC initiation, we classified spatial domains from three epithelial regions (i.e., N, IM, and T) into six categories associated with disease progression using stMVC.^24^ These categories are normal, GMC_P, IM, PMC_P, Pro_T, and tumor, each characterized by distinct gene expression profiles (Fig. 3a). PMC_P and GMC_P, observed as specific regions prior to cancer and in transitional zones from normal to tumor, represent precancerous states.^30^ Pro_T, found in cancer areas, exhibits functions related to tumor progression, such as inflammatory responses (TNF-α signaling via NF-κB and IL-6/JAK/STAT3 signaling), fibrotic/remodeling pathways (epithelial-mesenchymal transition and TGF-β signaling), metabolic reprogramming (cholesterol homeostasis and glycolysis) and cell fate control (apoptosis, TP53 pathway) activation, indicating a complexed pro-tumor state (Supplementary Fig. 2d). This classification aligns with inferred CNVs, which show increasing levels from normal to GMC_P and tumor, and from normal/IM to PMC_P, and tumor (Fig. 3b). Notably, while confirming a known differentiation pathway from GMC to tumor,^19^ we also identified a potential novel pathway from PMC to tumor (Fig. 3c).

**Fig. 4:**
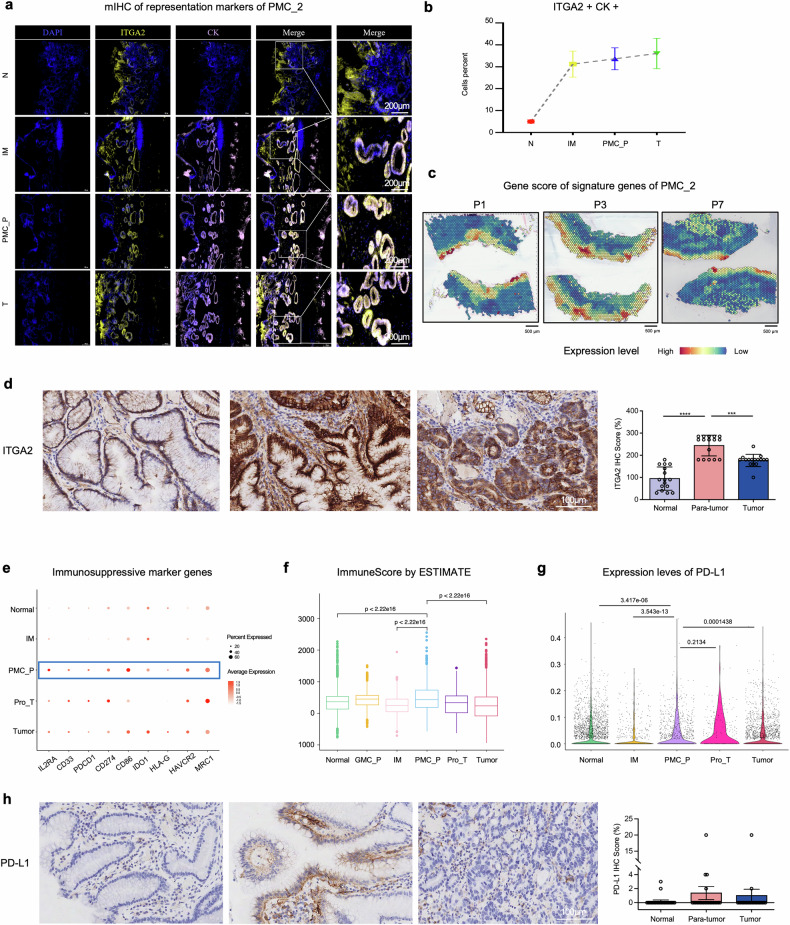
Precancerous PMC_P niche coordinates PMC_2 accumulation and PD-L1-mediated immune suppression. **a** Spatial distribution of PMC_2 markers (CK*/ITGA2*) across these groups via immunofluorescence double labeling (DAPI/CK/*ITGA2*). Co-localization signals (Merge, white) of CK (purple) and *ITGA2* (yellow) show enrichment of PMC_2 cells in the PMC_P region indicating their expansion starting from the precancerous microenvironment. **b** Quantitative analysis further confirms a gradient increase in PMC_2 proportion across progression stages (N < IM < PMC_P < T). **c** Spatial expression of the gene signature score of PMC_2 in three representative patients: P1, P3, and P7. **d** *ITGA2* expression in 21 EGC patients showed significant higher expression level of *ITGA2* in para-tumor regions than other regions. Scale bar: 200 μm. **e** Dot plot showing the expression of classical immunosuppressive marker genes, with *IL2RA*, *CD33*, *PDCD*, *CD274*, *CD86*, *IDO1*, *HLA-G*, *HAVCR2*, and *MRC1* highly expressed in PMC_P region. Dot size indicates detection rate, color scale represents normalized expression. **f** Boxplot of the distribution of immune score (calculated by ESTIMATE) across the six groups showing significantly higher immune score in PMC_P region than normal or tumor. **g** Violin plot showing the expression level of PD-L1 across six groups which is significantly higher in PMC_P region. **h** PD-L1 expression in 21 EGC patients showing higher expression of PD-L1 in para-tumor regions than other regions. Scale bar: 200 μm. ***p < 0.001, ****p < 0.0001

To elucidate the potential role of PMC_P in driving EGC initiation, we employed multiple complementary approaches: (i) PMC_P cells show over-expression of genes such as *COL3A1*, *SLC5A5*, *AKR1B10, LCN2, LGR4*, *CAPN8*, and *RNASE1*, which have been previsouly reported to be associated with GC development^31–35^(Fig. 3a); (ii) the mean expression level of seven representative “initiation-promoting” genes in PMC_P significantly correlates with shorter overall survival, as validated in an independent cohort from the TCGA stomach cancer database (log-rank test, $p-{value}=0.023$) (Fig. 3d); (iii) pathway analysis reveal that PMC_P cells are actively involved in key oncogenic signaling cascades, including apoptosis, EMT, JAK-STAT, and MAPK pathways (Fig. 3e); (iv) comparative analyses of epithelial subtypes across six groups using GraphST uncover distinct patterns: PMC_1 cells are predominantly enriched in normal/IM regions, while PMC_2 cells are significantly more abundant in PMC_P regions (Fig. 3f and Supplementary Fig. 2e), a pattern confirmed by mIHC (Fig. 4a, b). This distribution is consistent with the elevated PMC_2 gene signature scores in PMC_P regions in both ST data (Fig. 4c) and clinical specimens (Fig. 4d and Supplementary Table 7); (v) PMC_P regions are characterized by immunosuppressive microenvironment, as evidenced by the presence of immune checkpoint molecules (*PDCD1*, *CD274*, and *HAVCR2*^36^), co-stimulatory/co-inhibitory molecules (*CD86*^37^), immunosuppressive enzymes (*IDO1*,^38^ *HLA-G*^39^), myeloid cell markers (*CD33*,^40^ *MRC1*^41^), and T-cell regulation molecule (*IL2RA*^42^) within precancerous epithelial regions (Fig. 4e). Moreover, the proportion of immunosuppressive immune cells is significantly higher in PMC_P compared to the other five groups (Fig. 4f and Supplementary Table 8). Furthermore, we observed elevated PD-L1 expression in precancerous epithelial regions, as shown by both ST data (Fig. 4g) and early-stage clinical cancer samples (Fig. 4h). Taken together, these findings identify PMC_2 as a critical transitional epithelial population bridging IM and tumorigenesis, and PMC_P as a pivotal precancerous niche characterized by an immunosuppressive microenvironment that precedes EGC onset.

### Hyperactivation of PMC_2-macrophage/fibroblast crosstalk via *EGFR/ERBB2-AREG* and *NAMPT-ITGA5/ITGB1* in PMC_P

**Fig. 5:**
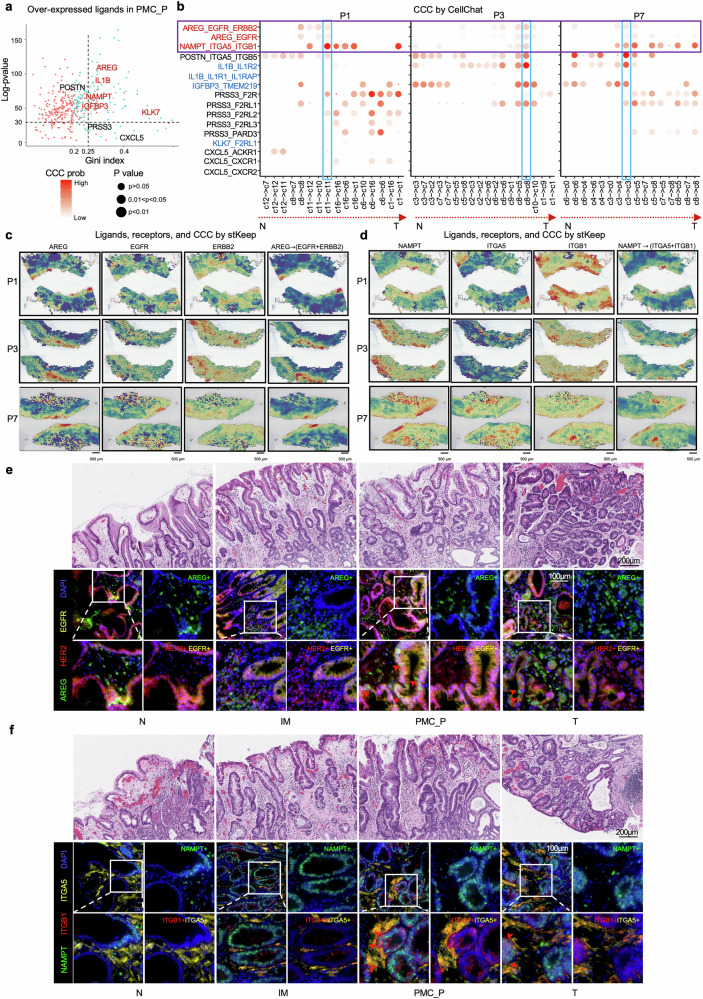
Identification of key cell populations and CCC mechanisms in the initiation of EGC. **a** Scatter plot showing the specificity (Gini index) and difference level ($\log P-{value}$) of over-expressed ligand genes in the PMC_P group. **b** Bubble heatmap displaying mean CCC strength by CellChat for *AREG*, *IL1B*, *NAMPT*, *KLK7*, *IGFBP3*, *PRSS3*, *CXCL5*, and *POSTN* interaction pairs across different domains in three representative patients: P1, P3, and P7. **c**, **d** Spatial expression of the ligand *AREG*, receptors *EGFR* and *Erb-B2* receptor tyrosine kinase 2 (*ERBB2)*, and their corresponding CCC interactions in three representative patients: P1, P3, and P7. **e** H&E plot with four groups: N, IM, PMC_P, and T in the upper panel with a scale bar of 200 µm. mIHC images of the four groups for *AREG* (green), *EGFR* (yellow), and *ERBB2* (red), with a scale bar of 20 µm showed over-expression of these three genes in the PMC_P. Red arrows: *AREG*+ cells and *HER2* + *EGFR*+ cells co-localization. **f** H&E plot with four groups: N, IM, PMC_P, and T in the upper panel with a scale bar of 1 mm. mIHC images of the four groups for *NAMPT* (green), *ITGA5* (yellow), and *ITGB1* (red), with a scale bar of 20 µm, showed over-expression of these three genes in the PMC_P. Red arrows: *ITGB1* + *ITGA5*+cells and *NAMPT*+ cells co-localization

To investigate the potential cell-cell communication (CCC) mechanisms, which involve various signaling pathways that regulate interactions between different cell types linked to EGC initiation, we analyzed genetic differences between PMC_P and five other epithelial cell groups. Using the Gini index program, we identified several secreted protein candidates, including *AREG*, *IL1B*, *NAMPT*, *KLK7*, and *IGFBP3*, which were specifically elevated in PMC_P compared to other groups (Gini index > 0.25, Fig. 5a). To further explore the key proteins involved in cell communication within PMC_P, we utilized CellChat software^43^ and identified that the ligand-receptor pairs (LRPs) of *AREG* $\longrightarrow$ *EGFR*/*ERBB2* and *NAMPT* $\longrightarrow$ *ITGA5*/*ITGB1* predominantly drive the cellular interactions in PMC_P across six patients (Fig. 5b and Supplementary Fig. 3a). These findings were further corroborated using our CCC inference tool stKeep,^44^ which applies contrastive heterogeneous graph learning to infer CCC at the spot level, enabling robust and comparable pattern identification across spatial domains (Fig. 5c, d and Supplementary Fig. 3b). Furthermore, mIHC analysis confirmed that *AREG* and *EGFR*/*ERBB2*, as well as *NAMPT* and *ITGA5*/*ITGB1*, were overexpressed in the PMC_P region (Fig. 5e, f), verified in clinical specimen (Supplementary Fig. 3c and Tables 9, 10) suggesting that these LRPs play a critical role in mediating tumor initiation in the tipping point. We have also analyzed genetic differences between Pro_T region and other regions which also showed *NAMPT* $\longrightarrow \,$*ITGA5*/*ITGB1* active in Pro_T region in 2 patients (Supplementary Fig. 3d, e).

**Fig. 6:**
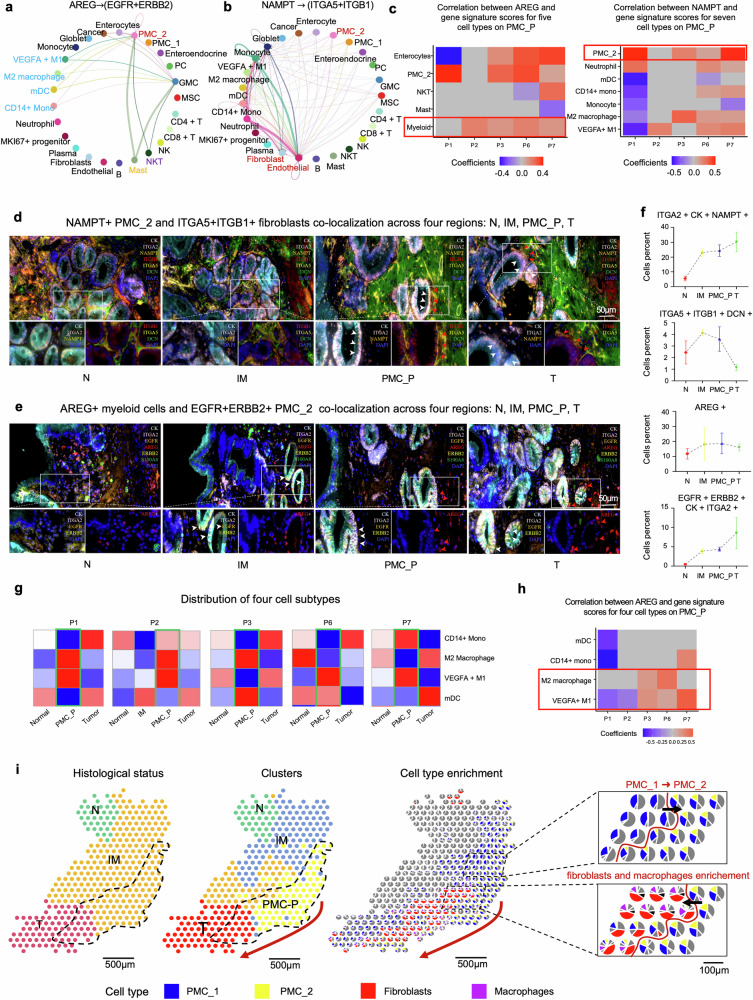
PMC_2 interacts strongly with fibroblasts and macrophages within PMC_P. **a**, **b** Circle plot (based on pan-tissue scRNA-seq across regions) displaying the intercellular communication between different cell types for *AREG* $\longrightarrow$ *EGFR*/*ERBB2*, and *NAMPT* $\longrightarrow$ *ITGA5*/*ITGB1*. **c** Spearman correlation (region-specific analysis-PMC_P only) between *AREG* expression and gene signature scores for enterocytes (*FABP1*, *FABP2*, and *APOA1*), PMC_2 (*CEACAM5*, *ITGA2*, *PHLDA1*, *ANXA1*, *DUSP5*, and *FERMT1*), NKT cells (*CD160*, *KRT81*, and *KIR2DL4*), mast cells (*TPSB2*, *MS4A2*, *TPSAB1*, and *KIT*), and myeloid cells (*S100A8*, *S100A9*, *IL1B*, and *CD68*) (left), *NAMPT* expression and gene signature scores for PMC_2 (*CEACAM5*, *ITGA2*, *PHLDA1*, *ANXA1*, *DUSP5*, and *FERMT1*), neutrophil (*FCGR3B*, *CXCR2*, *IFITM2*, *CMTM2*, and *CSF3R*), mDC (*PPP1R14A*, *FCER1A*, *HLA-DPB1*, and *CD1C*), CD14 mono (*GIMAP7*, *AHNAK*, and *CD36*), monocyte (*CCDC88C*, and *POU2F2*), M2 macrophage (*SLCO2B1*, *C1QC*, *C1QA*, *GPNMB*, and *C1QB*), and *VEGFA* + M1 (*TGM2*, *CLEC5A*, *VEGFA*, and *MCEMP1*) (right). **d** mIHC staining shows ligand-receptor pair *NAMPT* (secreted by PMC_2 marked by *CK* + *ITGA2*+) $\longrightarrow$ *ITGA5/ITGB1* (expressed by fibroblasts marked by *DCN*) across four regions: N, IM, PMC_P, T. The magnified view within the boxed region highlights *NAMPT* + PMC_2 cells (white arrows) and *ITGB1* + *ITGA5*+fibroblast cells (red arrows). Scale bar: 50 μm. **e** mIHC staining illustrates ligand-receptor pair *AREG* (expressed by macrophages marked by *S100A8)* $\longrightarrow$ *EGFR/ERBB2* (expressed by PMC_2 marked by *CK*, and *ITGA2*) across four regions: N, IM, PMC_P, T. The magnified view within the boxed region highlights *EGFR* + *ERBB2* + PMC_2 cells (white arrows) and *AREG*+ macrophages (red arrows). Scale bar: 50 μm. **f** Line charts showing the proportion of *AREG*+ cells, *EGFR* + *ERBB2* + PMC_2 cells, *ITGA5* + *ITGB1*+ fibroblasts, *NAMPT* + PMC_2 cells across four regions: N, IM, PMC_P, T. **g** Distribution of four myeloid cell subtypes showing macrophage as main myeloid cell type in PMC_P region. **h** Spearman correlation between *AREG* and myeloid subclusters: mDC (*PPP1R14A*, *FCER1A*, *HLA-DPB1*, and *CD1C*), CD14 mono (*GIMAP7*, *AHNAK*, and *CD36*), M2 macrophage (*SLCO2B1*, *C1QC*, *C1QA*, *GPNMB*, and *C1QB*), and *VEGFA* + M1 (*TGM2*, *CLEC5A*, *VEGFA*, and *MCEMP1*). **i** Spatial distribution of three histological status (i.e., N, IM, and T), spatial clusters by stMVC (N, IM, PMC_P, and T), distributions of five cell types, and zoomed-in views of the cell type distributions from PMC_P to T

To further investigate which cell types utilize these LRPs, we applied CellChat analysis to our scRNA-seq data. This revealed strong interactions between myeloid cells (e.g., M1 macrophage, M2 macrophage, mDC, *CD14*+ monocytes, mast)/NKT cells and PMC_2/GMC in relation to the *AREG* $\longrightarrow$ *EGFR*/*ERBB2* axis, and between myeloid cells/PMC_2 and stromal cells (e.g., fibroblasts and endothelial cells) via the *NAMPT*$\longrightarrow$*ITGA5*/*ITGB1* axis (Fig. 6a, b and Supplementary Fig. 4a–c). Further analysis of marker distributions (Supplementary Fig. 4d), together with analyses between gene expression and signature scores, clarified the cellular sources of key signaling molecules. These results confirmed that *AREG* is primarily derived from myeloid cells, *NAMPT* from PMC_2, and *ITGA5/ITGB1* from stromal cells (Fig. 6c, Supplementary Fig. 4e), showing robust communication between myeloid cells and PMC_2 via *AREG* $\longrightarrow \,$*EGFR*/*ERBB2*, as well as between PMC_2 and fibroblasts via *NAMPT* $\longrightarrow \,$*ITGA5*/*ITGB1* within the PMC_P region, which was further validated by mIHC (Fig. 6d–f, Supplementary Fig. 4f). Additionally, mIHC showed that (i) PMC_P exhibits higher levels of *ITGA2*, *NAMPT*, *ITGB1*, *ITGA5*, and *DCN*, indicating that *NAMPT* derived from PMC_2 interacts with fibroblasts in PMC_P; and (ii) PMC_P shows over-expression of *S100A8*, *AREG*, *EGFR*, *ERBB2*, and *ITGA2*, suggesting that *AREG* from myeloid cells interacts with PMC_2 in PMC_P. We checked the distribution of four myeloid cell subtypes to further identify cell populations interacting with PMC_2 in PMC_P region and analyzed the correlation between *AREG* expression and gene signature scores for myeloid subtypes. We identified macrophages as the major myeloid cell populations enriched in the PMC_P region, and their presence is significantly correlated with *AREG* expression in patients (Fig. 6g, h).

We then performed population enrichment analysis of four representative regions (N, IM, PMC_P, and T) in patient P4, which revealed a progressive increase in PMC_2 from IM to PMC_P, accompanied by significant infiltration of fibroblasts and macrophages from PMC_P to T (Fig. 6i). These findings highlight the critical role of intercellular interactions among PMC_2, macrophages, and fibroblasts in driving EGC initiation.

### *AREG/NAMPT* promotes malignant transformation in precancerous cell lines and organoids

**Fig. 7:**
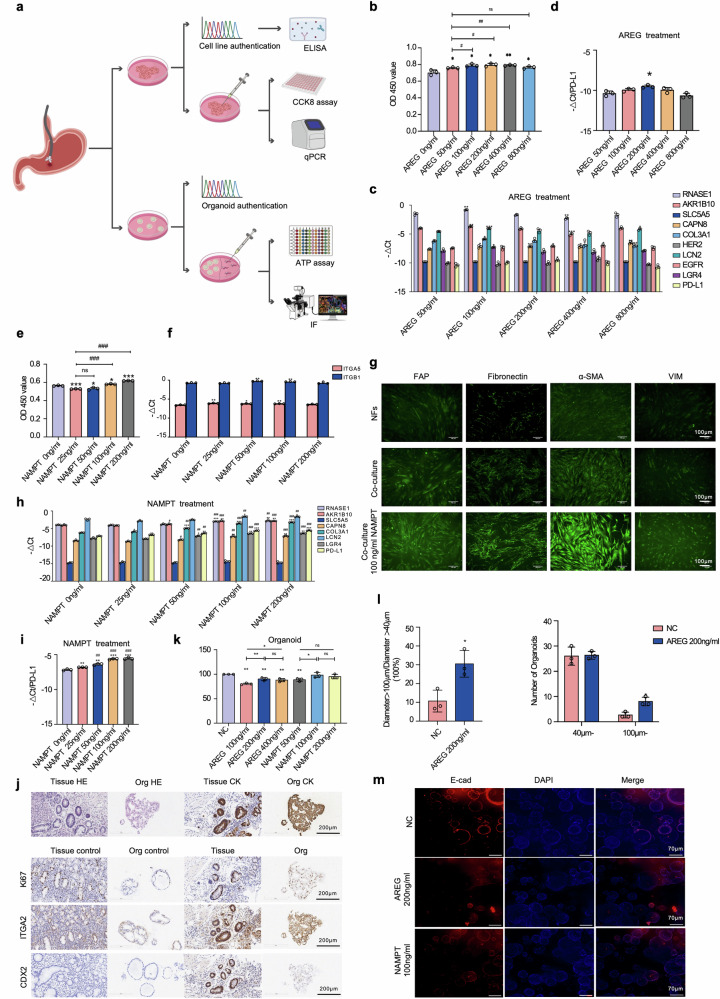
*AREG* and *NAMPT* enhance proliferation and gene modulation in precancerous gastric cell lines and organoids. **a** Precancerous cell lines (Pre-EGC) and organoids were successfully established from biopsies of EGC patients and treated with *AREG* and/or *NAMPT*. **b** CCK-8 assay demonstrating the impact of *AREG* on the viability of Pre-EGC after 72 h. **c** qPCR analysis of Pre-EGC treated with *AREG* for 72 h showing changes in the expression of “initiation-promoting” genes, *HER2*, *EGFR* and PD-L1. **d** qPCR analysis highlighting dose-dependent modulation of PD-L1 expression in Pre-EGC following *AREG* treatment (with 200 ng/ml the highest). **e** CCK-8 assay demonstrating the impact of *NAMPT* (0, 25, 50, 100, 200 ng/ml) on the viability of Pre-EGC after 72 h, with significant proliferation observed at 100 ng/ml. **f** Dose-dependent upregulated expressions of fibroblast surface receptors *ITGA5* and *ITGB1*, with the most significant increases observed at 25 ng/ml and 50 ng/ml, respectively. **g** Enhanced fluorescence intensity of CAF markers (*FAP*, fibronectin, α-SMA and VIM) observed in NFs co-cultured with Pre-EGC treated with 100 ng/ml *NAMPT* compared to normal fibroblasts (NFs) and untreated groups. **h** PCR analysis of Pre-EGC co-cultured with NFs treated with *NAMPT* for 72 h showing changes in the expression of “initiation-promoting” genes and PD-L1. **i** qPCR analysis highlighting dose-dependent modulation of PD-L1 expression in Pre-EGC following *NAMPT* treatment (significant with 100 ng/ml). **j** Validation and morphological characterization of precancerous gastric organoids. **k** ATP assay illustrating the proliferative effects of *AREG* on precancerous organoids after 5 days of treatment. **l** Organoids treated with 200 ng/ml *AREG* for 5 days were analyzed for size and gene expression. The left panel shows the number of organoids with diameters greater than 100 µm in the treatment group compared to controls. The right panel shows the proportion of organoids larger than 100 µm/40 µm in the treated group compared to controls. **m** Immunofluorescence analysis of E-cadherin in organoids treated with 200 ng/ml *AREG* or 100 ng/ml *NAMPT* for 5 days, revealing differential expression patterns. *p < 0.05, **p < 0.01, ***p < 0.01; #*p* < 0.05, ##*p* < 0.01, ###*p* < 0.001; ns no significance

To elucidate the roles of *AREG* and *NAMPT* in the malignant transformation of precancerous gastric epithelial cells, we successfully established primary cell lines derived from precancerous gastric lesions (Pre-EGC) and corresponding organoid models (Fig. 7a, Supplementary Fig. 5a, and Table 11). After successful polarization of macrophages into M1 and M2 phenotypes, *AREG* secretion was detected in both epithelial and macrophage supernatants, revealing that M1 macrophages secreted higher levels of *AREG* than M2 macrophages (Supplementary Fig. 5b–d, and Table 12). Proliferation assay of precancerous cells co-cultured with M1 macrophages showed a significant increase in cell viability across all *AREG*-treated Pre-EGC groups after 72 h, with the most pronounced effect observed in the 200 ng/ml treatment group (Fig. 7b and Supplementary Fig. 5e). qPCR analysis of the initial gene set and *HER2, EGFR*, and PD-L1 expression was performed in precancerous cells co-cultured with M1 macrophages. Genes such as *RNASE1*, *AKR1B10*, *CAPN8*, *COL3A1*, *LCN2 and LGR4* were significantly upregulated upon 100 ng/ml *AREG* treatment, while *EGFR* and PD-L1 upon 200 ng/ml *AREG* treatment, *HER2* upon 400 ng/ml *AREG* treatment (Fig. 7c, d).

*NAMPT* secretion was detected in the conditioned media of precancerous epithelial cell lines (Pre-EGC), indicating its active release from these cells (Supplementary Fig. 5f). To further investigate the functional role of *NAMPT*, we established a co-culture system comprising Pre-EGC and fibroblasts, we found: (1) dose-dependent enhancement of Pre-EGC proliferation under *NAMPT* treatment, with significant proliferation observed at 100 ng/ml (Fig. 7e); (2) dose-dependent upregulated expressions of fibroblast surface receptors *ITGA5* and *ITGB1* in response to *NAMPT* stimulation, with the most significant increases observed at 25 ng/ml and 50 ng/ml, respectively (Fig. 7f); (3) comparative analysis of normal fibroblasts (NFs), untreated NFs co-cultured with Pre-EGC, and those exposed to 100 ng/ml *NAMPT* revealing significantly enhanced fluorescence intensity of CAF markers (*FAP*, fibronectin, α-SMA and *VIM*) in the *NAMPT* treated group(Fig. 7g) which demonstrate that *NAMPT* drives fibroblast activation and facilitates malignant transformation, consistent with previously reported mechanisms^45^; (4) qPCR analysis of the initial gene set revealing that *COL3A1* and *PD-L1* were significantly upregulated following treatment with 25 ng/ml *NAMPT*, *AKR1B10*, *CAPN8*, and *LGR4* with 50 ng/ml *NAMPT* and *RNASE1*, *LCN2* expression with 100 ng/ml *NAMPT* treatment (Fig. 7h, i). Collectively, these results suggest that *NAMPT* not only enhances the proliferation of precancerous epithelial cells but also promotes fibroblast activation and malignant reprogramming, thereby contributing to early gastric carcinogenesis.

Organoid models recapitulated similar trends (Fig. 7j). After 5 days of treatment, an ATP assay showed a significant increase in organoid viability at 200 ng/ml and 400 ng/ml *AREG*, with 200 ng/ml showing the greatest effect, consistent with the cell line results. For *NAMPT*-treated organoids, the 100 ng/ml group demonstrated a significant increase in viability (Fig. 7k, Supplementary Fig. 5g, h). Additionally, the proportion of organoids exceeding 100 µm in diameter was significantly higher in the 200 ng/ml treatment group (Fig. 7l and Supplementary Fig. 5i, Table 13). Immunofluorescence staining revealed a significant decrease in *E-cadherin* expression in *NAMPT*-treated organoids, indicative of EMT, while *Fibronectin* expression remained unchanged (Fig. 7m and Supplementary Fig. 5j). Collectively, these findings underscore the pivotal roles of *AREG* and *NAMPT* in promoting cell proliferation, modulating gene expression, and driving phenotypic changes in the precancerous state, thereby contributing to malignant transformation.

### Evaluation of anti-*NAMPT*/*AREG* treatment in EGC prevention

**Fig. 8:**
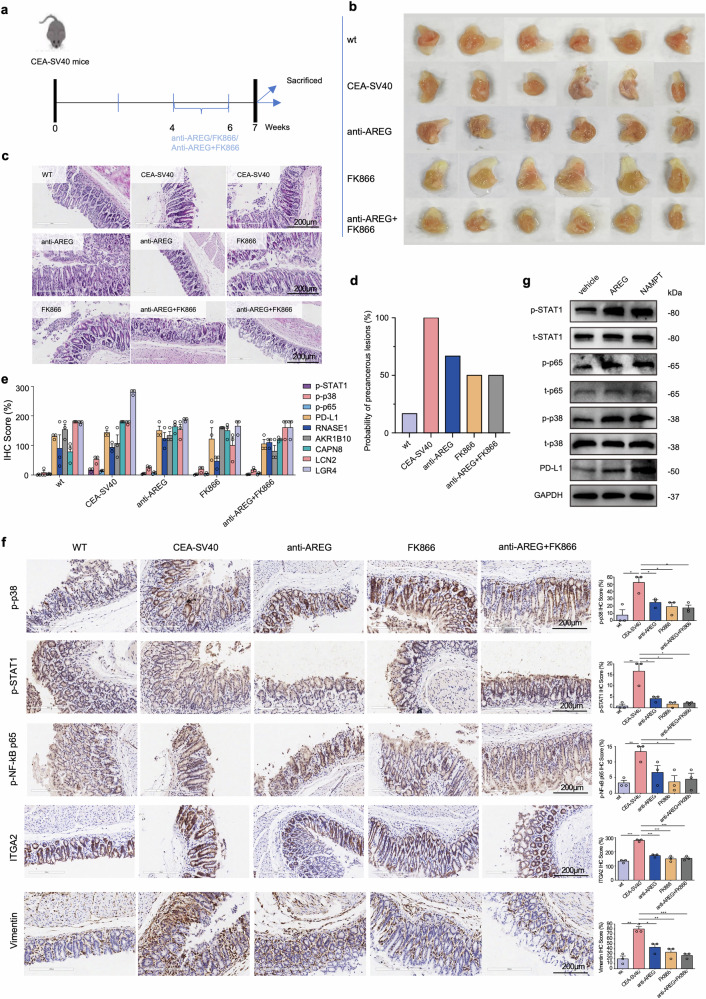
Therapeutic efficacy of anti-*AREG* and FK866 in the CEA-SV40 mouse model of EGC. **a** Schematic flowchart outlining the induction of EGC in CEA-SV40 mice and the oral administration protocol for anti-*AREG*, FK866, and the combination treatment (anti-*AREG* + FK866). **b** Representative images of mouse stomachs dissected longitudinally to expose the gastric mucosa for macroscopic examination. **c** Macroscopic examination of early lesions revealed no visible abnormalities in all groups. However, H&E staining identified distinct histological features: deep nuclear staining and polarity alterations were prominent in the untreated CEA-SV40 group, while the anti-*AREG* group exhibited mild dysplasia, and the FK866 and combined treatment groups showed the least dysplasia. 200 μm. **d** Probability of precancerous lesion formation (%) across groups. The untreated CEA-SV40 mice showed the highest incidence (~100%), while anti-*AREG* and FK866 treatments significantly reduced lesion probability. The combined treatment group demonstrated an approximately 50% reduction, comparable to individual treatments. **e** Immunohistochemical staining scores for “initiation-promoting” gene-set, NFκB, JAK/STAT, and MAPK pathways showed distinct expression patterns across groups. **f** Representative IHC staining images for PMC_2 markers (*ITGA2*), fibroblast marker (Vimentin) along with key pathway markers (pp38, p-STAT1 and pp65) revealed significant differences among groups. **g** Western blot analysis of PD-L1, STAT, p-STAT1, p38, p-p38, p65, and p-p65 after 72-h treatments in Pre-EGC, Pre-EGC + 200 ng/ml *AREG*, and Pre-EGC + fibroblasts groups treated with 100 ng/ml *NAMPT*/200 ng/ml *AREG*. *p < 0.05, **p < 0.01, ***p < 0.01

We utilized a transgenic CEA-SV40 mouse model^46^ to investigate the therapeutic effects of targeting *AREG* and *NAMPT* in EGC progression (Supplementary Fig. 6a) Treatments were administered as follows: mice were given drinking water containing 20 μg anti-AREG twice per week and/or 30 mg/kg FK866 twice daily for four consecutive days over 2 weeks, as described previously^47^ (Fig. 8a). At week 7, the mice were sacrificed, and both macroscopic (Fig. 8b) and histological evaluations (Fig. 8c) of their stomachs were performed. Pathological diagnoses revealed cancer occurrence rates of 16.7%, 100%, 66.7%, 50% and 50% in the wild-type (WT), CES-SV40 untreated, anti-*AREG*, FK866, and anti-*AREG* + FK866 groups, respectively (Fig. 8d). All treatment groups exhibited milder lesions compared to the untreated group, highlighting the potential of these interventions to delay EGC initiation.

Molecular analyses revealed differential expression of PD-L1, pSTAT, pp38, pp65, and the “initiation-promoting” genes, across groups (Fig. 8e and Supplementary Table 14). PD-L1, which was highly expressed in PMC_P and Pro_T tissues (Fig. 4g), was suppressed by *NAMPT* inhibition (FK866) (Supplementary Fig. 6b). Although total STAT1, p38, and p65 levels remained unchanged, pSTAT1 nuclear translocation and pp38 nuclear activity were markedly attenuated in all treated groups, and NF-κB activation (elevated nuclear pp65 expression) was significantly attenuated following FK866 treatment (Fig. 8f). “Initiation-promoting” genes exhibited differential susceptibility. *RNASE1* responded selectively to *NAMPT* inhibition, *CAPN8* to combined therapy, while *LGR4* was downregulated in all treatment groups (Supplementary Fig. 6c). PMC_2 marker (*ITGA2)* and fibroblast marker (*VIM*), were also markedly reduced in all treated groups, indicating lower PMC_2 and fibroblast infiltration, and their synergistic effect in disrupting the tumor-promoting microenvironment (Fig. 8f and Supplementary Table 14). We further assessed the expression of PD-L1, STAT, p-STAT1, p38, p-p38, p65, and p-p65 by western blot after 72-h treatments in Pre-EGC, Pre-EGC + 200 ng/ml *AREG*, and Pre-EGC+ fibroblasts treated with 100 ng/ml *NAMPT*. The WB results confirmed that both treatments led to the upregulation of these pathways, consistent with findings from the in vivo experiments (Fig. 8g). Collectively, these results indicate that anti-*AREG* and FK866 treatments reduce molecular drivers of EGC initiation and progression, underscoring their potential as therapeutic strategies for precancerous gastric lesions.

## Discussion

The 5-year survival rate for EGC exceeds 80–90%, but drops drastically to 20–30%^48^ in advanced stages, emphasizing the urgent need for early detection and intervention.^49,50^ Biomarkers and preventive strategies for EGC remain inadequately explored. While prior studies reconstructed carcinogenesis using fragmented biopsies across patients,^9,10,51^ this approach fundamentally obscures the spatial dynamics of malignant transformation. Our innovation lies in leveraging ESD specimens’ intrinsic spatial continuity—mapping molecular evolution from normal mucosa to IM to EGC within individual microenvironments. This strategy addresses the long-standing challenge of inter-patient heterogeneity that has hindered the field,^18,19^ enabling the construction of the first unified atlas of human gastric carcinogenesis.

Through AI-integrated spatial multi-omics, we identified the PMC_P niche—a novel carcinogenic tipping point^30^ spatially adjacent to tumors—driven by an inflammatory, stem-like PMC subpopulation. This niche orchestrates macrophage-epithelial (*AREG*$\longrightarrow$*EGFR/ERBB2*) and epithelial-fibroblast (*NAMPT*$\longrightarrow$*ITGA5/ITGB1*) crosstalk, collectively priming tumorigenic transformation. Elevated expression of both *AREG* and *NAMPT* in the precancerous stage was validated in 21 EGC patients. This robust clinical correlation further supports their potential role in the early phase of gastric carcinogenesis. Our multi-modal validation—from organoids to CEA-SV40 mice—confirms *NAMPT*/*AREG*’s causal role. We first established gastric precancerous cell lines and organoids, demonstrating that *NAMPT* and *AREG* significantly promote cellular proliferation in vitro. In preclinical models, inhibiting *AREG* and/or *NAMPT* suppressed NF-κB/JAK-STAT/MAPK activation and reversed PD-L1-mediated immune evasion, establishing their therapeutic relevance.

We first identified novel PMC cells (PMC_2) characterized by the over-expression of *IL-1β*, *PHLDA1*, and *ITGA2*. *IL-1β* polymorphisms have been associated with an increased risk of GC,^52^ while elevated *ITGA2/PHLDA1* levels are known to promote cell migration, invasion and resistance to apoptosis.^53^ Additionally, *PHLDA1*, a recognized stem cell marker in the human small and large intestine, functions downstream in the *ERBB2/EGFR* pathway,^54^ underscoring its potential role in the early stage of gastric carcinogenesis. We observed that while PMC_2 cells are present in both PMC_P and tumor regions, they exhibit spatiotemporal functional divergence. Compared to normal epithelium, PMC_2 cells are significantly enriched in the premalignant (PMC_P) region, alongside a transition from normal epithelial PMC_1 to PMC_2 cells. In PMC_P, they drive carcinogenesis via dual *NAMPT*$\longrightarrow$*ITGA5/ITGB1* (fibroblast) and *AREG*$\longrightarrow$*EGFR/ERBB2* (macrophage) axes, thereby activating stemness programs, rewiring oncogenic pathways, and establishing an immunosuppressive niche. In established tumors, PMC_2 may shift toward sustaining tumor progression (its region-specific functions warrant future investigation). Additionally, we identified a set of genes within the PMC_P region that promote EGC initiation, including *RNASE1*, a ribonuclease A family member implicated in RNA metabolism, angiogenesis, immune regulation, and cancer progression^55^; *LGR4*, a GPCR regulating cell proliferation, migration, and differentiation^34^; and *CAPN8*, which is predominantly expressed in the stomach, and appears to be involved in membrane trafficking in the gastric surface mucus cells.^56^ All three were functionally responsive to treatment in both in vitro and in vivo models. These findings position the PMC_P niche as a distinct precancerous tipping point, where transient signaling networks initiate malignant transformation. Notably, its molecular signatures offer a basis for non-invasive blood-based monitoring, paving the way for stratified clinical intervention.

Previous studies have established that *AREG* could be expressed by macrophage^57–60^ and plays a critical role in epithelial remodeling and tumorigenesis.^61–63^ Macrophage infiltration is a known hallmark of early tumor formation.^64^ Contrary to their classical anti-tumor role, *VEGFA* + M1 macrophages in the PMC_2 niche paradoxically drive early gastric tumorigenesis via *AREG*$\longrightarrow$*EGFR/ERBB2* signaling, fostering an immunosuppressive microenvironment that enables stromal remodeling and angiogenesis. While our data suggest macrophages are the predominant source of *AREG* in this context, *AREG* expression was also observed in PMC_2 and other cell types (Fig. 6c), indicating the possibility of complementary contributions to this signaling axis. This functional plasticity aligns with emerging evidence of M1 macrophages promoting carcinogenesis through ROS, cytokine-driven EMT, and immune evasion.^65–69^ Collectively, our findings implicate microenvironment-driven macrophage reprogramming in the early stages of cancer evolution. However, further lineage-specific studies are required to definitively determine the cellular origins of *AREG* and to clarify their respective contributions to tumor-initiating signaling.

We identified a *NAMPT*$\longrightarrow$*ITGA5/ITGB1* axis as a critical mediator of cell-cell interactions driving EGC, associated with enhanced NAD+ metabolism during the early stages of tumor development, which is consistent with previous research.^70^ It was revealed that *NNMT*-mediated nicotinamide metabolism was enriched in the gastric cell population that drives malignant progression. In our study, NAD+ metabolism was found to be enhanced during the early stages of GC development. Consistent with previous report,^71^ PD-L1, was absent in normal gastric mucosal epithelium, but showed prominent infiltrating in adjacent non-cancerous tissues from clinical ESD specimens. Notably, PD-L1 expression was induced by *NAMPT* activation, facilitating immune evasion via the *STAT1*-dependent INFγ signaling pathway. This aligns with clinical data linking *NAMPT* with poor outcomes.^72^ Critically, pharmacological inhibition of NAMPT using FK866 (currently in phase II trials for advanced malignancies^73–75^) attenuated EGC development in preclinical models, indicating its potential to intercept malignant transformation. Translation to human chemoprevention, however, requires clinical validation.

**Fig. 9:**
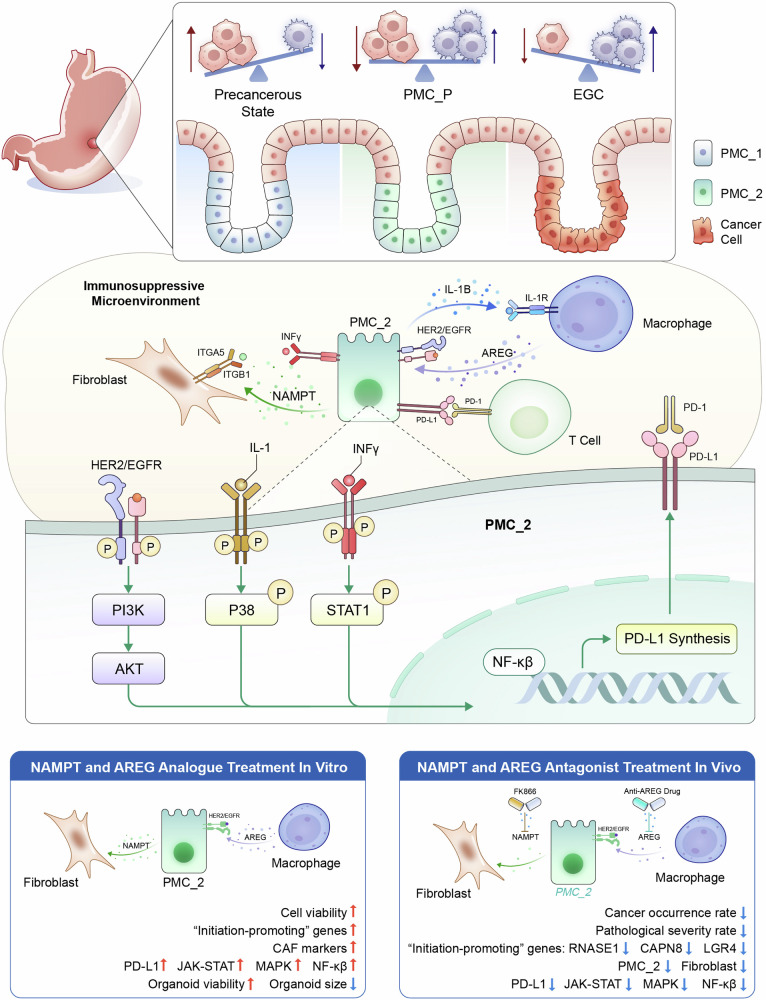
Graphical summary of molecular, cellular, and microenvironmental features defining the precancerous tipping point that drives EGC initiation. Using AI-integrated spatial multi-modal analysis of ESD specimens, we defined a precancerous tipping point niche (PMC_P) characterized by distinct molecular, cellular, and microenvironmental features. Within PMC_P, inflammatory pit mucous cells with stem-like properties (PMC_2) engage in pathological crosstalk with fibroblasts via *NAMPT* ⟶ *ITGA5*/*ITGB1* and with macrophages via *AREG* ⟶ *EGFR*/*ERBB2* signaling. These interactions collectively rewire oncogenic pathways, establish an immunosuppressive niche, and drive malignant transformation. The pivotal role of *NAMPT* and *AREG* signaling in EGC initiation was further validated through in vitro and in vivo models, including patient-derived cell lines, organoids and CEA-SV40 transgenic mice

In summary, our study defines the PMC_P niche as a signaling hub where *AREG/NAMPT* signaling duality drives metabolic-immune rewiring for EGC initiation (Fig. 9). Beyond mechanistic insights, our findings highlight tangible translational opportunities: (1) targeting this niche (e.g., anti-NAMPT therapy with FK866) can disrupt premalignant progression, reducing GC incidence in high-risk IM populations; (2) leveraging PMC_P-derived signatures for blood-based risk stratification, enabling precision surveillance endoscopy scheduling. While current study establishes foundational 2D transcriptomic profiles revealing PMC_P’s functional architecture, integrated 3D genome architecture analysis^76^ will resolve epigenetic drivers. Advanced spatial multi-omics/metabolomics^77^ will map post-translational modifications and metabolite gradients across the IM → PMC_P→tumor continuum. Implementation of SCENIC+ (single-cell regulatory network inference) will allow deeper exploration into internal gene modules and their regulatory dynamics.^78^ These efforts will support the next phase of our work to integrate the newly defined PMC_P and Pro_T domains with the classical GMC_P carcinogenic pathway, ultimately constructing a comprehensive landscape of EGC development.

## Materials and methods

### Human EGC sample collection and pathological annotation

This study was approved by the Ethics Committee of Zhongshan Hospital (B2020-393). Nine fresh-frozen tissue samples of EGC were obtained from Zhongshan Hospital, with approval from the Health Research Ethics Board of Fudan University. The clinical characteristics of the patients were summarized in Supplementary Table 1. Written informed consent was obtained from all patients before sample collection and data analysis. Tumor samples were collected during surgery, embedded in optimal cutting temperature compound, and stored at −80 °C until further processing. For this study, 10 µm thick cryosections were prepared from each tissue sample, stained with hematoxylin and eosin (H&E), and subsequently used for ST sequencing analysis. Additionally, adjacent tissue slices for four patients P4-P6, and P9 were selected for single-cell RNA sequencing. The pathologists reviewed the H&E-stained sections and annotated distinct tissue regions, including normal, IM, and tumor regions, to guide further data analysis.

### Spatial transcriptomic data processing and clustering analysis

Sequencing reads were aligned to the GRCh38 reference genome using the 10X Genomics Space Ranger pipeline (v1.1.0). Quality control was performed, and eight samples that met QC criteria were aggregated using the Space Ranger “aggr” function to normalize read depth across samples. This resulted in 20,063 spots with an average of 118,927 reads per spot.

We leveraged our previously developed AI model, stMVC,^24^ to elucidate spatial cellular niches and characterize their possible transition relationships. Specifically, stMVC employs a multi-view graph collaborative learning approach to integrate continuous disease progression features from histological images with localized cellular niches information from spatial locations. By further combining gene expression data and histological annotations, stMVC effectively learns spot embeddings that capture both spatial and temporal aspects of disease evolution, enabling a detailed characterization of disease dynamics over time. Spatial clusters were predicted from these embeddings using the Louvain algorithm. Various resolution parameters (0.4–1.6) were tested in the “Findclusters” function from Seurat^79^ for spatial clustering. Differentially expressed genes (DEGs) were identified in each cluster using the default Wilcoxon rank-sum test, and clusters were refined based on the expression pattern of DEGs.

To classify clusters from heterogeneous patients into unified groups consistent with pathological phenotypes, we manually reviewed the spatial distribution and marker gene expression levels of clusters, identifying six groups: normal, GMC_P, IM, PMC_P, Pro_T, and T. The Seurat package was then used to correct batch effects across patients, and DGEs were determined for each group compared to the others using the Wilcoxon rank-sum test.

### Receptor-ligand pairing analysis

To quantify the key CCC mechanisms in the PMC_P region, we performed LRP analysis on the ST data. For each patient, gene expression data and spatial clusters identified by stMVC in the epithelial tissue region were input into CellChat^80^ software to infer spatial cluster-level communications. To assess CCC patterns of LRPs at the spot level, we utilized our AI tool, stKeep,^44^ which employs heterogeneous graphs and contrastive learning to infer CCC for each spot/cell, ensuring that the learned CCC patterns are comparable across different clusters. This approach allowed us to quantify variations in CCC patterns of specific ligands and receptors associated with disease progression. Additionally, to elucidate how distinct cell types utilize the identified LRPs within the tumor microenvironment, we used CellChat to analyze scRNA-seq data. This analysis provided insights into CCC patterns between different cell types.

### Identification of cluster-specific highly expressed ligands

To identify the most expressed ligands in PMC_P cells, we proposed the Gini Index-based measure^81^ to estimate the relative expression level of each over-expressed ligand in PMC_P compared to five other groups: N, GMC_P, IM, Pro_T, and T. Specifically, we (i) identified over-expressed ligands in PMC_P using Wilcoxon rank-sum test; (ii) calculated the average expression level of each ligand in each group; (iii) calculated the Gini Index for each ligand using the “gini” function in the “reldist” package; and (iv) ranked the ligands based on their Gini index. The coefficient ranges from 0 to 1, where 0 indicates perfect equality and 1 represents perfect inequality. Higher coefficients indicate greater specificity of the ligand to the PMC_P group.

1 $${Gini}=\,\frac{{\sum }_{i=1}^{n}{\sum }_{j=1}^{n}\left|{x}_{i}-{x}_{j}\right|}{2{n}^{2}\bar{x}}$$

where ${x}_{i}$ is the mean expression level of a ligand in the ${ith}$ group, $n$ is the number of groups, and $\bar{x}$ is the mean expression level of a ligand across all groups.

### CNV estimation

To illustrate genomic diversity of nine epithelial cell subtypes, we used the InferCNV package (version 1.16.0) to infer large-scale chromosomal CNVs for each epithelial cell. This was achieved by sorting genes according to their chromosomal locations and utilizing moving averaged expression profiles, with a sliding window of 100 genes per chromosome.^82^ Genes with expression levels below 0.2 were excluded. To mitigate the influence of particular genes, we limited the relative expression values to the range [−3, 3], replacing values above 3 by 3 and those below −3 by −3. The resulting relative expression matrix was used for CNV estimation. Based on spatial mapping of nine epithelial subtypes, we treated PMC_1, GMC, and enteroendocrine cells as references and the remaining six subtypes as observations. Each cell was scored based on the extent of CNV signal, defined as the mean of squares of ${{CNV}}_{o}$ values across the genome, and for the correlation between the ${{CNV}}_{o}$ profile of each cell and the average ${{CNV}}_{o}$ of all cells from observation cells. After an initial analysis using the average ${{CNV}}_{o}$ of all cells as a reference, we obtained ${{CNV}}_{z}$ scores for each cell. We then redefined the CNV estimation using the average pattern of all reference cells, establishing a baseline reflecting the average ${{CNV}}_{z}$ of all reference cells, and the maximal (*BaseMax*) and minimal (*BaseMin*) baselines at each gene window. The final CNV estimation ${{CNV}}_{f}$ of cell $i$ at position $j$ was defined as:

2 $${{CNV}}_{f}\left(i,j\right)=\left\{\begin{array}{c}{{CNV}}_{z}\left(i,j\right)-{BaseMax}\left(j\right),{if}\,{{CNV}}_{z}\left(i,j\right)\, > \,{BaseMax}\left(j\right)+0.2\\ {{CNV}}_{z}\left(i,j\right)-{BaseMin}\left(j\right),{if}\,{{CNV}}_{z}\left(i,j\right)\, < \,{BaseMax}\left(j\right)-0.2\\ 0,{if}{{BaseMin}\left(j\right)-0.2\, < \,{CNV}}_{z}\left(i,j\right)\, < \,{BaseMax}\left(j\right)+0.2\end{array}\right.$$

Additionally, the same strategy was applied to analyze ST data by examining genomics variations across six epithelial groups: N, GMC_P, IM, PMC_P, and T, with N serving as the reference group.

### Pseudo-trajectory inference

We utilized the diffusion pseudotime (DPT)^28^ tool to analyze scRNA-seq data from nine epithelial cell subtypes. This method, based on diffusion maps, measures cell transitions using diffusion-like random walks. For each patient, MATLAB package DPT (version 1.0) was used to perform this analysis, where the input data was the corrected 30 PCs from Seurat package. The parameters of DPT was *transition matrix construction method* = “*nearest neighbors”*, *knn* = 20, *gstatmin* = 1.01, *nsig* = 10.

### Isolation and culture of primary cells and organoids

Fresh tissue samples were rinsed thoroughly with DMEM/F12 medium (Gibco) containing 100 U/ml penicillin and 100 μg/ml streptomycin (Sigma-Aldrich) to eliminate contaminants. Tissues were minced into small fragments and digested in 5 ml of enzymatic solution containing 2 mg/ml collagenase IV and 0.03 mg/ml DNase I (Sigma-Aldrich) at 37 °C for 30–60 min with gentle agitation. The digested cells were filtered through 100 μm strainers and centrifuged at 1500 rpm for 10 min. The single-cell suspension was cultured in a cell culture medium: DMEM/F12 medium (Gibco) plus 1 g/L Albumax (Gibco), 0.54 g/L Nicotinamide, 2 g/L NaHCO_3_, 5 mg/L Insulin, 5 mg/L Transferrin, 30 nM/L Selenium, 10^−6^ M hydrocortisone, 5 × 10^−5^ M beta-mercaptoethanol, 10^−9^ M Zinc Sulfate heptahydrate, 10 μg/ml high-density lipoprotein (Sigma-Aldrich), 2 mM L-glutamine, 10 μg/ml gentamicin, and 0.25 μg/mL amphotericin (Gibco). Cultures were maintained in a humidified incubator at 37 °C with 5% CO2.

For organoid cultures, freshly harvested tissues were rinsed using a tissue cleaning solution and subsequently minced into approximately 1 mm³ fragments using sterile scissors in a 6-cm petri dish. The fragments were transferred to a 1.5-ml Eppendorf tube and further digested with 1 ml of tissue digestion solution in a 37 °C water bath for 30 min. The digested mixture was filtered with a 100 μm cell screen to remove undigested residues. The cell pellet was resuspended in matrix gel at a ratio of 1:3 (pellet: matrix gel) and seeded in a 24-well plate (30–50 μl per well). Put the laid plate into the incubator at 37 °C for 10–15 min, and then add 1 ml organoid medium preheated at 37 °C for culture. Organoids were cultured until they reached a diameter of 150–200 μm, at which point they were passaged for expansion. Organoid media kit was provided by Shanghai Outdo Biotech Co., Ltd. The growth status of organoids can be observed with a microscope.

### Co-culture of primary epithelial cells and fibroblasts under *NAMPT* treatment

Primary gastric fibroblasts (CTCC-024-HUM) and primary epithelial cells (Pre-EGC) were sequentially digested, collected, and quantified. Fibroblasts were seeded into the upper chambers of 6-well Transwell inserts (0.4 μm pore size) at a density of 3 × 10⁴ cells per well, while primary epithelial cells were plated in the lower chambers at a density of 1 × 10⁶ cells per well. Co-culture was carried out using 3 mL of primary epithelial cell culture medium supplemented with varying concentrations of NAMPT (0, 25, 50, 100, or 200 ng/mL). Following a 72-h co-culture period, both epithelial cells and fibroblasts were harvested for further analysis.

### Co-culture of primary epithelial cells and macrophages

Primary epithelial cells were co-cultured with macrophages using a Transwell system (0.4 μm pore size). THP-1 monocytes were seeded into 6-well plates at a density of 6 × 10⁵ cells per well in 2 mL of macrophage culture medium. Once the cells reached 80–90% confluence, the culture medium was replaced with 2 mL of macrophage medium containing 100 ng/mL PMA to induce differentiation into M0 macrophages. Polarization was subsequently achieved by culturing M0 macrophages for 48 h in either: (1) M1-polarizing medium: 20 ng/mL IFN-γ + 100 ng/mL LPS; (2) M2-polarizing medium: 20 ng/mL IL-4 + 20 ng/mL IL-13. Following polarization, macrophages (THP-1, M0, M1, and M2 subtypes) were trypsinized, quantified, and seeded into Transwell upper chambers at a density of 4 × 10⁵ cells per well. Primary epithelial cells were similarly prepared and plated in the lower chambers at a density of 1 × 10⁶ cells per well with 3 mL of epithelial maintenance medium. After 72 h of co-culture, epithelial cells were collected for subsequent analyses.

### Co-culture of organoids and fibroblasts

Prior to seeding the cells, 4 mL of DPBS was added to the anti-evaporation chamber of the IBAC O2 chip (Daxiang Biotech, OE100811) and pre-warmed at 37 °C for 48 h. Organoids were enzymatically dissociated and suspended in a 1:2 mixture of culture medium and Datrigel (Daxiang Biotech, MG100101) at 4 °C. A 5 μL aliquot of this mixture was seeded into the main culture chamber. Fibroblast cells were enzymatically dissociated into single cells and suspended in a 4:1 mixture of culture medium (Daxiang Biotech, FC100101) and Datrigel (Daxiang Biotech, MG100101) at 4 °C. A 20 μL aliquot of this mixture was added to the auxiliary well. The IBAC O2 chip was incubated for 15 min, after which 60 μL of the corresponding culture medium was added to both the main culture well and the auxiliary well. The cells were stabilized and cultured for 48 h. The supernatants were then removed, and 350 μL of a 1:1 mixture of organoid culture medium and fibroblast culture medium was added to activate the interaction between the organoids and fibroblasts. After 24 h, the medium was refreshed with that containing the studied cytokines. The co-culture system was incubated for an additional 5 days.

### Passage breeding and germline construction of CEA-SV40 large T antigen mice

The H11-CEA/SV40 Tag mice were generated by Cyagen Company (Suzhou, China). Targeting vector was designed with (from 5′ to 3′) a CEA promoter (human carcinoembryonic antigen gene promoter), Kozak-SV40 large T antigen, a woodchuck hepatitis virus post-transcriptional regulatory element (WPRE; to enhance the mRNA transcript stability), and a polyA signal. The entire construct was inserted into H11 locus by CRISPR/Cas technology. Ribonucleoprotein (RNP) will be co-injected into fertilized eggs with the targeting vector for mice production.

Four F0 generation positive mice were obtained after identification. F0 generation mice with CEA-SV40 large T antigen exogenous gene were mated with non-transgenic mice to breed F1 generation positive mice, and each F0 generation mouse needed to be passed independently. After breeding, several F1 generation mice were obtained, and after Southern blot verification, 6 site-positive F1 mice were selected. After F1 generation mice were obtained, mice with the expression of the target gene were selected to be established for stable passage, and the passage situation and lineage were recorded at the same time. According to the experimental requirements, F1 and subsequent progeny were screened for self-crossbreeding to obtain homozygous mice.

### In vivo experiment

Four-week-old mice were divided into five groups (Wild Type: WT; Model Group: NC; Anti-AREG-Treatment Group; Anti-NAMPT-Treatment Group; Combined Medication Group). Anti-AREG (20 μg/mice), twice a week for three times. NAMPT inhibitor (FK866) (30 mg/kg), 8 times in 2 weeks, twice a day. On the second day after the last dose, whole stomach organs were removed from each group and photographed. The gastric tissues of fresh mice were fixed with 4% paraformaldehyde, dehydrated and embedded, then stained with HE and IHC.

### mIHC fluorescence signal quantification

Panoramic multispectral scanning of slides was performed by the Tissue-FAXS system (TissueFAXS Spectra, TissueGnostics GmbH, Vienna, Austria). Then, we imported the data into StrataQuest analysis software (Version 7.1.129, TissueGnostics GmbH, Vienna, Austria). The regions of interest were selected by pathologists. We used the spectral library for spectral splitting to obtain a single-channel fluorescence signal. The DAPI channel was used to identify the effective nucleus. Using the effective nucleus as the core, we set the distance radius according to the staining of each protein channel and found the protein fluorescent staining signal. Then, we set the threshold according to the staining situation of each channel, divided the positive cell population, and counted the positive cells. Triple-positive cells (A+B+C+) are defined as a cell population that simultaneously expresses three target proteins (e.g., Protein A, B, and C). These cells are identified using StrataQuest software (version 7.1.129), centered on valid cell nuclei, with thresholds established based on the fluorescence intensity of each protein channel to classify cells as positive (+) or negative (−). For triple-positive cells, Protein A-positive (A+) cells are first selected as the baseline. The fluorescence intensity of Protein B is plotted on the x-axis and that of Protein C on the y-axis to generate a scatter plot. Thresholds for Proteins B and C divide the scatter plot into four quadrants, with the upper-right quadrant (B+ and C+) corresponding to triple-positive cells (A+B+C+). StrataQuest software automatically counts the number of cells in each quadrant and calculates the proportion of triple-positive cells within the total cell population.

### Statistical analysis

The data in this study are shown as the mean ± standard error of the mean (SEM) of three independent experiments. Differences between groups were assessed by paired *t*-test or one-way analysis of variance (ANOVA) using the GraphPad Prism v8.0 software (Graphpad Software, La Jolla, CA, USA). Statistical significance was indicated as *P < 0.05, **P < 0.01, and ***P < 0.001.

## Supplementary information

Supplementary materials clean

All original and uncropped films of Western blots

Version 1 and 2 raw data of Figure 8f

All single-channel fluorescence images for Figures 6 and 8

## Data Availability

The scRNA-seq and spatial transcriptomics data supporting the findings of this study have been deposited into the OMIX database of the National Genomics Data Center, China National Center for Bioinformation (https://ngdc.cncb.ac.cn/omix/release/), under the accession number OMIX010346. The TCGA-derived datasets analyzed in this study were downloaded from cBioPortal (https://www.cbioportal.org). All additional data supporting the findings of this study are available from the corresponding author upon reasonable request.
