# Supplementary material for: Spatiotemporal multi-omics analysis uncovers NAD-dependent immunosuppressive niche triggering early gastric cancer
Source: Signal Transduct Target Ther. 2025 Sep 22;10:313. doi: 10.1038/s41392-025-02390-w (PMC12451012; doi:10.1038/s41392-025-02390-w)
Supplement: Supplementary file 1 — Supplementary materials clean [file 41392_2025_2390_MOESM1_ESM.docx]

**SIGTRANS-15882R3**

Supplementary Materials for

**Spatiotemporal multi-omics analysis uncovers NAD-dependent immunosuppressive niche triggering early gastric cancer**

**Spatial multimodal dissection of early gastric cancer**

Pingting Gao^1*^, Chunman Zuo^2*,#^, Wei Yuan^3^, Jiabin Cai^4^, Xiaoqiang Chai^4^, Ruijie Gong^4^, Jia Yu^1^, Lu Yao^1^, Wei Su^1^, Zuqiang Liu^1^, Shengli Lin^1^, Yun Wang^1^, Mingyan Cai^1^, Lili Ma^1^, Quanlin Li^1#^, Pinghong Zhou^1#^.

^1^Endoscopy Center and Endoscopy Research Institute, Zhongshan Hospital, Fudan University, Shanghai, China.

^2^School of Life Sciences, Sun Yat-sen University, Guangzhou, China

^3^Pathology Department, Zhongshan Hospital, Fudan University, Shanghai, China.

^4^ Department of Liver Surgery and Transplantation, Liver Cancer Institute, Zhongshan Hospital, Key Laboratory of Carcinogenesis and Cancer Invasion of Ministry of Education, Key Laboratory of Medical Epigenetics and Metabolism, Fudan University, Shanghai, China.

*Dr. Pingting Gao and Chunman Zuo are the co-first authors.

#Address correspondence to:

Pinghong Zhou, MD, PhD, Endoscopy Center and Endoscopy Research Institute, Zhongshan Hospital, Fudan University, 160 FengLin Road, Shanghai, 200032, China. e-mail: poemzhou@126.com.

Quanlin Li, MD, PhD, Endoscopy Center and Endoscopy Research Institute, Zhongshan Hospital, Fudan University, 160 FengLin Road, Shanghai, 200032, China. e-mail: li.quanlin@zs-hospital.sh.cn.

Chunman Zuo, School of Life Sciences, Sun Yat-sen University, 135 Xingang Xi Road, Guangzhou 510275, China. e-mail: zuochm@mail.sysu.edu.cn.

**This PDF file includes:**

Methods

Figures. S1 to S6

Tables S1 to S14

**Materials and methods**

**Spatial transcriptomic profiling**

Tissue optimization was performed to determine the optimal permeabilization time for EGC tissue in the downstream gene expression protocol. Spatial transcriptomics of EGC cryosections was conducted using the Visium Spatial Gene Expression Slide & Reagent Kit, 16 reactions (Catalog #1000184), according to the manufacturer's protocol (10x Genomics, Pleasanton, CA, USA). Briefly, 10 µm thick cryosections of OCT-embedded EGC samples were mounted onto Visium spatial slides. Enzymatic permeabilization was carried out for 24 minutes. mRNA captured on the slide via printed oligos was reverse transcribed to obtain cDNA. The cDNA was quantified using the Agilent Bioanalyzer High Sensitivity Kit (Catalog #5067-4626) on the Agilent Bioanalyzer 2100 instrument (Agilent Technologies, CA, USA). Following quantification, cDNA libraries were prepared and sequenced on an Illumina NovaSeq 6000 sequencer using the SP flowcell (200 cycles) at the Shanghai Biochip Centre.

**Single-cell RNA preparation and sequencing**

Tissues were dissected into 2-4 mm^3^ segments, and subjected to cellular lysis using a mixed enzyme solution. The resulting lysate was resuspended and filtered through a 70-μm cell strainer to remove debris. Cells were then collected by centrifugation at 300 × g for 7 minutes at 4 °C, followed by resuspension to a concentration of 700-1200 cells/μl. The entire heterogeneous cell population was analyzed without sorting or enrichment of specific subtypes. The cell suspension was loaded into the Chromium Single-Cell v2 3’ Chemistry Library preparation system, targeting the capture of 5,000-10,000 cells per sample. Library preparation was carried out following the manufacturer’s standard protocol, and sequencing was performed on the Illumina NextSeq 500 platform.

**scRNA-seq data processing and clustering**

The 10x Chromium single-cell RNA sequencing (scRNA-seq) data were processed using CellRanger (v3.1.0; 10x Genomics) for alignment, barcode assignment and unique molecular identifier (UMI) counting, with the genome reference set GRCh38-3.0.0. The filtered count matrices were converted to sparse matrices using the Seurat^1^ package (v4.0). Cells expressing fewer than 500 genes and with more than 10% mitochondrial reads were excluded from further analysis. The remaining data were log-normalized and scaled, with cell-cell variation due to UMI counts and percent mitochondrial reads regressed out.

To efficiently capture cellular diversity across samples, batch effects were corrected using the Harmony tool^2^. We then performed clustering analysis on the latent features learned from Harmony integration. Specifically, we identified 2,000 highly variable genes (HVGs) with the ‘FindVariableFeatures’ function in Seurat, extracted 20 principal components (PCs) from these HVGs, and applied Harmony to remove batch effects in these 20 PC embeddings. Cell clustering was conducted using the ‘FindClusters’ function with a resolution of 1. DEGs in each cluster were identified using the default Wilcoxon rank-sum test, and clusters were refined based on the canonical marker gene expression, ultimately identifying 12 different cell types.

For the analysis of epithelial cell and myeloid cell subtypes, canonical correlation analysis in Seurat was used to address batch effects. We selected 2,000 HVGs for anchoring using the ‘FindIntegrationAnchors’ function and applied 30 PCs for alignment with the ‘IntegrateData’ function. Cell clusters were predicted using the ‘FindClusters’ function at a resolution of 0.4. DEGs were identified in each cluster with the default Wilcoxon rank-sum test, and clusters were further defined based on canonical gene or DEG expression patterns.

**Gene set functional annotation**

We utilized the DAVID online website (https://david.ncifcrf.gov/) to perform gene enrichment analysis for the genes of interest, considering a term as enriched if the adjusted $p-value$ was less than 0.05. Additionally, to determine which gene programs are up- or down-regulated in PMC_2 cells versus PMC_1 cells (or PMC_P versus IM), we used gene set enrichment analysis (GSEA) with the “clusterProfiler” package^3^.

**ELISA**

Cells were seeded at a density of 1 × 10^4^ mL^−1^ in 25‑cm^2^ flasks, and 5ml medium was added for culture. When the cell density reaches 80-90%, remove the cell medium, and use the serum-free medium to clean three times. Then, the cells were incubated with serum-free medium for 48 h. The conditioned medium was harvested and centrifuged (1000 x g for 20 min, 4˚C). After centrifugation, the supernatant was collected for the subsequent enzyme‑linked immunosorbent assay (ELISA). The secretion levels of AREG and NAMPT were assessed using commercially available AREG and NAMPT ELISA kits (Meimian) according to the manufacturer's instructions.

**Real-time PCR**

Total RNA was isolated from cultured cells using TRIzol reagent (Sigma). RNA was reverse transcribed using PrimeScript™ RT Master Mix (TaKaRa). qPCR was performed using ChamQ Universal SYBR qPCR Master Mix (Vazyme). qPCR analysis was performed with SYBR Green Mixture in LightCycler® 480II (Roche). All results were normalized to β-actin expression. Primers for qPCR are listed as follows:

| **Gene** | **Forward primer sequence** | **Reverse primer sequence** |
| --- | --- | --- |
| *RNASE1* | CTGCCTCTTCCCTCTTCCTT | CCACCTCACAACCCACTCTT |
| *AKR1B10* | TGTCATCCCCAAGTCTGTG | GCCCTCCAGTTTCTGTTG |
| *CA2* | ACCCATCAGCGTCAGCA | TGTCCACCATCAGTTCTTCG |
| *CAPN8* | GTGGGTGGAGGTGGTCATT | GATTGGCTGGTGGTTTCTTC |
| *COL3A1* | TTGAAGGAGGATGTTCCCATCT | CAGACACATATTTGGCATGGTT |
| *LCN2* | ACAAAGACCCGCAAAAGATG | CTAAACAGGACGGAGGTGA |
| *LGR4* | AAATGGGAAGAGCAATCA | GTAGGAAATGGCAAACAA |
| *TNF-α* | TCAACCTCCTCTCTGCCATC | ATGTTCGTCCTCCTCACAGG |
| *IL-6* | GAAAGCAGCAAAGAGGCACT | GTTGGGTCAGGGGTGGTTAT |
| *iNOS (NOS2)* | CGTCCTGTCCCCTTTCTACT | CTGATTTTCCTGTCTCTGTCG |
| *CD206* | CGCCAGCAGTAGAACAAGC | ATGTCAATCCCAACCAAAACAG |
| *IL-10* | CGTGGAGCAGGTGAAGAAT | CTCAGTTTCGTATCTTCATTGTC |
| *HER2* | GCCTCCACTTCAACCACAG | GCCCAGACCATAGCACACTC |
| *EGFR* | GCTATGAGATGGAGGAAGACG | AGAGGAGGAGTATGTGTGAAGGA |
| *PD-L1* | CTACCTCTGGCACATCCTC | CACATCCATCATTCTCCCTT |
| *CCL22* | CGTCTGCTGCCGTGATT | GAGTCTGAGGTCCAGTAGAAGT |
| *ITGA5* | TGGTTTCCTTTCGGCTCTC | ATGGGGTGATTGGTGGTG |
| *ITGB1* | ACGGAGGAAGTAGAGGTTATT | CGCACTCTCCATTGTTACTG |

**Cell viability assay**

For cell viability assay, 4×10^3^ cells were cultured in a 96-well plate for 24 hrs and then treated with *AREG* (R&D Systems) for 72 hrs. The concentrations of addition of *AREG* are 0ng/ml, 50ng/ml, 100ng/ml, 200ng/ml, 400ng/ml and 800ng/ml. Survival cells were determined by Cell Counting Kit‐8 (Vazyme), and 10µL CCK-8 solution was added into each well (containing 100µL medium) with incubation at 37°C for 1h. Absorbance at 450 nm was measured using a microplate reader.

To quantitatively assess cell viability, the CellTiter Glo Luminescent Cell Viability Assay (Promega, G7570) was employed according to the manufacturer's protocol. Briefly, the IBAC O2 chip containing the organoids was equilibrated to room temperature for 30 min. The culture medium was gently removed from both the main culture well and the auxiliary well. 50 µL of CellTiter Glo reagent was added to each of the wells, followed by mixing the content for 2 mins on an orbital shaker to induce cell lysis. The IBAC O2 chip was incubated at room temperature for 10 min, and the luminescence was measured using a microplate reader (BioTek, MR-001) with an integration time of 0.1 seconds per well.

**gRNA design and synthesis**

In the http://crispor.tefor.net/ design gRNA, choose high-grade gRNA (matching forward strand of gene): GAACACTAGTGCACTTATCCTGG. The sequences of synthetic CrRNA (CRISPR RNA) (IDT Corporation) and tracrRNA (trans-activating crRNA) (Kingsley Biotechnology Corporation). The CrRNA will combine with tracrRNA to form a gRNA sequence.

**Western blotting**

Cell pellets were collected and resuspended in cold RIPA lysis buffer (Thermo Fisher Scientific) with protease inhibitor cocktail (Roche) and phosphatase inhibitor cocktail (MedChemExpress), incubated on ice for 30 minutes with a vortex every 5 minutes. The supernatants were collected by centrifuging at 4 °C, 13,000*g* for 15 min and quantified by BCA Assay (Thermo Fisher Scientific), then mixed with 5× SDS loading buffer and boiled for 5-10 min at 95 °C. The proteins were loaded on SDS-PAGE gels, electrophoresed, and then transferred to nitrocellulose membranes. Membranes were blocked with 5% BSA in TBST, and incubated with indicated primary antibodies in 5% BSA at 4 °C overnight. After being washed 3 times with TBST, Membranes were incubated with HRP-conjugated secondary antibody for 1 h at room temperature. Finally, a chemiluminescence substrate (ECL, Smart-Lifesciences) was added to the membranes and the western blot was resolved using a Luminescent imaging system (Tanon). The antibody information is as follows: anti-GAPDH (proteintech, 60004-1-Ig, 1:10000), anti-PD-L1 (proteintech, 17952-1-AP, 1:500), anti-p38 (proteintech, 66234-1-Ig, 1:2000), anti-phospho-p38 (Thr180/Tyr182) (proteintech, 28796-1-AP, 1:1000), anti-p65 ((STARTER, S0B0549, 1:1000), anti-phospho-p65 (Ser536) (Absin, abs146362, 1:500), anti-STAT1 (proteintech, 66545-1-Ig, 1:5000), anti-phospho-STAT1(Ser727) (proteintech, 28977-1-AP, 1:1000). The raw gel data are provided in Supplementary.

**Multiplexed immunohistochemistry (mIHC) and Immunofluorescence (IF) Assay**

4μm FFPE slides were stained for mIHC. Staining methods were validated by the EACRI IHC Core at the Providence Cancer Institute (Portland, OR) and are previously reported^4^. Briefly, slides were baked at 63℃ for 1h and then deparaffinized by a fully automatic dyeing machine (LEICAST5020, LEICA), and the antigen was retrieved. Then, commercial hydrogen peroxidase was used to remove the endogenous peroxidase for 10 mins. Next, the microarray was blocked for 10 min and incubated with one of the following antibodies to process mIHC staining for 1hr: anti-CK (PA125, dilution 1:10, Abcarta), anti-ITGA2 (ab181548, dilution 1:400, Abcam), anti-DCN (14667-1-AP, dilution 1:500, Proteintech), anti-ITGB1 (ab52971, dilution 1:200, Abcam), anti-ITGA5 (ab150361, dilution 1:200, Abcam), anti-NAMPT (ab236874, dilution 1:500, Abcam), anti-S100A8 (66853-1-Ig, dilution 1:300, Proteintech), anti-AREG (66433-1-Ig, dilution 1:300, Proteintech), anti-EGFR (PA192, ready-to-use, Abcarta), anti-ERBB2 (GT224502, ready-to-use, Gene Tech), anti-CD31 (GT232107, ready-to-use, Gene Tech), anti-CD68 (PA014, ready-to-use, Abcarta). After washing the slides with TBST, we incubated them with the secondary antibody (SM802, ready-to-use, DAKO) for 10 min. Next, opal dye (Opal 7-color Manual IHC Kit, NEL801001KT, PerkinElmer) diluent was added and incubated at room temperature for 10 mins. Microwave treatment was used to remove the antibody complex. Subsequent markers were counterstained, and the steps were repeated until all indicators in a panel were assessed. Slides were counterstained with DAPI for 5 mins and were enclosed with an antifade mounting medium.

Cells were cultured on coverslips (60-80% confluency) and washed three times with PBS (pH 7.4; Admas Life) using a horizontal shaker (NUOMI). Fixation was carried out with 4% paraformaldehyde (Biosharp) for 30 minutes at room temperature (25°C), followed by three PBS washes. Permeabilization and blocking were performed with 10% horse serum (Gibco) and 0.1% Triton X-100 (VWR) in PBS for 1 hour at room temperature. Cells were then incubated sequentially with primary antibodies: anti-FAP (1:200, Absin), anti-Fibronectin (1:500, MCE), anti-α-SMA (1:1000, STARTER), and anti-Vimentin (1:800, Proteintech). After PBS washes, FITC-conjugated secondary antibodies (1:700, STARTER) were applied for 1 hour at room temperature, in the dark. Nuclei were stained with DAPI (Beijing Puda Hemin Technology Co., Ltd.) and washed with PBS. Finally, coverslips were mounted with an antifade medium (HISTOVA), and images were captured using a fluorescence microscope (Olympus).

The organoid model was washed three times with DPBS. The cells were fixed with 1% paraformaldehyde (PFA) for 30 minutes and then rinsed three times with DPBS. The fixed cells were sequentially incubated with primary antibodies：anti-E-cadherin (60335-1-Ig, dilution 1:400, Proteintech), anti-Fibronectin (HY-P80493, dilution 1:200, MCE), washed with DPBS, incubated with secondary antibodies (ab150080, dilution 1:500, Abcam), and washed again with DPBS. The nuclei were stained with DAPI and washed with DPBS. The cytoskeleton was stained by Phalloidin (CA1620, Solarbio). Fluorescent images were captured using a high-content microscope (Molecular Devices, HT-001).

**Immunohistochemistry (IHC) for clinical ESD specimens and mouse model**

Clinical ESD specimens from 21 EGC patients and stomachs from CES-SC40 model were fixed with formalin, embedded with paraffin and immuno-stained. Briefly, the slides were deparaffinized in xylene, rehydrated through graded ethanol, quenched for endogenous peroxidase activity in 3% hydrogen peroxide, and processed for antigen retrieval by microwave heating in EDTA. The primary anti-LGR4 (20150-1-AP, dilution 1:2000, proteintech), anti-LCN2 (26991-1-AP, dilution 1:1000, proteintech), anti-CAPN8 (20971-1-AP, dilution 1:500, proteintech), anti-AKR1B10 (68327-1-Ig, dilution 1:10000, proteintech), anti-RNASE1 (abs118684, dilution 1:1000, Absin), anti-p-p65 (abs146362, dilution 1:2000, Absin), anti-p-STAT1(28977-1-AP, dilution 1:200, proteintech), anti-p-p38 (28796-1-AP, dilution 1:300, proteintech), anti-Vimentin (S0B2254, dilution 1:1000, Starter), anti-CD8 (S0B0034, dilution 1:1000, Starter), anti-PD-L1 (AD80167, ready-to-use, Abcarta), anti-ITGA2 (ab181548, dilution 1:2000, Abcam), anti-AREG (66433-1-1g, dilution 1:500, proteintech), were diluted in PBS containing 1% bovine serum albumin (BSA) and incubated at 4°C, followed by the incubation with secondary antibody (Dako, Carpinteria, CA, USA) for 60 min at room temperature and stained with (3,3′-diaminobenzidine, DAB; Dako) solution. The IHC results were determined by adding the scores for staining intensity (0; 1; 2; 3) and positive cell percentage (0-100%). All experiments were performed with triplicate biological replicates. Quantitative analysis was conducted by two independent investigators.


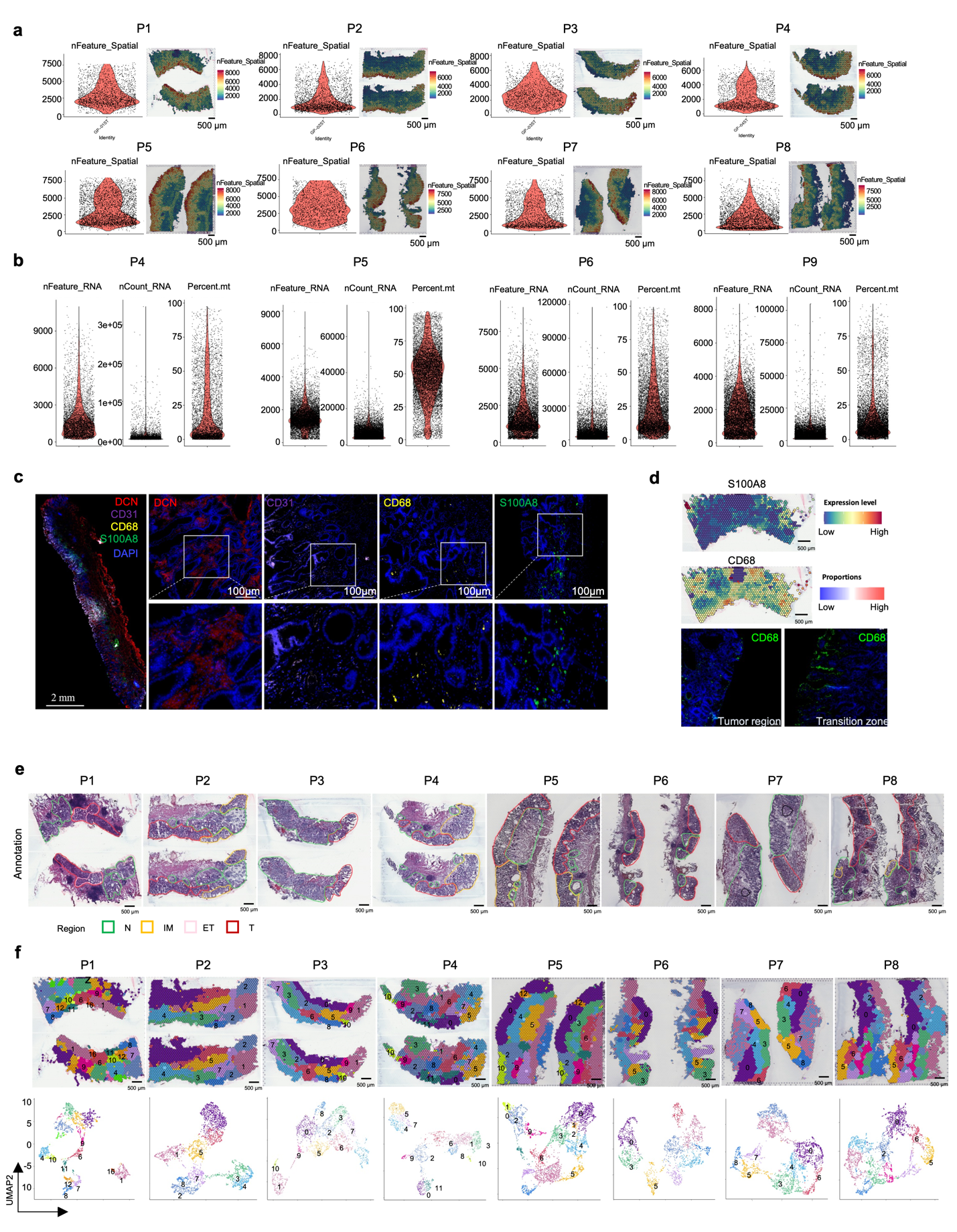


Figure. S1.

Processing of spatial transcriptomics and scRNA-seq data. a Violin plots and spatial distributions of detected feature counts in spatial transcriptomics data from eight patients. b Violin plots showing the distributions of nFeature_RNA, nCount_RNA, and mitochondria proportions in four scRNA-seq datasets. c Representative immunofluorescence images of marker genes of fibroblast (*DCN* and *CD31*) and macrophage (*CD68* and *S100A8*) in patient P2. d *CD68* showed poor specificity and technical variability compared to *S100A8* both spatially and under mIHC. e H&E image and annotations for eight patients, categorized into four types: normal (N), intestinal metaplasia (IM), early tumor (ET), and tumor (T). f Spatial distribution and UMAP visualization of clusters across eight spatial transcriptomics datasets, with each color indicating a distinct cluster.


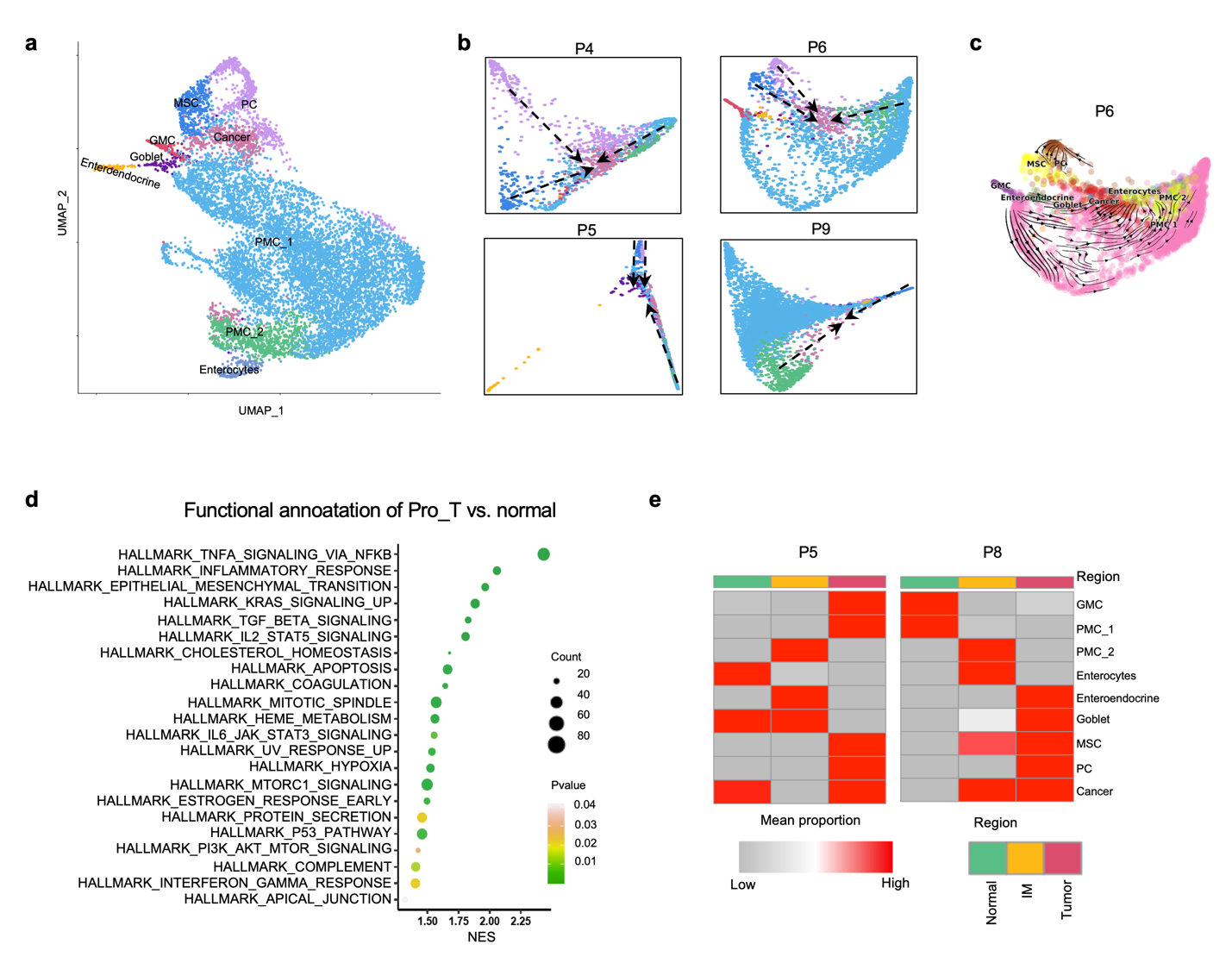


**Figure.S2.**

**Trajectory analysis of epithelial subtypes, GSEA for spatial Pro-T and epithelial subtypes spatial distribution.** **a** UMAP plot depicting nine epithelial cell subtypes. **b** Pseudotime trajectory analysis of epithelial cells derived from scRNA-seq data by patients. **c** RNA velocity analysis in P6. **d** GSEA analysis of genes upregulated in the Pro_T group compared with the normal group. **e** Heatmap showing the proportion of nine epithelial subtypes in spatial transcriptomics datasets from patients P5 and P8.

**
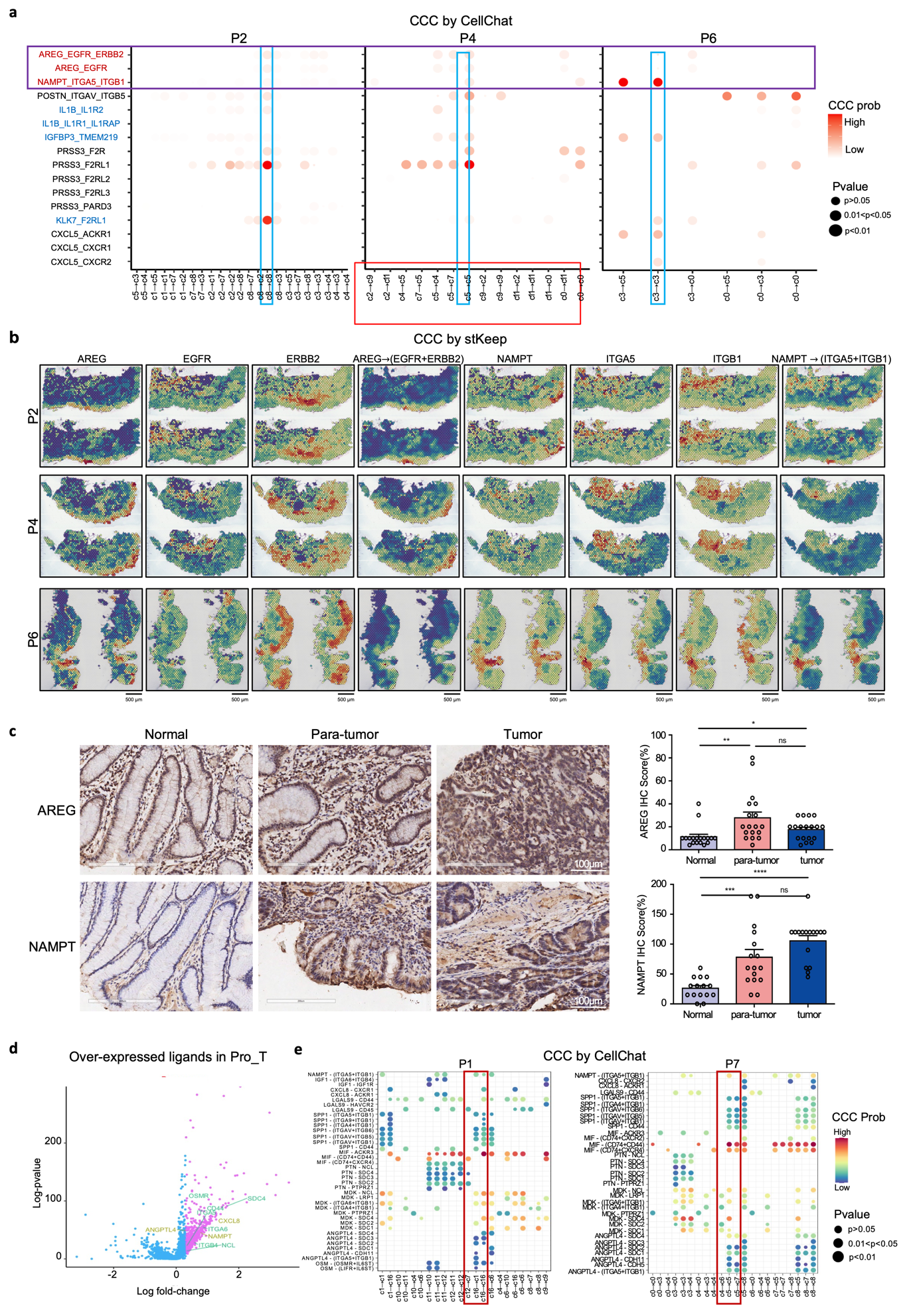
**

Figure. S3.

CCC and differential analysis for PMC_P and Pro_T groups. a Bubble heatmap displaying the mean CCC strength (calculated by CellChat) for interaction pairs including *AREG*, *IL1B*, *NAMPT*, *KLK7*, *IGFBP3*, *PRSS3*, *CXCL5*, and *POSTN* across different domains in three representative patients: P2, P4, and P6. b Spatial expression of the ligand *AREG*, receptors *EGFR* and *Erb-B2* receptor tyrosine kinase 2 (*ERBB2)*, and their corresponding CCC interactions in three representative patients: P2, P4, and P6. c AREG and NAMPT expression on 21 clinical ESD specimens. d Scatter plot showing the specificity (Gini index) and difference level ($\log\boldsymbol{P}\mathbf{-}\boldsymbol{value}$) of over-expressed ligand genes in the Pro_T group. e Bubble heatmap displaying CCC by CellChat for interaction pairs across different domains.


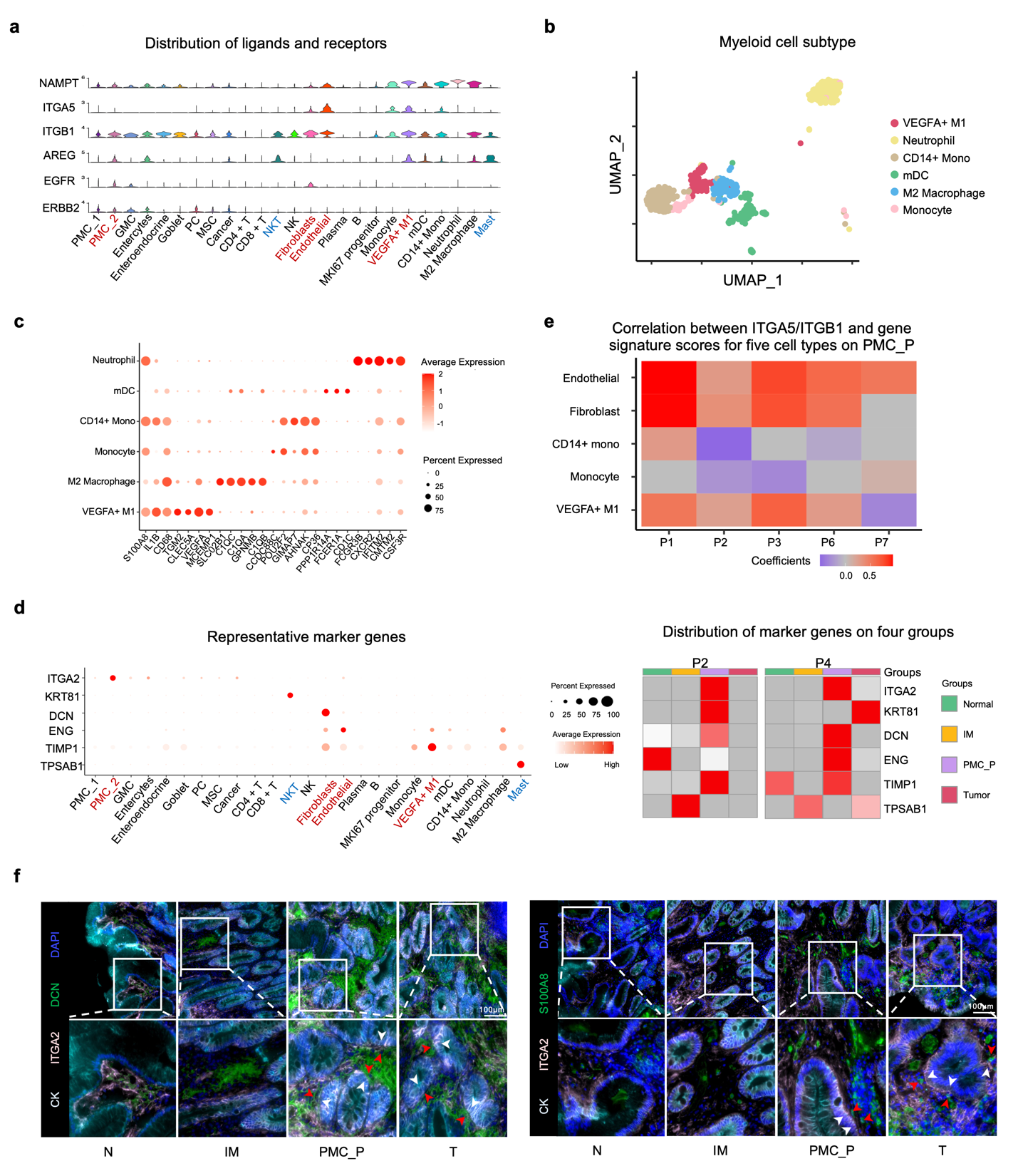


Figure. S4.

Spatially coordinated cellular crosstalk in the PMC_P precancerous niche drives malignant transformation. a Violin plot showing expression levels of ligands and receptors of LPRs, *AREG* $\boldsymbol{\longrightarrow}$ *EGFR*/*ERBB2* and *NAMPT* $\boldsymbol{\longrightarrow}$ *ITGA5*/*ITGB1*, in different cell types. b Umap plot illustrating 6 myeloid cell subtypes. c Dot plot showing the expression levels of marker genes in each subtype. Dot size and color indicate the percentage and mean expression level of each gene. d Representative marker distributions confirming interactions. e Spearman correlation between *ITGA5*/*ITGB1* and gene signature scores for five cell types, including endothelial (*CDH5*, *PECAM1*, *VWF*, and *ENG*), fibroblast (*CFD*, *DCN*, and *LUM*), *CD14*+ mono (*GIMAP7*, *AHNAK*, and *CD36*), monocyte (*CCDC88C*, and *POU2F2*), and *VEGFA*+ M1 (*TGM2*, *CLEC5A*, *VEGFA*, and *MCEMP1*). f Co-localization of *DCN*+ cells and *CK*+*ITGA2*+cells is evident, particularly within the PMC_P region where *DCN*+ cell infiltration is significantly concentrated, higher than in the tumor region. The boxed region highlights a magnified view, with white arrows indicating *CK*+*ITGA2*+cells, and red arrows pointing to *DCN*+ cells. Scale bar: 50 μm. *S100A8*+cells and *CK*+*ITGA2*+cells co-localization becomes more pronounced starting in the PMC_P region. Noticeable infiltration of myeloid cells was observed in the tumor region. The boxed area represents an enlarged display, with white arrows indicating *CK*+*ITGA2*+ cells and red arrows marking *S100A8*+ cells.


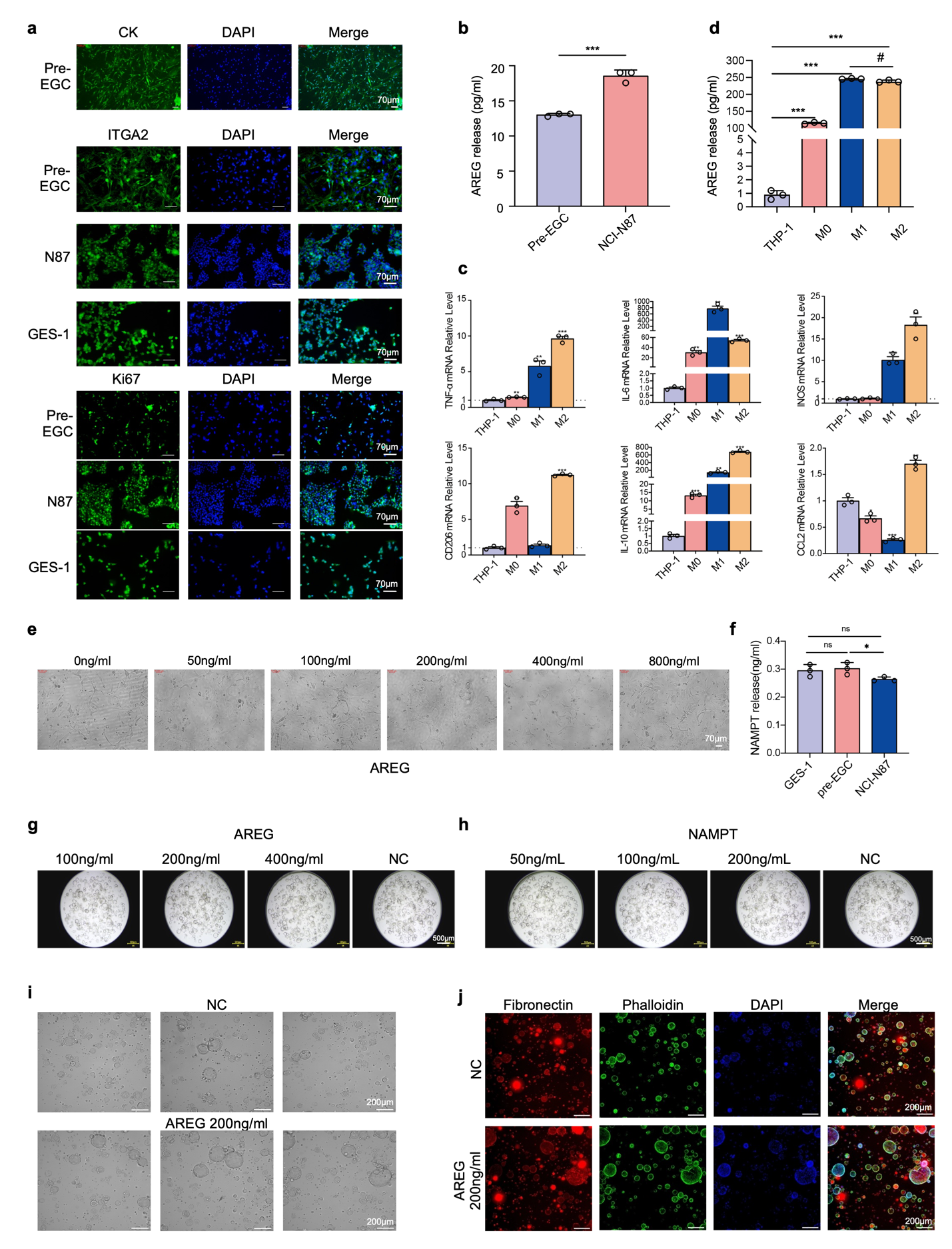


Figure. S5.

**AREG and NAMPT promote proliferation in gastric precancerous cells and organoids. a** Identification of pre-primary cells in gastric cancer. Scale bar: 70μm. **b** Release of AREG in Pre-EGC, NCI-N87 cell lines. **c** Successful polarization of macrophages into M1 and M2 phenotypes. **d** Release of AREG in THP-1, M0, M1, M2 cells. M1 macrophages secreted significantly higher levels of AREG compared to M2 macrophages. **e** Viability assay of pre-primary cells after AREG treatment. CCK 8 assay was performed after AREG treatment for 72 hours. Scale bar: 70μm. **f** Release of NAMPT in GES-1, Pre-EGC and NCI-N87 cell lines. **g&h** ATP analysis of gastric precancerous organoids following treatment with AREG or NAMPT in organoids. Scale bar: 200μm. **i** Cellular changes observed after the addition of 200ng/ml AREG to gastric precancerous organoids. **j** Immunofluorescence analysis of the gastric precancerous organoids after 200ng/ml AREG treatment for 5 days, showing no obvious difference in fibronectin expression. DAPI (blue), Fibronectin (red), Phalloidin (green). Scale bar: 200μm.


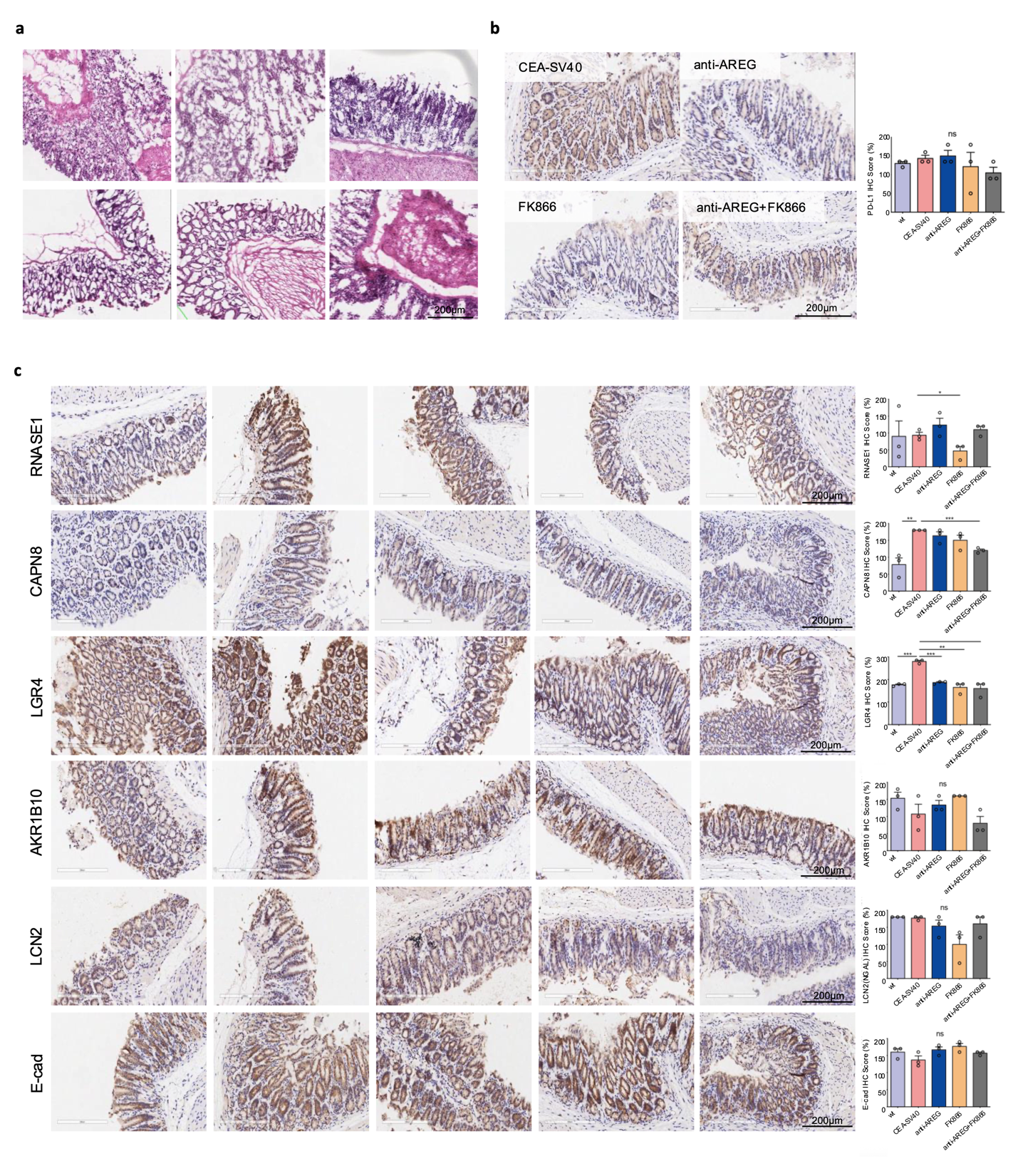


Figure. S6.

**Therapeutic effects of anti-AREG and FK866 on EGC initiation in CEA-SV40 mice. a** Representative H&E staining images showing dysplasia and neoplasia in gastric tissues of CEA-SV40 mice. Distinct morphological changes are observed across treatment groups: untreated, anti-AREG*-*treated, FK866-treated, and combined anti-AREG + FK866-treated. **b** Representative images depict PD-L1 expression in different groups, CEA-SV40 (control), anti-AREG-treated, FK866-treated, and combined anti-AREG + FK866-treated groups. Scale bar: 200μm. **c** Representative IHC staining images showing that "initiation-promoting" genes *RNASE1* was significantly affected by NAMPT inhibition, while *CAPN8* and *LGR4* were significantly decreased in all the treatment groups. Immunohistochemical staining results for *AKR1B10*, *LCN2*, and *E-cadherin* across groups, showing no notable differences in expression. Quantitative analysis confirms the absence of significant variation among treatment conditions. Scale bar: 200μm.

Table S1.

**Clinicopathological characteristics of 9 patients.**

| **Patient number** | **Age** | **Sex** | **Histopathological diagnosis** | **Site of origin** | **Lauren’s classification** | **Method** | **Tissue** |
| --- | --- | --- | --- | --- | --- | --- | --- |
|  |  |  |  |  |  |  |  |
| P1 | 64 | M | HGIN（Tub1）Well differentiated adenocarcinoma and NAG | Cardia | Intestinal | ST | ESD specimen |
| P2 | 56 | M | Tub2 Moderately differentiated adenocarcinoma and CAG | Angular incisure | Intestinal | ST | ESD specimen |
| P3 | 73 | M | Well differentiated adenocarcinoma and CAG | Angular incisure | Intestinal | ST | ESD specimen |
| P4 | 49 | F | Tub2 Moderately differentiated adenocarcinoma | Antrum | Intestinal | ST with scRNA-seq | ESD specimen |
| P5 | 71 | F | Tub1tub2 Well-Moderately differentiated adenocarcinoma | Antrum | Intestinal | ST with scRNA-seq | ESD specimen |
| P6 | 68 | F | Tub2 Moderately differentiated adenocarcinoma | Greater curvature | Intestinal | ST with scRNA-seq | ESD specimen |
| P7 | 63 | F | tub2 por1 tub1 Moderately-poorly-well differentiated adenocarcinoma | Antrum | Mixed | ST | ESD specimen |
| P8 | 74 | M | HGIN | Cardia | Intestinal | ST | ESD specimen |
| P9 | 63 | M | tub2 tub1 por2 Moderately-well-poorly differentiated adenocarcinoma | Antrum | Intestinal | ST with scRNA-seq | ESD specimen |

**Table S2.**

**Statistics for evaluating the quality of ST (10x genomics).**

|  | **Patient1** | **Patient2** | **Patient3** | **Patient4** | **Patient5** | **Patient6** | **Patient7** | **Patient8** | **Patient9** |
| --- | --- | --- | --- | --- | --- | --- | --- | --- | --- |
| Number of spots under tissue | 2,174 | 2,751 | 1,763 | 2,082 | 3,655 | 1,911 | 2,496 | 3,231 | 3887 |
| Mean Reads per spot | 96,403 | 85,048 | 166,875 | 131,106 | 82,282 | 160,410 | 131,719 | 97,572 | 82606 |
| Median genes per spot | 2,814 | 1,442 | 2,957 | 2,093 | 3,123 | 4,148 | 2,441 | 1,780 | 788 |
| Median UMI Counts per spot | 7,520 | 2,847 | 9,356 | 5,175 | 7,715 | 11,023 | 4,830 | 3,303 | 1147 |

Table S3.

**Statistics for evaluating the quality of scRNA-seq (10x genomics).**

|  | **GP-04SC** | **GP-05SC** | **GP-06SC** | **GP-09SC** |
| --- | --- | --- | --- | --- |
| Estimated Number of Cells | 5,567 | 11,549 | 9,456 | 10,432 |
| Fraction Reads in Cells | 80.30% | 74.40% | 80.20% | 84.80% |
| Mean Reads per Cell | 64,441 | 39,489 | 33,951 | 50,679 |
| Median Genes per Cell | 1,290 | 1,414 | 1,380 | 1,478 |
| Total Genes Detected | 23,940 | 22,612 | 23,696 | 23,081 |
| Median UMI Counts per Cell | 4,244 | 5,666 | 4,308 | 4,045 |
| Sequencing Saturation | 68.20% | 40.40% | 36.30% | 59.80% |
| Q30 Bases in UMI | 96.80% | 95.40% | 96.10% | 92.20% |
| Reads Mapped to Genome | 95.50% | 94.40% | 86.40% | 90.70% |
| Cell viability before library construction | 97.70% | 96% | 97% | 96% |

Table S4.
Statistics for cell numbers of each cluster in patients with scRNA-seq.

| **Cluster** | **GP-04SC** | **GP-05SC** | **GP-06SC** | **GP-09SC** |
| --- | --- | --- | --- | --- |
| MKI67+ progenitor | 141 | 13 | 52 | 33 |
| Plasma | 11 | 24 | 78 | 13 |
| Fibroblast | 99 | 0 | 15 | 6 |
| Endothelial | 34 | 1 | 39 | 3 |
| B | 649 | 56 | 630 | 741 |
| Epithelial | 1276 | 919 | 2721 | 5558 |
| Myeloid | 60 | 47 | 286 | 496 |
| Mast | 15 | 11 | 40 | 4 |
| NKT | 13 | 8 | 43 | 43 |
| NK | 13 | 11 | 66 | 171 |
| CD8+ T | 157 | 54 | 534 | 435 |
| CD4+ T | 292 | 130 | 440 | 358 |

**Table S5.**

**Number and proportion of cells in each cell type.**

| **Cluster** | **Cell number** | **Proportions for a cell type** |
| --- | --- | --- |
| MKI67+ progenitor | 239 | 0.0142 |
| Plasma | 126 | 0.0074 |
| Fibroblast | 120 | 0.0071 |
| Endothelial | 77 | 0.0046 |
| B | 2076 | 0.1233 |
| Epithelial | 10474 | 0.622 |
| Myeloid | 889 | 0.0528 |
| Mast | 70 | 0.0042 |
| NKT | 107 | 0.0064 |
| NK | 261 | 0.0155 |
| CD8+ T | 1180 | 0.07 |
| CD4+ T | 1220 | 0.07245 |

**Table S6.**

**Cell clusters and marker genes.**

| **Cluster** | **All marker genes** |
| --- | --- |
| MKI67+ progenitor | *AURKB* |
| MKI67+ progenitor | *CCNA2* |
| MKI67+ progenitor | *CCNB2* |
| NKT | *CD160* |
| NKT | *KRT81* |
| NKT | *KIR2DL4* |
| Plasma | *JCHAIN* |
| Plasma | *MZB1* |
| Plasma | *DERL3* |
| Fibroblast | *CFD* |
| Fibroblast | *DCN* |
| Fibroblast | *LUM* |
| Mast | *TPSB2* |
| Mast | *MS4A2* |
| Mast | *TPSAB1* |
| Mast | *KIT* |
| Endothelial | *CDH5* |
| Endothelial | *PECAM1* |
| Endothelial | *VWF* |
| Endothelial | *ENG* |
| Myeloid | *S100A8* |
| Myeloid | *S100A9* |
| Myeloid | *IL1B* |
| Myeloid | *CD68* |
| NK | *GNLY* |
| NK | *CST7* |
| NK | *CCL4* |
| NK | *GZMB* |
| NK | *KLRD1* |
| CD4+ T | *IL7R* |
| CD4+ T | *CCR7* |
| CD4+ T | *CD4* |
| CD8+ T | *CD8A* |
| CD8+ T | *CD8B* |
| CD8+ T | *TRAC* |
| B | *CD79A* |
| B | *MS4A1* |
| B | *IGHM* |
| Epithelial | *EPCAM* |
| Epithelial | *KRT18* |
| Epithelial | *KRT19* |

**Table S7. IHC results of Human ITGA2**

| **SliceNumber** | **Normal** | | | **Para-tumor** | | | **Tumor** | | |
| --- | --- | --- | --- | --- | --- | --- | --- | --- | --- |
|  | **intensity** | **positive rate** | **IHC Score （%）** | **intensity** | **positive rate** | **IHC Score （%）** | **intensity** | **positive rate** | **IHC Score （%）** |
| 24G124077-002 | 2 | 80% | 160 | 3 | 95% | 285 | 2 | 90% | 180 |
| 24G125973-003 | 2 | 15% | 30 | 2 | 90% | 180 | 2 | 90% | 180 |
| 24G121169-003 | 2 | 30% | 60 | 3 | 75% | 225 | 2 | 90% | 180 |
| 24G123213-002 | 3 | 10% | 30 | 3 | 90% | 270 | 3 | 80% | 240 |
| 24G124076-003 | 2 | 20% | 40 | 2 | 90% | 180 | 2 | 90% | 180 |
| 24G121167-007 | 3 | 10% | 30 | 3 | 90% | 270 | 2 | 80% | 160 |
| 24G114683-002 | 2 | 90% | 180 | 3 | 95% | 285 | 2 | 80% | 160 |
| 24G120637-002 | 2 | 60% | 120 | 3 | 95% | 285 | 2 | 90% | 180 |
| 24G124073-002 | 2 | 70% | 140 | 3 | 90% | 270 | 3 | 60% | 180 |
| 24G124074-003 | 3 | 25% | 75 | 2 | 90% | 180 | 2 | 90% | 180 |
| 24G125761-002 | 2 | 45% | 90 | 2 | 90% | 180 | 2 | 90% | 180 |
| 24G121171-002 | 2 | 45% | 90 | 2 | 90% | 180 | 3 | 65% | 195 |
| 24G115204-002 | 2 | 80% | 160 | 3 | 90% | 270 | 2 | 90% | 180 |
| 24G114865-003 | 3 | 20% | 60 | 3 | 95% | 285 | 2 | 80% | 160 |
| 24G120284-003 | 2 | 80% | 160 | 3 | 95% | 285 | 2 | 95% | 190 |
| 24G110633-003 | 2 | 45% | 90 | 3 | 90% | 270 | 2 | 50% | 100 |
| Mean of IHC Score |  |  | 94.7 |  |  | 243.8 |  |  | 176.6 |
| SEM of IHC Score |  |  | 13.1 |  |  | 11.7 |  |  | 6.9 |

**Table S8. IHC results of Human PD-L1**

| SliceNumber | Normal | | | Para-tumor | | | Tumor | | |
| --- | --- | --- | --- | --- | --- | --- | --- | --- | --- |
|  | intensity | positive rate | IHC Score （%） | intensity | positive rate | IHC Score （%） | intensity | positive rate | IHC Score （%） |
| 24G111264-002 | 1.5 | 2% | 3 | 0 | 0% | 0 | 0 | 0% | 0 |
| 24G120284-003 | 2 | 1% | 2 | 0 | 0% | 0 | 0 | 0% | 0 |
| 24G124076-003 | 0 | 0% | 0 | 2 | 2% | 4 | 0 | 0% | 0 |
| 24G125974-003 | 0 | 0% | 0 | 0 | 0% | 0 | 0 | 0% | 0 |
| 24G125402-002 | 0 | 0% | 0 | 0 | 0% | 0 | 0 | 0% | 0 |
| 24G125402-003 | 0 | 0% | 0 | 0 | 0% | 0 | 0 | 0% | 0 |
| 24G125973-003 | 0 | 0% | 0 | 0 | 0% | 0 | 0 | 0% | 0 |
| 24G125763-002 | 0 | 0% | 0 | 0 | 0% | 0 | 0 | 0% | 0 |
| 24G125073-002 | 0 | 0% | 0 | 0 | 0% | 0 | 2 | 10% | 20 |
| 24G121171-002 | 0 | 0% | 0 | 0 | 0% | 0 | 0 | 0% | 0 |
| 24G124077-002 | 0 | 0% | 0 | 2 | 2% | 4 | 0 | 0% | 0 |
| 24G124080-002 | 0 | 0% | 0 | 0 | 0% | 0 | 0 | 0% | 0 |
| 24G110633-003 | 0 | 0% | 0 | 0 | 0% | 0 | 0 | 0% | 0 |
| 24G115202-002 | 0 | 0% | 0 | 0 | 0% | 0 | 0 | 0% | 0 |
| 24G120637-002 | 0 | 0% | 0 | 2 | 10% | 20 | 0 | 0% | 0 |
| 24G123213-002 | 0 | 0% | 0 | 0 | 0% | 0 | 2 | 1% | 2 |
| 24G121167-007 | 0 | 0% | 0 | 0 | 0% | 0 | 0 | 0% | 0 |
| 24G114865-003 | 0 | 0% | 0 | 0 | 0% | 0 | 0 | 0% | 0 |
| 24G115204-002 | 0 | 0% | 0 | 0 | 0% | 0 | 0 | 0% | 0 |
| 24G114683-002 | 0 | 0% | 0 | 2 | 1% | 2 | 0 | 0% | 0 |
| 24G121169-003 | 0 | 0% | 0 | 0 | 0% | 0 | 0 | 0% | 0 |
| 24G124074-003 | 0 | 0% | 0 | 0 | 0% | 0 | 2 | 1% | 2 |
| Mean of IHC Score |  |  | 0.2 |  |  | 1.4 |  |  | 1.1 |
| SEM of IHC Score |  |  | 0.2 |  |  | 0.9 |  |  | 0.9 |

**Table S9. IHC results of Human AREG**

| **SliceNumber** | **Normal** | | | **Para-tumor** | | | **Tumor** | | |
| --- | --- | --- | --- | --- | --- | --- | --- | --- | --- |
|  | **intensity** | **positive rate** | **IHC Score （%）** | **intensity** | **positive rate** | **IHC Score （%）** | **intensity** | **positive rate** | **IHC Score （%）** |
| 24G110633-003 | 2 | 3% | 6 | 3 | 10% | 30 | 2 | 10% | 20 |
| 24G111264-002 | 2 | 5% | 10 | 3 | 5% | 15 | 2 | 5% | 10 |
| 24G114683-002 | 2 | 5% | 10 | 3 | 5% | 15 | 2 | 3% | 6 |
| 24G114865-003 | 2 | 5% | 10 | 3 | 5% | 15 | 2 | 2% | 4 |
| 24G115204-002 | 2 | 20% | 40 | 2 | 40% | 80 | 2 | 15% | 30 |
| 24G120637-002 | 2 | 5% | 10 | 2 | 20% | 40 | 2 | 10% | 20 |
| 24G121171-002 | 2 | 5% | 10 | 3 | 15% | 45 | 2 | 5% | 10 |
| 24G121167-007 | 2 | 5% | 10 | 2 | 5% | 10 | 2 | 15% | 30 |
| 24G121169-003 | 2 | 5% | 10 | 2 | 15% | 30 | 2 | 15% | 30 |
| 24G123213-002 | 2 | 5% | 10 | 3 | 25% | 75 | 2 | 15% | 30 |
| 24G124077-002 | 2 | 15% | 30 | 2 | 20% | 40 | 2 | 5% | 10 |
| 24G124076-003 | 2 | 3% | 6 | 2 | 10% | 20 | 2 | 10% | 20 |
| 24G124080-002 | 2 | 3% | 6 | 2 | 10% | 20 | 2 | 10% | 20 |
| 24G124073-002 | 2 | 2% | 4 | 2 | 5% | 10 | 2 | 10% | 20 |
| 24G124074-003 | 2 | 5% | 10 | 2 | 10% | 20 | 2 | 10% | 20 |
| 24G125761-002 | 2 | 3% | 6 | 2 | 10% | 20 | 2 | 5% | 10 |
| 24G125974-003 | 2 | 2% | 4 | 2 | 2% | 4 | 2 | 3% | 6 |
| 24G125973-003 | 2 | 5% | 10 | 2 | 5% | 10 | 2 | 10% | 20 |
| Mean of IHC Score |  |  | 11.2 |  |  | 27.7 |  |  | 17.6 |
| SEM of IHC Score |  |  | 2.1 |  |  | 5.1 |  |  | 2.1 |

**Table S10. IHC results of Human NAMPT**

| **SliceNumber** | **Normal** | | | **Para-tumor** | | | **Tumor** | | |
| --- | --- | --- | --- | --- | --- | --- | --- | --- | --- |
|  | **intensity** | **positive rate** | **IHC Score （%）** | **intensity** | **positive rate** | **IHC Score （%）** | **intensity** | **positive rate** | **IHC Score （%）** |
| 24G124074-003 | 1.5 | 30% | 45 | 2 | 90% | 180 | 1.5 | 80% | 120 |
| 24G110633-003 | 1.5 | 20% | 30 | 2 | 40% | 80 | 2 | 60% | 120 |
| 24G114683-002 | 1.5 | 10% | 15 | 2 | 20% | 40 | 1.5 | 80% | 120 |
| 24G121167-007 | — | — | — | 2 | 30% | 60 | 1.5 | 80% | 120 |
| 24G121171-002 | 1.5 | 20% | 30 | — | — | — | — | — | — |
| 24G115204-002 | 1.5 | 30% | 45 | 2 | 25% | 50 | 1.5 | 80% | 120 |
| 24G111264-002 | 1.5 | 10% | 15 | 2 | 50% | 100 | 1.5 | 80% | 120 |
| 24G124076-003 | — | — | — | 2 | 40% | 80 | 1.5 | 80% | 120 |
| 24G115202-002 | 1.5 | 10% | 15 | 2 | 20% | 40 | 1.5 | 30% | 45 |
| 24G124077-002 | 1.5 | 10% | 15 | 2 | 30% | 60 | 1.5 | 80% | 120 |
| 24G124080-002 | 1.5 | 10% | 15 | 2 | 20% | 40 | 1.5 | 80% | 120 |
| 24G125974-003 | 1.5 | 20% | 30 | — | — | — | — | — | — |
| 24G125761-002 | 0 | 0% | 0 | — | — | — | — | — | — |
| 24G120637-002 | 1.5 | 60%% |  | 2 | 60% | 120 | 1 | 60% | 60 |
| 24G123213-002 | 1.5 | 30% | 45 | 1.5 | 10% | 15 | 1.5 | 35% | 52.5 |
| 24G125973-003 | 2 | 15% | 30 | 2 | 90% | 180 | 2 | 90% | 180 |
| 24G114865-003 | 0 | 0% | 0 | 1.5 | 10% | 15 | 1.5 | 40% | 60 |
| 24G121169-003 | 1.5 | 40% | 60 | 2 | 65% | 130 | 1.5 | 60% | 90 |
| Mean of IHC Score |  |  | 26.0 |  |  | 79.3 |  |  | 104.5 |
| SEM of IHC Score |  |  | 4.5 |  |  | 13.7 |  |  | 9.3 |

**Table S11.**

| **Standard curve** | | **Standard curve** | |
| --- | --- | --- | --- |
| **OD450** | **AERG concentration pg/ml** | **OD450** | **NAMPT concentration ng/ml** |
| 0.052 | 0 | 0.047 | 0 |
| 0.16 | 31.25 | 0.065 | 0.3125 |
| 0.28 | 62.5 | 0.116 | 0.625 |
| 0.372 | 125 | 0.195 | 1.25 |
| 0.719 | 250 | 0.362 | 2.5 |
| 1.367 | 500 | 0.764 | 5 |
| 2.798632074 | 1000 | 1.41 | 10 |

|  | **OD value** | | | **AREG concentration pg/ml** | | |  | | | |
| --- | --- | --- | --- | --- | --- | --- | --- | --- | --- | --- |
|  | **OD-1** | **OD-2** | **OD-3** | **concentration-1** | **concentration-2** | **concentration-3** | **T-Test analysis** | | | |
| GES-1 | 2.765 | 2.615 | 2.511 | 996.886 | 941.661 | 903.371 | GES-1 | vs | pre-EGC | 4.25E-06 |
| pre-EGC | 0.093 | 0.093 | 0.092 | 13.136 | 13.136 | 12.768 | GES-1 | vs | NCI-N87 | 4.35E-06 |
| NCI-N87 | 0.109 | 0.105 | 0.109 | 19.027 | 17.554 | 19.027 | pre-EGC | vs | NCI-N87 | 4.00E-04 |

|  | **OD value** | | | **NAMPT concentration ng/ml** | | |  | | | |
| --- | --- | --- | --- | --- | --- | --- | --- | --- | --- | --- |
|  | **OD-1** | **OD-2** | **OD-3** | **concentration-1** | **concentration-2** | **concentration-3** | **T-Test analysis** | | | |
| GES-1 | 0.069 | 0.072 | 0.075 | 0.273 | 0.295 | 0.316 | GES-1 | vs | pre-EGC | 7.04E-01 |
| pre-EGC | 0.073 | 0.07 | 0.076 | 0.302 | 0.281 | 0.324 | GES-1 | vs | NCI-N87 | 7.98E-02 |
| NCI-N87 | 0.067 | 0.067 | 0.069 | 0.259 | 0.259 | 0.273 | pre-EGC | vs | NCI-N87 | 4.53E-02 |

**Expression levels of AREG and NAMPT in GES-1, pre-EGC, NCI-N87 cell supernatants (Elisa raw data).**

**Table S12.**

| **Standard curve** | |
| --- | --- |
| **OD450** | **AERG concentration pg/ml** |
| 0.038 | 0 |
| 0.206 | 31.25 |
| 0.329 | 62.5 |
| 0.639 | 125 |
| 0.951 | 250 |
| 1.464 | 500 |
| 3.262 | 1000 |

**Expression levels of AREG in THP-1, M0, M1, M2 cell supernatants (Elisa raw data).**

|  | **OD value** | | | **AREG concentration pg/ml** | | |  | | | |
| --- | --- | --- | --- | --- | --- | --- | --- | --- | --- | --- |
|  | **OD-1** | **OD-2** | **OD-3** | **concentration-1** | **concentration-2** | **concentration-3** | **T-Test analysis** | | | |
| THP-1 | 0.112 | 0.111 | 0.113 | 0.879 | 0.557 | 1.200 | THP-1 | vs | M0 | 2.26E-07 |
| M0 | 0.475 | 0.467 | 0.458 | 117.572 | 115.000 | 112.107 | THP-1 | vs | M1 | 3.51E-10 |
| M1 | 0.871 | 0.874 | 0.878 | 244.874 | 245.839 | 247.125 | THP-1 | vs | M2 | 3.78E-08 |
| M2 | 0.843 | 0.846 | 0.864 | 235.873 | 236.838 | 242.624 | M1 | vs | M2 | 2.73E-02 |

**Table S13.**

**Number and diameter statistics of organoids in AREG-treatment and control groups.**

| **AREG>100μm** | **AREG>40μm** | **NC>100μm** | **NC>40μm** |
| --- | --- | --- | --- |
| 7 | 28 | 2 | 30 |
| 7 | 25 | 4 | 23 |
| 10 | 26 | 2 | 25 |

**Table S14. IHC results in mouse model**

|  | **IHC results of Mouse p-p38** | | | | | | | | | | | |
| --- | --- | --- | --- | --- | --- | --- | --- | --- | --- | --- | --- | --- |
|  | intensity 1 | positive rate 1 | IHC Score 1（%） | intensity 2 | positive rate 2 | IHC Score 2（%） | intensity 3 | positive rate 3 | IHC Score 3（%） | Mean of IHC Score(%) | SEM of IHC Score |  |
| wt | 0 | 0% | 0 | 0 | 0% | 0 | 0.5 | 45% | 22.5 | 7.5 | 7.5 |  |
| CEA-SV40 | 1 | 60% | 60 | 1 | 60% | 60 | 1.5 | 25% | 37.5 | 52.5 | 7.5 |  |
| anti-AREG | 0.5 | 55% | 27.5 | 0.5 | 60% | 30 | 0.5 | 35% | 17.5 | 25.0 | 3.8 |  |
| FK866 | 0.5 | 15% | 7.5 | 0.5 | 50% | 25 | 0.5 | 50% | 25 | 19.2 | 5.8 |  |
| anti-AREG+  FK866 | 0.5 | 25% | 12.5 | 0.5 | 50% | 25 | 0.5 | 30% | 15 | 17.5 | 3.8 |  |

|  | **IHC results of Mouse p-STAT1** | | | | | | | | | | |
| --- | --- | --- | --- | --- | --- | --- | --- | --- | --- | --- | --- |
|  | intensity 1 | positive rate 1 | IHC Score 1（%） | intensity 2 | positive rate 2 | IHC Score 2（%） | intensity 3 | positive rate 3 | IHC Score 3（%） | Mean of IHC Score(%) | SEM of IHC Score |
| wt | 0 | 0% | 0 | 0.5 | 5% | 2.5 | 0 | 0% | 0 | 0.8 | 0.8 |
| CEA-SV40 | 1 | 10% | 10 | 1 | 20% | 20 | 1 | 20% | 20 | 16.7 | 3.3 |
| anti-AREG | 0.5 | 10% | 5 | 0.5 | 10% | 5 | 0.5 | 5% | 2.5 | 4.2 | 0.8 |
| FK866 | 0.5 | 5% | 2.5 | 0.5 | 5% | 2.5 | 0 | 0% | 0 | 1.7 | 0.8 |
| anti-AREG+  FK866 | 0.5 | 5% | 2.5 | 0.5 | 5% | 2.5 | 0.5 | 3% | 1.5 | 2.2 | 0.3 |

|  | **IHC results of Mouse p-NF-kB p65** | | | | | | | | | | |
| --- | --- | --- | --- | --- | --- | --- | --- | --- | --- | --- | --- |
|  | intensity 1 | positive rate 1 | IHC Score 1（%） | intensity 2 | positive rate 2 | IHC Score 2（%） | intensity 3 | positive rate 3 | IHC Score 3（%） | Mean of IHC Score(%) | SEM of IHC Score |
| wt | 0.5 | 5% | 2.5 | 0.5 | 10% | 5 | 0.5 | 5% | 2.5 | 3.3 | 0.8 |
| CEA-SV40 | 1 | 15% | 15 | 1 | 10% | 10 | 1 | 15% | 15 | 13.3 | 1.7 |
| anti-AREG | 1 | 10% | 10 | 0.5 | 15% | 7.5 | 0.5 | 5% | 2.5 | 6.7 | 2.2 |
| FK866 | 0.5 | 5% | 2.5 | 0.5 | 2% | 1 | 0.5 | 15% | 7.5 | 3.7 | 2.0 |
| anti-AREG+  FK866 | 0.5 | 15% | 7.5 | 0.5 | 10% | 5 | 0.5 | 2% | 1 | 4.5 | 1.9 |

|  | **IHC results of Mouse ITGA2** | | | | | | | | | | |
| --- | --- | --- | --- | --- | --- | --- | --- | --- | --- | --- | --- |
|  | intensity 1 | positive rate 1 | IHC Score 1（%） | intensity 2 | positive rate 2 | IHC Score 2（%） | intensity 3 | positive rate 3 | IHC Score 3（%） | Mean of IHC Score(%) | SEM of IHC Score |
| wt | 2 | 60% | 120 | 2 | 70% | 140 | 2 | 70% | 140 | 133.3 | 6.7 |
| CEA-SV40 | 3 | 90% | 270 | 3 | 95% | 285 | 3 | 95% | 285 | 280.0 | 5.0 |
| anti-AREG | 2 | 80% | 160 | 2 | 90% | 180 | 2 | 90% | 180 | 173.3 | 6.7 |
| FK866 | 2 | 85% | 170 | 2 | 75% | 150 | 2 | 65% | 130 | 150.0 | 11.5 |
| anti-AREG+  FK866 | 2 | 75% | 150 | 2 | 70% | 140 | 2 | 85% | 170 | 153.3 | 8.8 |

|  | **IHC results of Mouse CAPN8** | | | | | | | | | | |
| --- | --- | --- | --- | --- | --- | --- | --- | --- | --- | --- | --- |
|  | intensity 1 | positive rate 1 | IHC Score 1（%） | intensity 2 | positive rate 2 | IHC Score 2（%） | intensity 3 | positive rate 3 | IHC Score 3（%） | Mean of IHC Score(%) | SEM of IHC Score |
| wt | 1.5 | 60% | 90 | 1.5 | 70% | 105 | 1 | 40% | 40 | 78.3 | 19.6 |
| CEA-SV40 | 2 | 90% | 180 | 2 | 90% | 180 | 2 | 90% | 180 | 180.0 | 0.0 |
| anti-AREG | 2 | 70% | 140 | 2 | 85% | 170 | 2 | 90% | 180 | 163.3 | 12.0 |
| FK866 | 1.5 | 80% | 120 | 2 | 85% | 170 | 2 | 80% | 160 | 150.0 | 15.3 |
| anti-AREG+  FK866 | 1.5 | 75% | 112.5 | 1.5 | 80% | 120 | 1.5 | 85% | 127.5 | 120.0 | 4.3 |

|  | **IHC results of Mouse RNASE1** | | | | | | | | | | |
| --- | --- | --- | --- | --- | --- | --- | --- | --- | --- | --- | --- |
|  | intensity 1 | positive rate 1 | IHC Score 1（%） | intensity 2 | positive rate 2 | IHC Score 2（%） | intensity 3 | positive rate 3 | IHC Score 3（%） | Mean of IHC Score(%) | SEM of IHC Score |
| wt | 1.5 | 20% | 30 | 1.5 | 40% | 60 | 2 | 90% | 180 | 90.0 | 45.8 |
| CEA-SV40 | 2 | 55% | 110 | 2 | 45% | 90 | 2 | 40% | 80 | 93.3 | 8.8 |
| anti-AREG | 2 | 60% | 120 | 1.5 | 60% | 90 | 2 | 80% | 160 | 123.3 | 20.3 |
| FK866 | 2 | 10% | 20 | 1.5 | 40% | 60 | 2 | 30% | 60 | 46.7 | 13.3 |
| anti-AREG+  FK866 | 2 | 45% | 90 | 1.5 | 80% | 120 | 2 | 60% | 120 | 110.0 | 10.0 |

|  | **IHC results of Mouse LGR4** | | | | | | | | | | |
| --- | --- | --- | --- | --- | --- | --- | --- | --- | --- | --- | --- |
|  | intensity 1 | positive rate 1 | IHC Score 1（%） | intensity 2 | positive rate 2 | IHC Score 2（%） | intensity 3 | positive rate 3 | IHC Score 3（%） | Mean of IHC Score(%) | SEM of IHC Score |
| wt | 2 | 90% | 180 | 2 | 90% | 180 | 2 | 85% | 170 | 176.7 | 3.3 |
| CEA-SV40 | 3 | 95% | 285 | 3 | 90% | 270 | 3 | 95% | 285 | 280.0 | 5.0 |
| anti-AREG | 2 | 90% | 180 | 2 | 95% | 190 | 2 | 95% | 190 | 186.7 | 3.3 |
| FK866 | 1.5 | 90% | 135 | 2 | 90% | 180 | 2 | 90% | 180 | 165.0 | 15.0 |
| anti-AREG+  FK866 | 2 | 90% | 180 | 2 | 90% | 180 | 1.5 | 80% | 120 | 160.0 | 20.0 |

|  | **IHC results of Mouse AKR1B10** | | | | | | | | | | |
| --- | --- | --- | --- | --- | --- | --- | --- | --- | --- | --- | --- |
|  | intensity 1 | positive rate 1 | IHC Score 1（%） | intensity 2 | positive rate 2 | IHC Score 2（%） | intensity 3 | positive rate 3 | IHC Score 3（%） | Mean of IHC Score(%) | SEM of IHC Score |
| wt | 2 | 80% | 160 | 2 | 90% | 180 | 2 | 60% | 120 | 153.3 | 17.6 |
| CEA-SV40 | 2 | 80% | 160 | 2 | 50% | 100 | 2 | 30% | 60 | 106.7 | 29.1 |
| anti-AREG | 1.5 | 80% | 120 | 2 | 60% | 120 | 2 | 80% | 160 | 133.3 | 13.3 |
| FK866 | 2 | 80% | 160 | 2 | 80% | 160 | 2 | 80% | 160 | 160.0 | 0.0 |
| antiAREG+  FK866 | 2 | 60% | 120 | 1.5 | 40% | 60 | 1 | 60% | 60 | 80.0 | 20.0 |

|  | **IHC results of Mouse LCN2** | | | | | | | | | | |
| --- | --- | --- | --- | --- | --- | --- | --- | --- | --- | --- | --- |
|  | intensity 1 | positive rate 1 | IHC Score 1（%） | intensity 2 | positive rate 2 | IHC Score 2（%） | intensity 3 | positive rate 3 | IHC Score 3（%） | Mean of IHC Score(%) | SEM of IHC Score |
| wt | 2 | 90% | 180 | 2 | 90% | 180 | 2 | 90% | 180 | 180.0 | 0.0 |
| CEA-SV40 | 2 | 85% | 170 | 2 | 90% | 180 | 2 | 90% | 180 | 176.7 | 3.3 |
| anti-AREG | 1.5 | 80% | 120 | 2 | 90% | 180 | 2 | 80% | 160 | 153.3 | 17.6 |
| FK866 | 1.5 | 90% | 135 | 1.5 | 30% | 45 | 1.5 | 80% | 120 | 100.0 | 27.8 |
| antiAREG+  FK866 | 1.5 | 80% | 120 | 2 | 90% | 180 | 2 | 90% | 180 | 160.0 | 20.0 |

|  | **IHC results of Mouse Vimentin** | | | | | | | | | | |
| --- | --- | --- | --- | --- | --- | --- | --- | --- | --- | --- | --- |
|  | intensity 1 | positive rate 1 | IHC Score 1（%） | intensity 2 | positive rate 2 | IHC Score 2（%） | intensity 3 | positive rate 3 | IHC Score 3（%） | Mean of IHC Score(%) | SEM of IHC Score |
| wt | 2 | 15% | 30 | 2 | 10% | 20 | 2 | 5% | 10 | 20.0 | 5.8 |
| CEA-SV40 | 3 | 30% | 90 | 3 | 25% | 75 | 3 | 25% | 75 | 80.0 | 5.0 |
| anti-AREG | 2 | 25% | 50 | 2 | 25% | 50 | 2 | 15% | 30 | 43.3 | 6.7 |
| FK866 | 2 | 20% | 40 | 2 | 20% | 40 | 2 | 10% | 20 | 33.3 | 6.7 |
| anti-AREG+  FK866 | 2 | 15% | 30 | 2 | 15% | 30 | 2 | 10% | 20 | 26.7 | 3.3 |

|  | **IHC results of Mouse E-cad** | | | | | | | | | | |
| --- | --- | --- | --- | --- | --- | --- | --- | --- | --- | --- | --- |
|  | intensity 1 | positive rate 1 | IHC Score 1（%） | intensity 2 | positive rate 2 | IHC Score 2（%） | intensity 3 | positive rate 3 | IHC Score 3（%） | Mean of IHC Score(%) | SEM of IHC Score |
| wt | 2 | 85% | 170 | 2 | 85% | 170 | 2 | 70% | 140 | 160.0 | 10.0 |
| CEA-SV40 | 2 | 65% | 130 | 2 | 80% | 160 | 2 | 60% | 120 | 136.7 | 12.0 |
| anti-AREG | 2 | 75% | 150 | 2 | 85% | 170 | 2 | 90% | 180 | 166.7 | 8.8 |
| FK866 | 2 | 90% | 180 | 2 | 80% | 160 | 2 | 95% | 190 | 176.7 | 8.8 |
| anti-AREG+  FK866 | 2 | 75% | 150 | 2 | 80% | 160 | 2 | 80% | 160 | 156.7 | 3.3 |

**References**

[1]Butler A, et al. Integrating single-cell transcriptomic data across different conditions, technologies, and species. Nature biotechnology, 2018, 36(5): 411-420.

[2]Korsunsky I, et al. Fast, sensitive and accurate integration of single-cell data with Harmony. Nat Methods, 2019, 16(12): 1289-1296.

[3]Yu G, Wang L-G, Han Y and He Q-Y. clusterProfiler: an R package for comparing biological themes among gene clusters. Omics: a journal of integrative biology, 2012, 16(5): 284-287.

[4]Baird J R, et al. Evaluation of Explant Responses to STING Ligands: Personalized Immunosurgical Therapy for Head and Neck Squamous Cell Carcinoma. Cancer Res, 2018, 78(21): 6308-6319.
