# Supplementary figures and images for: Spatiotemporal multi-omics analysis uncovers NAD-dependent immunosuppressive niche triggering early gastric cancer
Source: Signal Transduct Target Ther. 2025 Sep 22;10:313. doi: 10.1038/s41392-025-02390-w (PMC12451012; doi:10.1038/s41392-025-02390-w)

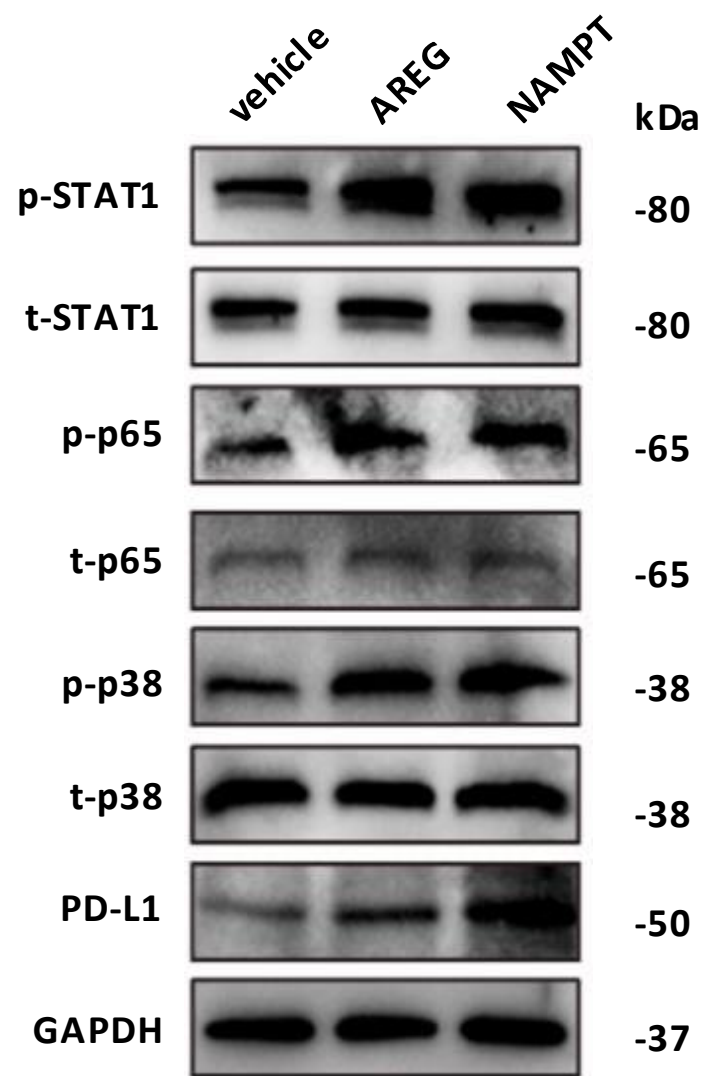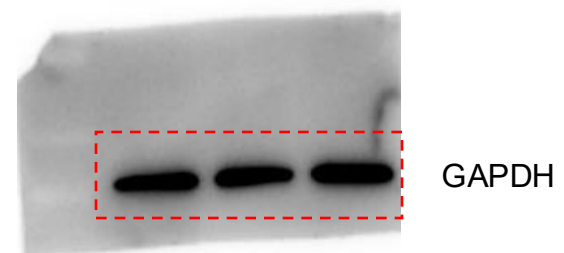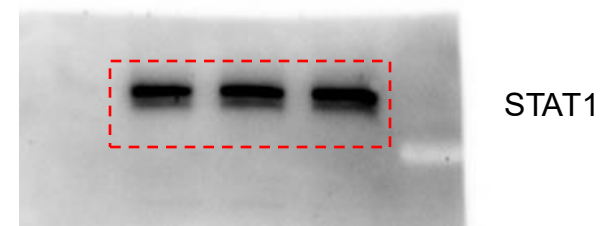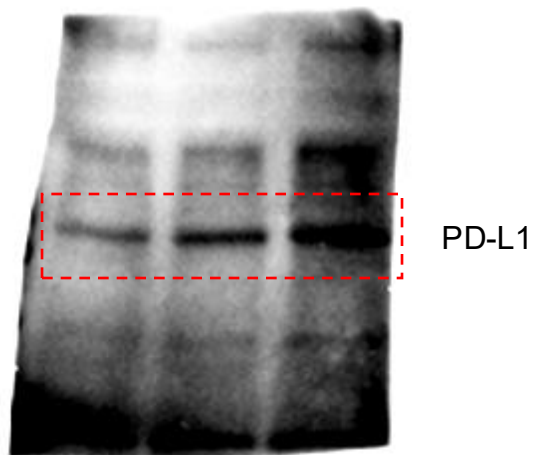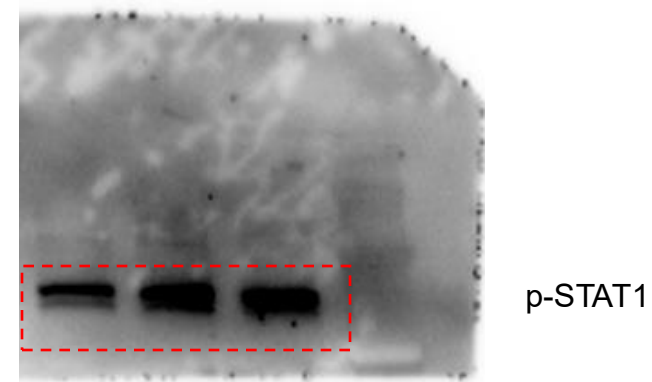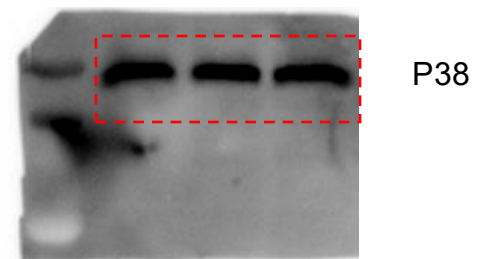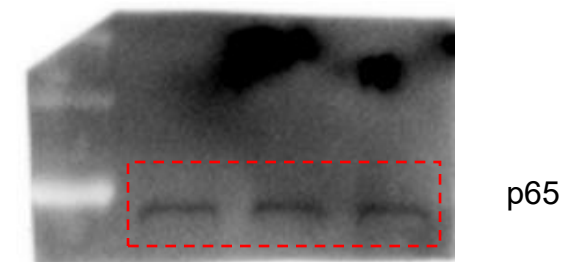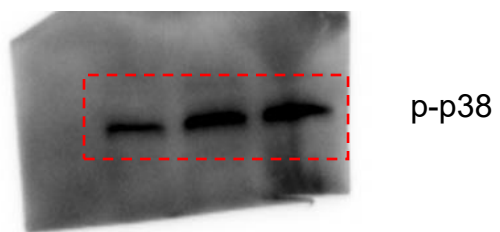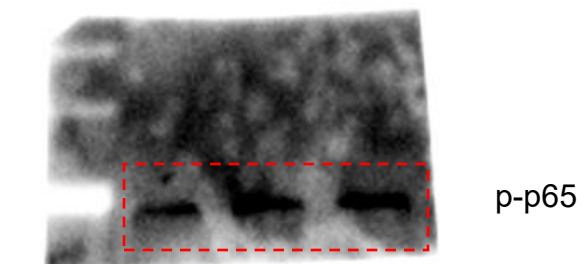

Supplement: Supplementary file 2 — All original and uncropped films of Western blots [file 41392_2025_2390_MOESM2_ESM.pdf]

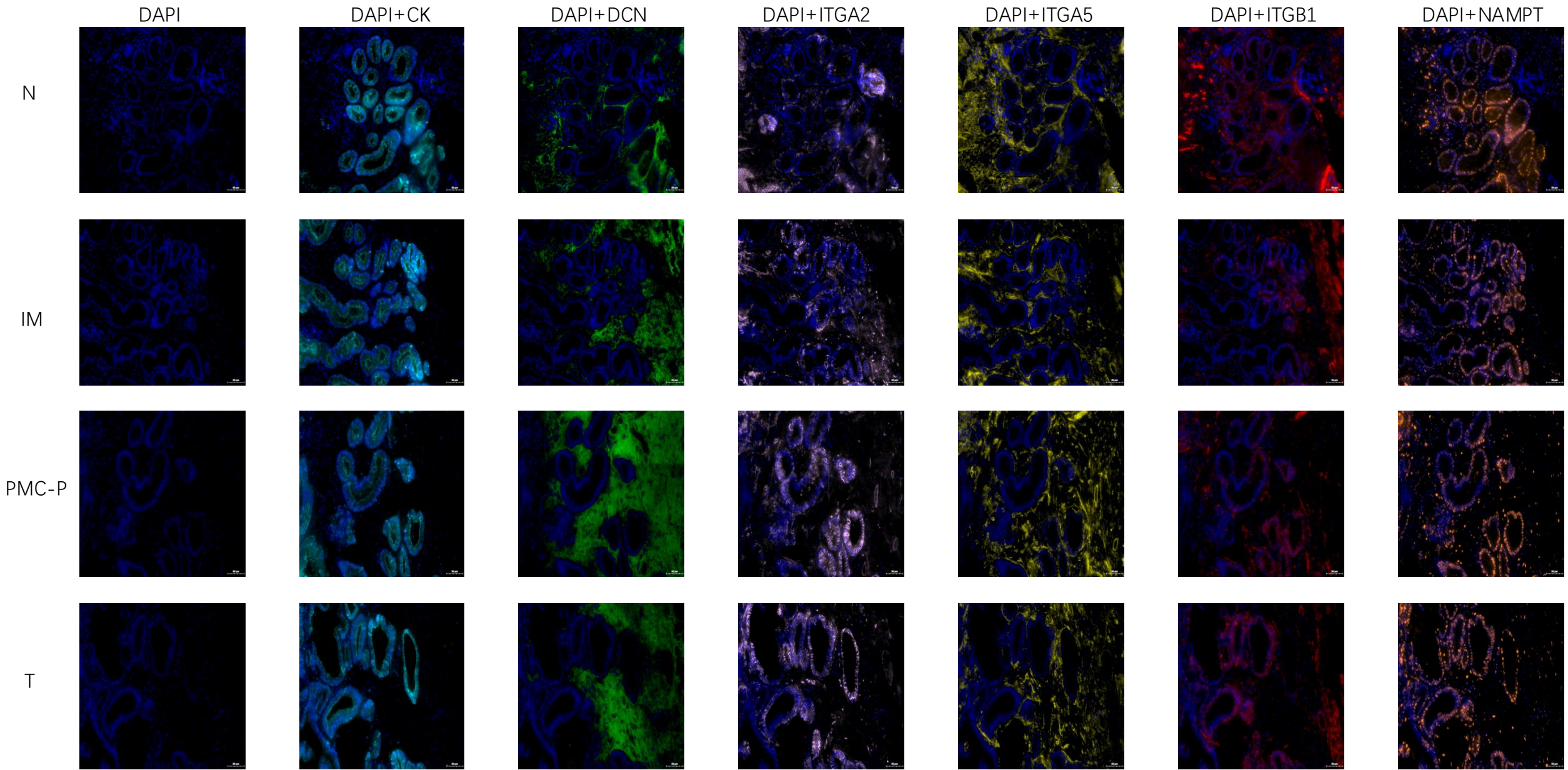

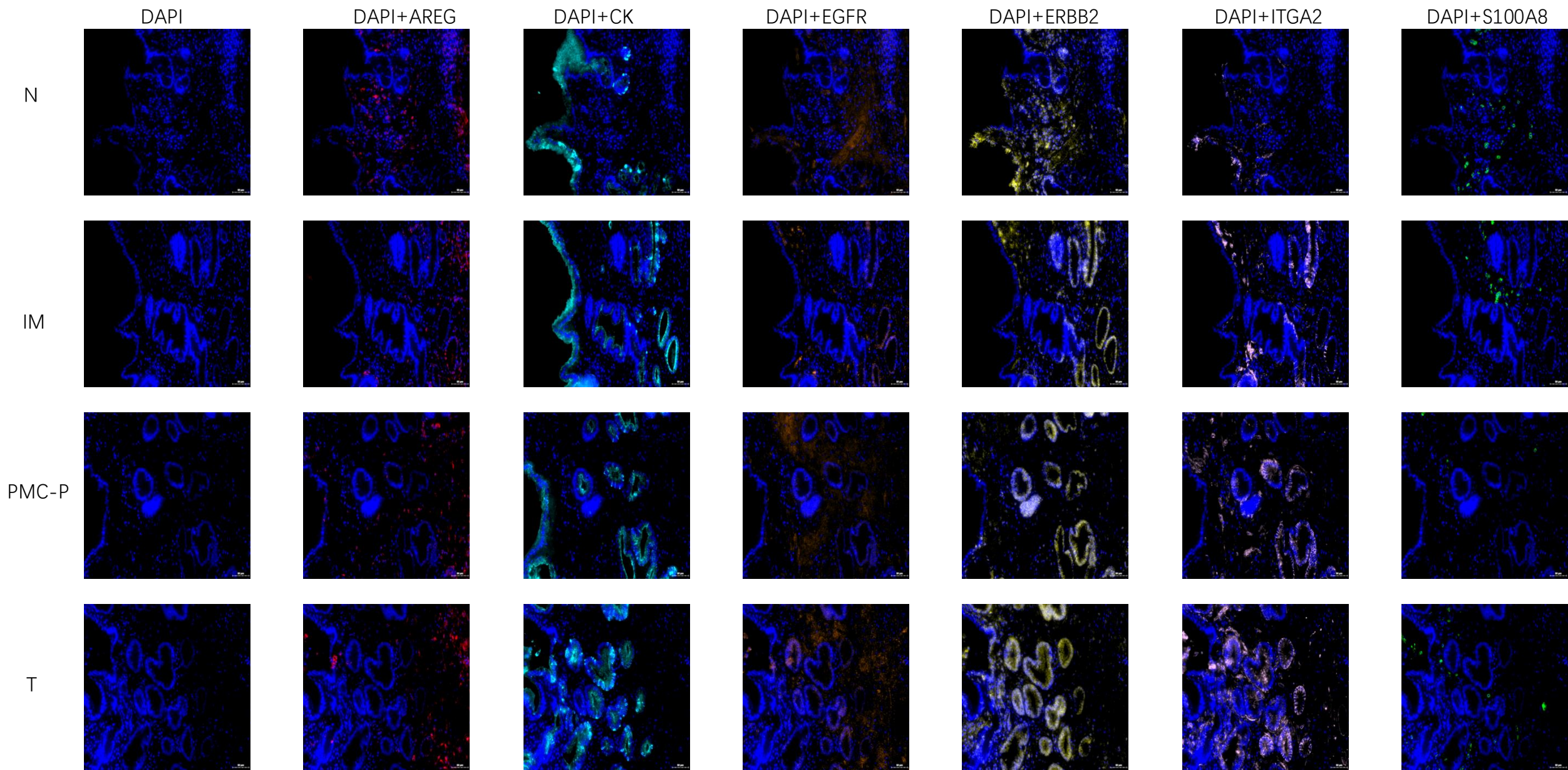

Supplement: Supplementary file 4 — All single-channel fluorescence images for Figures 6 and 8 [file 41392_2025_2390_MOESM4_ESM.pdf]
