# Supplementary material for: Spatiotemporal multi-omics analysis uncovers NAD-dependent immunosuppressive niche triggering early gastric cancer
Source: Signal Transduct Target Ther. 2025 Sep 22;10:313. doi: 10.1038/s41392-025-02390-w (PMC12451012; doi:10.1038/s41392-025-02390-w)
Supplement: Supplementary file 3 — Version 1 and 2 raw data of Figure 8f [file 41392_2025_2390_MOESM3_ESM.pdf]

名称	创建日期
Mouse gastric -ITGA2 -IHC- Interpretive data and statistical data-20241128 v1.xlsx	2024年11月28日 16:36
Mouse gastric -IHC- Interpretive data and statistical data-20241106 v1.xlsx	2025年2月11日 16:15
Mouse gastric -IHC- Interpretive data and statistical data-v2.docx	2025年4月18日 16:07

Figure 8f Raw Data Version 1

	p-STAT1(Interpretation of nuclear positive expression)				
	intensity 1	positive rate 1	IHC Score 1 (%)	intensity 2	positive rate 2
wt	0	0%	0	0 5	5%
CEA-SV40	1	10%	10	1	20%
anti-AREG	0 5	10%	5	0 5	10%
FK866	0 5	5%	2 5	0 5	5%
anti-AREG+FK866	0 5	5%	2 5	0 5	5%

				p-p38 1(Interpretation of nuclear positive expressi		
IHC Score 2 （%)	intensity 3	positive rate 3	IHC Score 3 （%)	intensity 1	positive rate 1	IHC Score 1 （%
2 5	0	0%	0	0	0%	0
20	1	20%	20	1	60%	60
5	0 5	5%	2 5	0 5	55%	27 5
2 5	0	0%	0	0 5	15%	7 5
2 5	0 5	3%	1 5	0 5	25%	12 5

on）						p-NF-kB p65
intensity 2	positive rate 2	IHC Score 2 （%	intensity 3	positive rate 3	IHC Score 3 （%	intensity 1
0	0%	0	0 5	45%	22 5	0 5
1	60%	60	1 5	25%	37 5	1
0 5	60%	30	0 5	35%	17 5	1
0 5	50%	25	0 5	50%	25	0 5
0 5	50%	25	0 5	30%	15	0 5

positive rate 1	IHC Score 1 （%	intensity 2	positive rate 2	IHC Score 2 （%	intensity 3	positive rate 3
5%	2 5	0 5	10%	5	0 5	5%
15%	15	1	10%	10	1	15%
10%	10	0 5	15%	7 5	0 5	5%
5%	2 5	0 5	2%	1	0 5	15%
15%	7 5	0 5	10%	5	0 5	2%

	PD-L1					
IHC Score 3（%	intensity 1	positive rate 1	IHC Score 1（%	intensity 2	positive rate 2	IHC Score 2（%
2 5	1 5	90%	135	1 5	80%	120
15	1 5	90%	135	1 5	90%	135
2 5	2	90%	180	1 5	90%	135
7 5	1 5	90%	135	1	50%	50
1	1	90%	90	1	90%	90

			RNASE1			
intensity 3	positive rate 3	IHC Score 3 （%	intensity 1	positive rate 1	IHC Score 1 （%	intensity 2
1 5	90%	135	1 5	20%	30	1 5
2	80%	160	2	55%	110	2
1 5	90%	135	2	60%	120	1 5
2	90%	180	2	10%	20	1 5
1 5	90%	135	2	45%	90	1 5

					AKR1B10	
positive rate 2	IHC Score 2 （%	intensity 3	positive rate 3	IHC Score 3 （%	intensity 1	positive rate 1
40%	60	2	90%	180	2	80%
45%	90	2	40%	80	2	80%
60%	90	2	80%	160	1 5	80%
40%	60	2	30%	60	2	80%
80%	120	2	60%	120	2	60%

IHC Score 1（%	intensity 2	positive rate 2	IHC Score 2（%	intensity 3	positive rate 3	IHC Score 3（
160	2	90%	180	2	60%	120
160	2	50%	100	2	30%	60
120	2	60%	120	2	80%	160
160	2	80%	160	2	80%	160
120	1 5	40%	60	1	60%	60

CAPN8					
intensity 1	positive rate 1	IHC Score 1 （%）	intensity 2	positive rate 2	IHC Score 2 （%
1 5	60%	90	1 5	70%	105
2	90%	180	2	90%	180
2	70%	140	2	85%	170
1 5	80%	120	2	85%	170
1 5	75%	112 5	1 5	80%	120

			LCN2(NGAL)			
intensity 3	positive rate 3	IHC Score 3 （%	intensity 1	positive rate 1	IHC Score 1 （%）	intensity 2
1	40%	40	2	90%	180	2
2	90%	180	2	85%	170	2
2	90%	180	1 5	80%	120	2
2	80%	160	1 5	90%	135	1 5
1 5	85%	127 5	1 5	80%	120	2

					LGR4	
positive rate 2	IHC Score 2 （	intensity 3	positive rate 3	IHC Score 3 （%)	intensity 1	positive rate 1
90%	180	2	90%	180	2	90%
90%	180	2	90%	180	3	95%
90%	180	2	80%	160	2	90%
30%	45	1 5	80%	120	1 5	90%
90%	180	2	90%	180	2	90%

IHC Score 1（%	intensity 2	positive rate 2	IHC Score 2（%	intensity 3	positive rate 3	IHC Score 3（%
180	2	90%	180	2	85%	170
285	3	90%	270	3	95%	285
180	2	95%	190	2	95%	190
135	2	90%	180	2	90%	180
180	2	90%	180	1.5	80%	120

E-cad							
intensity 1	positive rate	IHC Score 1 （%	intensity 2	positive rate	IHC Score 2 （%	intensity 3	positive rate
2	85%	170	2	85%	170	2	70%
2	65%	130	2	80%	160	2	60%
2	75%	150	2	85%	170	2	90%
2	90%	180	2	80%	160	2	95%
2	75%	150	2	80%	160	2	80%

	N-cad						
IHC Score 3 （%	intensity 1	positive rate	IHC Score 1	intensity 2	positive rate	IHC Score 2 （%	intensity 3
140	0	0%	0	0	0%	0	0
120	0	0%	0	0	0%	0	0
180	0	0%	0	0	0%	0	0
190	0	0%	0	0	0%	0	0
160	0	0%	0	0	0%	0	0

positive rate	IHC Score 3（%）
0%	0
0%	0
0%	0
0%	0
0%	0

	p-STAT1(Interpretation of nuclear positive expression）				p-p38 1(Interpretation of nuclear	
	IHC Score 1 （%	IHC Score 2 （%	IHC Score 3 （%	Mean(%)	IHC Score 1 （%	IHC Score 2 （%
wt	0	2 5	0	0 83	0	0
CEA-SV40	10	20	20	16 67	60	60
anti-AREG	5	5	2 5	4 17	27 5	30
FK866	2 5	2 5	0	1 67	7 5	25
anti-AREG+	2 5	2 5	1 5	2 17	12 5	25

positive expression）		p-NF-kB p65				PD-L1
IHC Score 3 （%	Mean(%)	IHC Score 1 （%	IHC Score 2 （%	IHC Score 3 （%	Mean(%)	IHC Score 1 （%
22 50	7 50	2 5	5	2 5	3 33	135
37 5	52 50	15	10	15	13 33	135
17 5	25 00	10	7 5	2 5	6 67	180
25	19 17	2 5	1	7 5	3 67	135
15	17 50	7 5	5	1	4 50	90

			RNASE1			
IHC Score 2 （%	IHC Score 3 （%	Mean(%)	IHC Score 1 （%	IHC Score 2 （%	IHC Score 3 （%	Mean(%)
120	135	130 00	30	60	180	90 00
135	160	143 33	110	90	80	93 33
135	135	150 00	120	90	160	123 33
50	180	121 67	20	60	60	46 67
90	135	105 00	90	120	120	110 00

AKR1B10				CAPN8		
IHC Score 1 （%	IHC Score 2 （%	IHC Score 3 （	Mean(%)	IHC Score 1 （%）	IHC Score 2 （%	IHC Score 3 （%
160	180	120	153 33	90	105	40
160	100	60	106 67	180	180	180
120	120	160	133 33	140	170	180
160	160	160	160 00	120	170	160
120	60	60	80 00	112 5	120	127 5

	LCN2(NGAL)				LGR4	
Mean(%)	IHC Score 1 （	IHC Score 2	IHC Score 3 （%）	Mean(%)	IHC Score 1 （%	IHC Score 2 （%
78 33	180	180	180	180 00	180	180
180 00	170	180	180	176 67	285	270
163 33	120	180	160	153 33	180	190
150 00	135	45	120	100 00	135	180
120 00	120	180	180	160 00	180	180

		E-cad				N-cad
IHC Score 3 （%	Mean(%)	IHC Score 1 （%	IHC Score 2 （%	IHC Score 3 （%	Mean(%)	IHC Score 1
170	176 67	170	170	140	160 00	0
285	280 00	130	160	120	136 67	0
190	186 67	150	170	180	166 67	0
180	165 00	180	160	190	176 67	0
120	160 00	150	160	160	156 67	0

IHC Score 2（%	IHC Score 3（%	Mean(%)
0	0	0 00
0	0	0 00
0	0	0 00
0	0	0 00
0	0	0 00

	ITGA2(Interpretation of Cell membrane and cytoplasm positive expression）						
	intensity 1	positive rate	IHC Score 1	intensity 2	positive rate	C Score 2（%	intensity 3
wt	2	60%	120	2	70%	140	2
CEA-SV40	3	90%	270	3	95%	285	3
anti-AREG	2	80%	160	2	90%	180	2
FK866	2	85%	170	2	75%	150	2
anti-AREG+FK866	2	75%	150	2	70%	140	2

positive rate	C Score 3（%）
70%	140
95%	285
90%	180
65%	130
85%	170

	ITGA2(Interpretation of Cell membrane and cytoplasm positive expression）		
	IHC Score 1 （%）	IHC Score 2 （%）	IHC Score 3 （%）
wt	120	140	140
CEA-SV40	270	285	285
anti-AREG	160	180	180
FK866	170	150	130
anti-AREG+FK866	150	140	170

Figure 8f Raw Data Version 2

IHC results of Human *AREG*									
**SliceNumber**	**Normal**			**Para-tumor**			**Tumor**		
	**intensity**	**positive rate**	**IHC Score （%）**	**intensity**	**positive rate**	**IHC Score （%）**	**intensity**	**positive rate**	**IHC Score （%）**
24G110633-003	2	3%	6	3	10%	30	2	10%	20
24G111264-002	2	5%	10	3	5%	15	2	5%	10
24G114683-002	2	5%	10	3	5%	15	2	3%	6
24G114865-003	2	5%	10	3	5%	15	2	2%	4
24G115204-002	2	20%	40	2	40%	80	2	15%	30
24G120637-002	2	5%	10	2	20%	40	2	10%	20
24G121171-002	2	5%	10	3	15%	45	2	5%	10
24G121167-007	2	5%	10	2	5%	10	2	15%	30
24G121169-003	2	5%	10	2	15%	30	2	15%	30
24G123213-002	2	5%	10	3	25%	75	2	15%	30
24G124077-002	2	15%	30	2	20%	40	2	5%	10
24G124076-003	2	3%	6	2	10%	20	2	10%	20
24G124080-002	2	3%	6	2	10%	20	2	10%	20
24G124073-002	2	2%	4	2	5%	10	2	10%	20
24G124074-003	2	5%	10	2	10%	20	2	10%	20
24G125761-002	2	3%	6	2	10%	20	2	5%	10
24G125974-003	2	2%	4	2	2%	4	2	3%	6
24G125973-003	2	5%	10	2	5%	10	2	10%	20
Mean of IHC Score			11.2			27.7			17.6
SEM of IHC Score			2.1			5.1			2.1

IHC results of Human *ITGA2*									
SliceNumber	Normal			Para-tumor			Tumor		
	intensity	positive rate	IHC Score （%）	intensity	positive rate	IHC Score （%）	intensity	positive rate	IHC Score （%）
24G124077-002	2	80%	160	3	95%	285	2	90%	180
24G125973-003	2	15%	30	2	90%	180	2	90%	180
24G121169-003	2	30%	60	3	75%	225	2	90%	180
24G123213-002	3	10%	30	3	90%	270	3	80%	240
24G124076-003	2	20%	40	2	90%	180	2	90%	180
24G121167-007	3	10%	30	3	90%	270	2	80%	160
24G114683-002	2	90%	180	3	95%	285	2	80%	160
24G120637-002	2	60%	120	3	95%	285	2	90%	180
24G124073-002	2	70%	140	3	90%	270	3	60%	180
24G124074-003	3	25%	75	2	90%	180	2	90%	180
24G125761-002	2	45%	90	2	90%	180	2	90%	180
24G121171-002	2	45%	90	2	90%	180	3	65%	195
24G115204-002	2	80%	160	3	90%	270	2	90%	180
24G114865-003	3	20%	60	3	95%	285	2	80%	160
24G120284-003	2	80%	160	3	95%	285	2	95%	190
24G110633-003	2	45%	90	3	90%	270	2	50%	100
Mean of IHC Score			94.7			243.8			176.6
SEM of IHC Score			13.1			11.7			6.9

IHC results of Human *NAMPT*									
SliceNumber	Normal			Para-tumor			Tumor		
	intensity	positive rate	IHC Score （%）	intensity	positive rate	IHC Score （%）	intensity	positive rate	IHC Score （%）
24G124074-003	1.5	30%	45	2	90%	180	1.5	80%	120
24G110633-003	1.5	20%	30	2	40%	80	2	60%	120
24G114683-002	1.5	10%	15	2	20%	40	1.5	80%	120
24G121167-007	—	—	—	2	30%	60	1.5	80%	120
24G121171-002	1.5	20%	30	—	—	—	—	—	—
24G115204-002	1.5	30%	45	2	25%	50	1.5	80%	120
24G111264-002	1.5	10%	15	2	50%	100	1.5	80%	120
24G124076-003	—	—	—	2	40%	80	1.5	80%	120
24G115202-002	1.5	10%	15	2	20%	40	1.5	30%	45
24G124077-002	1.5	10%	15	2	30%	60	1.5	80%	120
24G124080-002	1.5	10%	15	2	20%	40	1.5	80%	120
24G125974-003	1.5	20%	30	—	—	—	—	—	—
24G125761-002	0	0%	0	—	—	—	—	—	—
24G120637-002	1.5	60%%		2	60%	120	1	60%	60
24G123213-002	1.5	30%	45	1.5	10%	15	1.5	35%	52.5
24G125973-003	2	15%	30	2	90%	180	2	90%	180
24G114865-003	0	0%	0	1.5	10%	15	1.5	40%	60
24G121169-003	1.5	40%	60	2	65%	130	1.5	60%	90
Mean of IHC Score			26.0			79.3			104.5
SEM of IHC Score			4.5			13.7			9.3

IHC results of Human *PDL1*

SliceNumber	Normal			Para-tumor			Tumor		
	intensity	positive rate	IHC Score (%)	intensity	positive rate	IHC Score (%)	intensity	positive rate	IHC Score (%)
24G111264-002	1.5	2%	3	0	0%	0	0	0%	0
24G120284-003	2	1%	2	0	0%	0	0	0%	0
24G124076-003	0	0%	0	2	2%	4	0	0%	0
24G125974-003	0	0%	0	0	0%	0	0	0%	0
24G125402-002	0	0%	0	0	0%	0	0	0%	0
24G125402-003	0	0%	0	0	0%	0	0	0%	0
24G125973-003	0	0%	0	0	0%	0	0	0%	0
24G125763-002	0	0%	0	0	0%	0	0	0%	0
24G125073-002	0	0%	0	0	0%	0	2	10%	20
24G121171-002	0	0%	0	0	0%	0	0	0%	0
24G124077-002	0	0%	0	2	2%	4	0	0%	0
24G124080-002	0	0%	0	0	0%	0	0	0%	0
24G110633-003	0	0%	0	0	0%	0	0	0%	0
24G115202-002	0	0%	0	0	0%	0	0	0%	0
24G120637-002	0	0%	0	2	10%	20	0	0%	0
24G123213-002	0	0%	0	0	0%	0	2	1%	2
24G121167-007	0	0%	0	0	0%	0	0	0%	0
24G114865-003	0	0%	0	0	0%	0	0	0%	0
24G115204-002	0	0%	0	0	0%	0	0	0%	0
24G114683-002	0	0%	0	2	1%	2	0	0%	0
24G121169-003	0	0%	0	0	0%	0	0	0%	0
24G124074-003	0	0%	0	0	0%	0	2	1%	2

*续上表									
Mean of IHC Score			0.2			1.4			1.1
SEM of IHC Score			0.2			0.9			0.9

	**IHC results of Mouse *p-p38***										
	intensity 1	positive rate 1	IHC Score 1 (%)	intensity 2	positive rate 2	IHC Score 2 (%)	intensity 3	positive rate 3	IHC Score 3 (%)	Mean of IHC Score(%)	SEM of IHC Score
wt	0	0%	0	0	0%	0	0.5	45%	22.5	7.5	7.5
CEA-SV40	1	60%	60	1	60%	60	1.5	25%	37.5	52.5	7.5
anti-AREG	0.5	55%	27.5	0.5	60%	30	0.5	35%	17.5	25.0	3.8
FK866	0.5	15%	7.5	0.5	50%	25	0.5	50%	25	19.2	5.8
anti-AREG+FK866	0.5	25%	12.5	0.5	50%	25	0.5	30%	15	17.5	3.8

	**IHC results of Mouse *p-STAT1***										
	intensity 1	positive rate 1	IHC Score 1 (%)	intensity 2	positive rate 2	IHC Score 2 (%)	intensity 3	positive rate 3	IHC Score 3 (%)	Mean of IHC Score(%)	SEM of IHC Score
wt	0	0%	0	0.5	5%	2.5	0	0%	0	0.8	0.8
CEA-SV40	1	10%	10	1	20%	20	1	20%	20	16.7	3.3
anti-AREG	0.5	10%	5	0.5	10%	5	0.5	5%	2.5	4.2	0.8
FK866	0.5	5%	2.5	0.5	5%	2.5	0	0%	0	1.7	0.8

anti-AREG+FK866	0.5	5%	2.5	0.5	5%	2.5	0.5	3%	1.5	2.2	0.3

	**IHC results of Mouse *p-NF-kB p65***										
	intensity 1	positive rate 1	IHC Score 1 (%)	intensity 2	positive rate 2	IHC Score 2 (%)	intensity 3	positive rate 3	IHC Score 3 (%)	Mean of IHC Score(%)	SEM of IHC Score
wt	0.5	5%	2.5	0.5	10%	5	0.5	5%	2.5	3.3	0.8
CEA-SV40	1	15%	15	1	10%	10	1	15%	15	13.3	1.7
anti-AREG	1	10%	10	0.5	15%	7.5	0.5	5%	2.5	6.7	2.2
FK866	0.5	5%	2.5	0.5	2%	1	0.5	15%	7.5	3.7	2.0
anti-AREG+FK866	0.5	15%	7.5	0.5	10%	5	0.5	2%	1	4.5	1.9

	**IHC results of Mouse *ITGA2***										
	intensity 1	positive rate 1	IHC Score 1 (%)	intensity 2	positive rate 2	IHC Score 2 (%)	intensity 3	positive rate 3	IHC Score 3 (%)	Mean of IHC Score(%)	SEM of IHC Score
wt	2	60%	120	2	70%	140	2	70%	140	133.3	6.7

CEA-SV40	3	90%	270	3	95%	285	3	95%	285	280.0	5.0
anti-AREG	2	80%	160	2	90%	180	2	90%	180	173.3	6.7
FK866	2	85%	170	2	75%	150	2	65%	130	150.0	11.5
anti-AREG+FK866	2	75%	150	2	70%	140	2	85%	170	153.3	8.8

	**IHC results of Mouse *Vimentin***										
	intensity 1	positive rate 1	IHC Score 1 (%)	intensity 2	positive rate 2	IHC Score 2 (%)	intensity 3	positive rate 3	IHC Score 3 (%)	Mean of IHC Score(%)	SEM of IHC Score
wt	2	15%	30	2	10%	20	2	5%	10	20.0	5.8
CEA-SV40	3	30%	90	3	25%	75	3	25%	75	80.0	5.0
anti-AREG	2	25%	50	2	25%	50	2	15%	30	43.3	6.7
FK866	2	20%	40	2	20%	40	2	10%	20	33.3	6.7
anti-AREG+FK866	2	15%	30	2	15%	30	2	10%	20	26.7	3.3

	**IHC results of Mouse *RNASE1***										
	intensity 1	positive rate 1	IHC Score 1 (%)	intensity 2	positive rate 2	IHC Score 2 (%)	intensity 3	positive rate 3	IHC Score 3 (%)	Mean of IHC Score(%)	SEM of IHC Score
wt	1.5	20%	30	1.5	40%	60	2	90%	180	90.0	45.8

CEA-SV40	2	55%	110	2	45%	90	2	40%	80	93.3	8.8
anti-AREG	2	60%	120	1.5	60%	90	2	80%	160	123.3	20.3
FK866	2	10%	20	1.5	40%	60	2	30%	60	46.7	13.3
anti-AREG+FK866	2	45%	90	1.5	80%	120	2	60%	120	110.0	10.0

	**IHC results of Mouse *CAPN8***										
	intensity 1	positive rate 1	IHC Score 1 (%)	intensity 2	positive rate 2	IHC Score 2 (%)	intensity 3	positive rate 3	IHC Score 3 (%)	Mean of IHC Score(%)	SEM of IHC Score
wt	1.5	60%	90	1.5	70%	105	1	40%	40	78.3	19.6
CEA-SV40	2	90%	180	2	90%	180	2	90%	180	180.0	0.0
anti-AREG	2	70%	140	2	85%	170	2	90%	180	163.3	12.0
FK866	1.5	80%	120	2	85%	170	2	80%	160	150.0	15.3
anti-AREG+FK866	1.5	75%	112.5	1.5	80%	120	1.5	85%	127.5	120.0	4.3

	IHC results of Mouse *LGR4*										
	intensity 1	positive rate 1	IHC Score 1 (%)	intensity 2	positive rate 2	IHC Score 2 (%)	intensity 3	positive rate 3	IHC Score 3 (%)	Mean of IHC Score(%)	SEM of IHC Score
wt	2	90%	180	2	90%	180	2	85%	170	176.7	3.3
CEA-SV40	3	95%	285	3	90%	270	3	95%	285	280.0	5.0
anti-AREG	2	90%	180	2	95%	190	2	95%	190	186.7	3.3
FK866	1.5	90%	135	2	90%	180	2	90%	180	165.0	15.0
anti-AREG+FK866	2	90%	180	2	90%	180	1.5	80%	120	160.0	20.0

	IHC results of Mouse *AKR1B10*										
	intensity 1	positive rate 1	IHC Score 1 (%)	intensity 2	positive rate 2	IHC Score 2 (%)	intensity 3	positive rate 3	IHC Score 3 (%)	Mean of IHC Score(%)	SEM of IHC Score
wt	2	80%	160	2	90%	180	2	60%	120	153.3	17.6
CEA-SV40	2	80%	160	2	50%	100	2	30%	60	106.7	29.1
anti-AREG	1.5	80%	120	2	60%	120	2	80%	160	133.3	13.3
FK866	2	80%	160	2	80%	160	2	80%	160	160.0	0.0

anti-AREG+FK866	2	60%	120	1.5	40%	60	1	60%	60	80.0	20.0

	**IHC results of Mouse *LCN2***										
	intensity 1	positive rate 1	IHC Score 1 (%)	intensity 2	positive rate 2	IHC Score 2 (%)	intensity 3	positive rate 3	IHC Score 3 (%)	Mean of IHC Score(%)	SEM of IHC Score
wt	2	90%	180	2	90%	180	2	90%	180	180.0	0.0
CEA-SV40	2	85%	170	2	90%	180	2	90%	180	176.7	3.3
anti-AREG	1.5	80%	120	2	90%	180	2	80%	160	153.3	17.6
FK866	1.5	90%	135	1.5	30%	45	1.5	80%	120	100.0	27.8
anti-AREG+FK866	1.5	80%	120	2	90%	180	2	90%	180	160.0	20.0

	**IHC results of Mouse *E-cad***										
	intensity 1	positive rate 1	IHC Score 1 (%)	intensity 2	positive rate 2	IHC Score 2 (%)	intensity 3	positive rate 3	IHC Score 3 (%)	Mean of IHC Score(%)	SEM of IHC Score

wt	2	85%	170	2	85%	170	2	70%	140	160.0	10.0
CEA-SV40	2	65%	130	2	80%	160	2	60%	120	136.7	12.0
anti-AREG	2	75%	150	2	85%	170	2	90%	180	166.7	8.8
FK866	2	90%	180	2	80%	160	2	95%	190	176.7	8.8
anti-AREG+FK866	2	75%	150	2	80%	160	2	80%	160	156.7	3.3
